# Supplementary material for: Disparities in Cervical Cancer Among LHS+ Women: A Primer for Medical Students
Source: MedEdPORTAL. 2024 Dec 24;20:11482. doi: 10.15766/mep_2374-8265.11482 (PMC11668185; doi:10.15766/mep_2374-8265.11482)
Supplement: Supplementary file 1 — Facilitator Guide.docxPowerPoint Presentation.pptxEvaluation Form.docxVideo.movVideo Script.docxCase Studies.docx [file mep_2374-8265.11482-s001.zip › C. Evaluation Form.docx]

**Pre-test:** 5 minutes

This pre-test should be given to the audience before the presentation to assess their baseline knowledge.

Please write a unique pin number (e.g. birthday date): ______________

1. Complete the following self-assessment:

| **Please rate how much CONFIDENCE**  **you have in your ability to…** | **No confidence**  **0** | **1** | **2** | **3** | **Complete confidence**  **4** |
| --- | --- | --- | --- | --- | --- |
| Obj 1: Describe cervical cancer epidemiology, risk factors, and prevention efforts. | 0 | 1 | 2 | 3 | 4 |
| Obj 2: Describe health disparities in cervical cancer among LHS+ women. | 0 | 1 | 2 | 3 | 4 |
| Obj 3: Identify the social determinants related to cervical cancer outcomes among LHS+ women. | 0 | 1 | 2 | 3 | 4 |
| Obj 4: Identify culturally competent communication skills needed to assess barriers in cervical cancer prevention among LHS+ women. | 0 | 1 | 2 | 3 | 4 |

1. Which of the following is not a risk factor for cervical cancer?
   1. Obesity
   2. Smoking
   3. Long-term use of oral contraceptives
   4. Family history of breast cancer
2. The HPV (Human papillomavirus) vaccine is licensed in the United States for women and men through age __ years.
   1. 26
   2. 35
   3. 45
   4. 56
3. Which of the following does not result in cervical cancer disparities among LHS+ women?
   1. Socioeconomic factors
   2. Criminality rates
   3. Access to healthcare
   4. Language and culture
4. Cultural barriers among LHS+ women include all the following, except:
   1. Religion
   2. Sexism
   3. Distance
   4. Customs
5. The RESPECT model is a widely used tool to:
   1. promote physicians’ awareness of their own cultural biases and develop rapport with patients from diverse cultural backgrounds.
   2. support the training of healthcare professionals on how to engage patients in healthcare decision making.
   3. check patient’s understanding by asking them to state in their own words what they need to know or do about their health.

**Post-test:** 5 minutes

This post-test should be given to the audience following the presentation to evaluate their understanding and retention of the material.

Please rewrite the same unique pin number used in your pre-test: ________________

1. Complete the following self-assessment:

| **Please rate how much CONFIDENCE you have in your ability to…** | **No confidence**  **0** | **1** | **2** | **3** | **Complete confidence**  **4** |
| --- | --- | --- | --- | --- | --- |
| Obj 1: Describe cervical cancer epidemiology, risk factors, and prevention efforts. | 0 | 1 | 2 | 3 | 4 |
| Obj 2: Describe health disparities in cervical cancer among LHS+ women. | 0 | 1 | 2 | 3 | 4 |
| Obj 3: Identify the social determinants related to cervical cancer outcomes among LHS+ women. | 0 | 1 | 2 | 3 | 4 |
| Obj 4: Identify culturally competent communication skills needed to assess barriers in cervical cancer prevention among LHS+ women. | 0 | 1 | 2 | 3 | 4 |

1. Which of the following is not a risk factor for cervical cancer?
   1. Obesity
   2. Smoking
   3. Long-term use of oral contraceptives
   4. Family history of breast cancer
2. The HPV vaccine is licensed in the United States for women and men through age __ years.
   1. 26
   2. 35
   3. 45
   4. 56
3. Which of the following does not result in cervical cancer disparities among LHS+ women?
   1. Socioeconomic factors
   2. Criminality rates
   3. Access to healthcare
   4. Language and culture
4. Cultural barriers among LHS+ women include all the following, except:
   1. Religion
   2. Sexism
   3. Distance
   4. Customs
5. The RESPECT model is a widely used tool to:
   1. promote physicians’ awareness of their own cultural biases and develop rapport with patients from diverse cultural backgrounds.
   2. support the training of healthcare professionals on how to engage patients in healthcare decision making.
   3. check patient’s understanding by asking them to state in their own words what they need to know or do about their health.

**Evaluation form**

1. On a scale from 1=poor to 5=excellent, how would you rate this workshop overall?

1 2 3 4 5

1. On a scale from 1=poor to 5=excellent, how would you rate the usefulness of the content?
2. 2 3 4 5
3. On a scale from 1=poor to 5=excellent, how would you rate the video and its discussion?
4. 2 3 4 5
5. On a scale from 1=poor to 5=excellent, how would you rate the case scenarios and its discussions?
6. 2 3 4 5
7. What did you like about this workshop?
8. What suggestions do you have to improve this workshop?
