# Supplementary material for: Disparities in Cervical Cancer Among LHS+ Women: A Primer for Medical Students
Source: MedEdPORTAL. 2024 Dec 24;20:11482. doi: 10.15766/mep_2374-8265.11482 (PMC11668185; doi:10.15766/mep_2374-8265.11482)
Supplement: Supplementary file 1 — Facilitator Guide.docxPowerPoint Presentation.pptxEvaluation Form.docxVideo.movVideo Script.docxCase Studies.docx [file mep_2374-8265.11482-s001.zip › E. Video Script.docx]

**Video Script**

This document contains the dialogue the characters carried out throughout the video, which lasts 10 minutes, considering its discussion.

**Title: First Encounter**

INT. DOCTOR'S OFFICE - DAY

Isabella enters the doctor's office, greeted by Dr. James, a middle-aged Caucasian doctor. He sits behind his desk, focused on his computer.

**DR. JAMES**

(looking up)

Isabella, how can I help you today?

**ISABELLA**

(worried) (with heavy Spanish accent)

Dr. James, I've been having some *síntomas* I want to get checked out.

**DR. JAMES**

(dismissively)

Where are you from?

**ISABELLA**

From Puerto Rico. I moved years ago to work. Why?

Dr. James gives her a quick glance, seeming disinterested.

**DR. JAMES**

(uninterested)

What symptoms are you experiencing?

**ISABELLA**

(nervously)

My periods are, uh, irregular, and sometimes *me duele*, uh, HURT a lot down there.

**DR. JAMES**

Have you had any pain “down there” during or after intercourse?

**ISABELLA**

(confused)

Um, *no sé*, I don’t know what that means.

**DR. JAMES**

(uncomfortable)

Intercourse? When you’re being intimate.

**ISABELLA**

(sighs, speaks anxiously)

Doctor, I’m sorry, Spanish please!

**DR. JAMES**

(visibly annoyed)

Do you have any family here that speaks english? *Familia?*

**ISABELLA**

(nods no)

**DR. JAMES**

(sighs, annoyed)

Give me a second, I guess I’ll call interpreter.

Dr. James calls a Telemedicine interpreter.

NOTE ON-SCREEN: *For purposes of the exercise, from this point on the patient will speak as if the interpreter were speaking for her.*

**DR. JAMES**

Have you had any unusual vaginal discharge?

**ISABELLA**

Yes, and it smells bad.

**DR. JAMES**

How about feeling unusually tired?

**ISABELLA**

Yes, it's been going on for a few months now, and it's starting to worry me.

Dr. James's eyes wander around the room, avoiding direct eye contact and periodically glancing at the computer screen.

**DR. JAMES**

(hesitant)

Well, irregular bleeding can be normal for some women. It sounds like you could have a UTI or vaginal infection, which is common in sexually active women. As for the fatigue it could just be stress related. Have you been feeling stressed lately?

**ISABELLA**

(concerned)

Yes, but I've never experienced anything like this before. I'm really worried it might be something serious.

Dr. James interrupts, dismissively.

**DR. JAMES**

Look, Isabella, I understand your concerns, but you're a young woman. These symptoms are often exaggerated. I'm confident it's nothing to worry about.

Isabella's face shows a mix of frustration and hurt.

**ISABELLA**

(firmly)

Dr. James, I appreciate your perspective, but I know my body, and something doesn't feel right. Can you please run some tests?

Dr. James sighs and looks at his watch, clearly annoyed.

**DR. JAMES**

(reluctant)

Fine, we can run some tests, just to put your mind at ease.

NOTE ON-SCREEN: *2 weeks later the pap results returned showing a high-grade squamous intraepithelial lesion.*

FADE OUT.
