# Supplementary material for: Disparities in Cervical Cancer Among LHS+ Women: A Primer for Medical Students
Source: MedEdPORTAL. 2024 Dec 24;20:11482. doi: 10.15766/mep_2374-8265.11482 (PMC11668185; doi:10.15766/mep_2374-8265.11482)
Supplement: Supplementary file 1 — Facilitator Guide.docxPowerPoint Presentation.pptxEvaluation Form.docxVideo.movVideo Script.docxCase Studies.docx [file mep_2374-8265.11482-s001.zip › F. Case Studies.docx]

**Case Studies**

**Case studies and discussions: 10 minutes**

This document contains three case studies of hypothetical LHS+ patients to discuss cervical cancer risk factors, potential barriers that may result in disparities, and communication skills needed to unveil and/or address identified barriers. Use after pre-test and power point discussion to address the audience understanding and clarify doubts.

**Case #1:**

Maria is a 40-year-old woman who presents with vaginal bleeding and pain after intercourse for 6 months. Vitals include temperature = 36.9⁰C, heart rate = 76 beats per minute, respiratory rate = 15 breaths per minute, blood pressure = 113/75 mm Hg, saturation: 99%, and BMI = 23. She was born in Central America and immigrated to the United States 4 years ago to join her husband. He is an agricultural worker in New Jersey and has been in the United States for 8 years now. Her father was killed in a fight and her mother died of an unspecified cancer at 43 years old. She has no allergies and takes no medications. She does not smoke, drink or use illicit drugs. She has been married for 23 years and has 4 healthy adult children (G5P4A1). Her surgical history includes an appendectomy. Findings from her physical exam and review of systems were unremarkable.

**Questions:**

1. What are the patient’s risk factors for cervical cancer?
2. What social determinants can be identified in this case that may contribute to disparities in incidence, morbidity, and mortality?
3. What questions would you ask the patient to find out more about the social determinants of health?

**Case #2:**

Ana is a 35-year-old woman who presents with abnormal uterine and vaginal bleeding and discharge for 9 months. Vitals include temperature = 36.7⁰C, heart rate = 72 beats per minute, respiratory rate = 13 breaths per minute, blood pressure = 110/72 mm Hg, saturation: 99%, and BMI = 18. She is allergic to penicillin, has no surgeries and takes no medications. She was born in the United States to foreign-born LHS+ parents. Her father was diagnosed with hypertension, diabetes, chronic kidney disease, and depression and her mother has Alzheimer’s disease. She smokes one pack a day, drinks, uses illicit drugs, and has multiple sex partners. Her 2 children (G5P2A3) have been under state custody the last 5 years after she lost her job as a waitress. Her medical history includes Hep C, HIV, depression and opioids abuse. Findings from her physical exam and review of systems were unremarkable, except for needle tracks and bruises in the left antecubital fossa.

**Questions:**

1. What are the patient’s risk factors for cervical cancer?
2. What social determinants can be identified in this case that may contribute to disparities in incidence, morbidity, and mortality?
3. What questions would you ask the patient to find out more about the social determinants of health?

**Case #3:**

Gabriela is a 37-year-old woman who presents with abnormal uterine and vaginal bleeding for 3 months. Vitals include temperature = 37.1⁰C, heart rate = 85 beats per minute, respiratory rate = 15 breaths per minute, blood pressure = 121/79 mm Hg, saturation: 99%, and BMI = 32. She reports no allergies or surgeries. Medications include a daily multivitamin, oral contraceptives, metformin and candesartan. She is a foreign-born LHS+ women that immigrated with her family 30 years ago. At 15 years old she returned and stayed in her native country for a few years due to family affairs and later in life due to work affairs. Both parents are alive and well. She smokes half a pack daily and drinks socially but does not use illicit drugs. Due to her engineering studies and work hours as an operator in a pharmaceutical company, her diet consists of fast food and microwaved meals. She defines herself as bisexual and is currently sexually active with her boyfriend. Her medical history includes diabetes mellitus type 2, hypertension, and recurrent vaginal and urinary tract infections. She reports no children (G0P0A0). Findings from her physical exam and review of systems were unremarkable, except for fatigue.

**Questions:**

1. What are the patient’s risk factors for cervical cancer?
2. What social determinants can be identified in this case that may contribute to disparities in incidence, morbidity, and mortality?
3. What questions would you ask the patient to find out more about the social determinants of health?
