# Supplementary material for: Disparities in Cervical Cancer Among LHS+ Women: A Primer for Medical Students
Source: MedEdPORTAL. 2024 Dec 24;20:11482. doi: 10.15766/mep_2374-8265.11482 (PMC11668185; doi:10.15766/mep_2374-8265.11482)
Supplement: Supplementary file 1 — Facilitator Guide.docxPowerPoint Presentation.pptxEvaluation Form.docxVideo.movVideo Script.docxCase Studies.docx [file mep_2374-8265.11482-s001.zip › A. Facilitator Guide.docx]

**Facilitator Instructional Guide**

This document contains detailed information about each topic covered in the PowerPoint presentation with instructions and talking points to help facilitators present to the audience and guide discussions.

**Overall Goals**

The goals of this module are to help medical students understand how social determinants results in disparities in cervical cancer incidence and mortality among Latina, Latino, Latinx, Latine, Hispanic or of Spanish Origin+ (LHS+) women in the United States (U.S.); and what communication skills are needed as future physicians to assess potential barriers in prevention efforts to improve their care, experience, and health outcomes. This module may be implemented by individuals from all backgrounds.

**Objectives**

1. Describe cervical cancer epidemiology, risk factors, and prevention efforts.
2. Describe health disparities in cervical cancer among LHS+ women.
3. Identify the social determinants related to cervical cancer outcomes among LHS+ women.
4. Recognize culturally competent communication skills needed to assess barriers in cervical cancer prevention among LHS+ women.

**Suggested Agenda and Timeline**

- Pre-test (5 minutes)
- Power point presentation
  - Understanding cervical cancer (5 minutes)
  - HPV and cervical cancer (5 minutes)
  - Disparity factors (10 minutes)
  - Patient-physician communication (10 minutes)
- Video and discussion (10 minutes)
- Case studies and discussion (10 minutes)
- Post-test (5 minutes)

**Handouts and Materials**

- Computer set up with audio capability and connection to projector
- Internet
- Evaluation form
- Power point presentation
- Video
- Video script (English)
- Case studies
- Facilitator guide

**Slide Instructions**

**Slide 1:** Title: *Disparities in Cervical Cancer Among LHS+ Women*

*The facilitators should introduce themselves to the audience.*

**Slide 2:** Agenda

*The facilitator will present the agenda for the educational intervention.*

**Slide 3:** Relevance of the module

*The facilitator will explain that the reason this educational intervention addresses cervical cancer is due to the 2022 AACR Cancer Disparity Progress Report, which states that the Hispanic population have higher incidence and mortality rates from* ***cervical,*** *stomach and liver cancer in comparison with non-Hispanic population^1^.*

**Slide 4:** Pre-test

*The facilitator will instruct participants to complete the pre-test. Please include QR code for evaluation form.*

**Slide 5:** Objectives

*The facilitator will present the objectives for the educational intervention.*

**Slide 6:** Understanding cervical cancer (transition slide)

**Slide 7:** Introduction

*The facilitator will give a brief introduction to cervical cancer by pointing out the following:*

- Cervical cancer is the 4^th^ most common cancer among women in U.S. ^2^ Breast cancer is the first, followed by colorectal and lung cancer.
- In 2020, the cervical cancer incidence was 25.6 per 100,000 women^3^. Jurisdictions with higher rates included Puerto Rico with 10.3 per 100,000 women, Oklahoma with 9.7 per 100,000 women, and West Virginia, Texas and Alabama with 8.9 per 100,000 women each.
- For the same year, cervical cancer mortality was 5.1 per 100,000 women^3^. Jurisdictions with higher rates included Oklahoma with 3.8 per 100,000 women, Mississippi and Alabama with 3.3 per 100,000 women each, and Texas with 2.9 per 100,000 women.
- The median age of diagnosis in U.S. is 47 years, with almost 50% of cases diagnosed under age 35 years ^4^.
- This disease develops slowly over time, beginning with a precancerous condition known as dysplasia, which may progress to SCC or adenocarcinoma ^4^.
- With proper prevention, screening and treatment we can reduce cervical cancer burden in our population.

**Slide 8:** Clinical presentation

*The facilitator will explain the clinical manifestations of cervical cancer^4^.*

- Often asymptomatic and diagnosed following routine screening and/or pelvic examination.
- Post-coital abnormal vaginal bleeding.
- Lower limb edema, flank pain and sciatica.
- Passage of urine through vaginal canal due to a vesicovaginal fistula product of cervical cancer invasion anteriorly towards the bladder wall.
- Passage of feces through vaginal canal due to a rectovaginal fistula product of cervical cancer invasion posteriorly towards the rectum.

**Slide 9:** Knowledge checkpoint

*The facilitator will ask the audience what cervical cancer risk factors they can remember.*

**Slide 10**: Risk factors

*The facilitator will discuss the following cervical cancer risk factors:*

- Smoking - Women who smoke are twice as likely than those who don’t smoke to get cervical cancer^5^. Tobacco products have been found in cervical mucus of women who smoke, which lead to DNA damage in cervical cells. Although LHS+ women smoke less than Non-Hispanic Whites, LHS+ women are less likely to receive smoking cessation counseling^6^. Finally, LHS+ women who smokes have fewer rates of health insurance coverage than NHW who smokes^7^.
- Sexual history - Becoming sexually active at a young age and having multiple sexual partners or a high-risk partner increases the risk of developing cervical cancer^5,8^. Adolescents tend to avoid using condom due to low awareness of transmission of HPV^9^.
- Obesity - Obese women are less likely to report being screened for cervical cancer than non-obese women, and LHS+ women have higher rates of obesity than Non-Hispanic White women^5,10,11^.
- Weak immune system (e.g. HIV) - In 2015, cervical cancer rates are higher among HIV patients due to a weak immune system, and LHS+ women have over 3 times higher incidence rates of HIV when compared with Non-Hispanic Whites^12-14^.
- Long-term use of oral contraceptives - Using oral contraceptives for more than 5 years increases the relative risk of cervical cancer from 1.5 to 3.3-fold^15, 16^. Steroid contraceptive hormones can increase the susceptibility of cervical cells to infection with high-risk HPV types by promoting its integration into the host DNA genome.
- Multiple full-term pregnancies - Women who have had 3 or more full term pregnancies have an increased risk of developing cervical cancer due to hormonal changes and weakened immune systems during pregnancy^5, 17^.
- Diet low in fruits and vegetables - Consuming a diet rich in vitamins C, E, A and folate may decrease the risk of cervical cancer because antioxidants can reduce HPV viral loads and shorten the duration and progression of disease^18, 19^. A study concluded that dark greens and yellow vegetables were related to lowered cervical cancer incidence.
- Family history^5, 20^
- Low rates of HPV vaccination^5^ - In 2019, 87.3% of LHS+ adolescents ages 13 to 17 had 3 doses of HPV vaccine, when compared with 93.8% of Non-Hispanic White adolescents^21^. For the same year, 35.7% of LHS+ women ages 19 to 26 had at least 1 dose of HPV vaccine when compared with 44.7% of Non-Hispanic White women.

**Slide 11:** HPV and cervical cancer (transitional slide)

**Slide 12:** HPV

*The facilitator will discuss what HPV is and how the virus is transmitted.*

- Human Papillomavirus, abbreviated HPV, is a non-enveloped icosahedral DNA virus from the *Papillomaviridae* family^22^.
- More than 200 types of HPV have been identified and differentiated based on their genomic sequence^22^.
- Each type of HPV displays tissue specificity, infecting cutaneous, mucosal or both epithelia^22^.
- The virus is transmitted through skin-to-skin or skin-to-mucosa contact^22^. The most common established method of transmission is sexual contact; nevertheless, studies have suggested non-sexual transmission such as horizontal (through fomites) and vertical (from mother to child), and self-inoculation^23^.
- HPV is the most common and infectious viral sexually transmitted infection (STI)^24^.
- Over 70% of sexually active women and men will become infected at some point in their lives^24^.

**Slide 13:** HPV and its connection to cervical cancer

*The facilitator will explain the most common types of HPV and how it infects the tissue.*

- Low-risk HPV types include 6 and 11; these typically cause warts and may go away without treatment^25^. They are responsible for 90% of anal warts and can also cause cervical dysplasia, but changes are not precancerous^26^.
- High-risk HPV types, such as 16 and 18, are considered the cause of 70% of all cervical cancers worldwide^27^.
- Progression to cervical cancer is dependent on 2 viral oncoproteins named E6 and E7, which interfere with tumor suppressor proteins p53 and retinoblastoma and lead to tumorigenesis^28, 29^.
- HPV is responsible for disordered epithelial growth, usually occurring at the basal layer of the squamocolumnar junction (also known as the transformation zone)^26^.

**Slide 14:** Screening methods

*The facilitator will discuss the screening test for HPV and related guidelines.*

- The Papanicolaou test, or pap smear, is the screening test for HPV and subsequently cervical cancer.
- According to the American College of Obstetricians and Gynecologists^30^:
  - Screening should begin at the age of 21
  - Individuals aged 21-29 should have a cytology every 3 years.
  - Primary high-risk HPV testing every 5 years can be considered for average-risk patients aged 25–29 years.
  - Patients aged 30-65 should have one of the followings:
    - Cytology alone every 3 years
    - Primary high-risk HPV testing every 5 years
    - Cytology and high-risk HPV testing every 5 years
  - Individuals over 65 years need no screening if prior results were negative.

**Slide 15:** HPV vaccine

*The facilitator will discuss HPV vaccination and related guidelines^31^.*

- Three vaccines prevent infection against HPV:
  - Gardasil
  - Gardasil 9
  - Cervarix
- Gardasil 9 is the only vaccine currently used in the United States.
- It protects against HPV types 6,11, 16, 18, 31, 33, 45, 52, and 58.
- Children ages 11-12 should be given two doses of the vaccine 6-12 months apart.
- Three doses are recommended for patients that started the series after the age of 15 and before 26.
- HPV vaccine is approved for people up to 45 years old; however, it should be discussed with a health care practitioner.
- According to the CDC, the percentage of cervical precancers caused by the HPV types most often linked to cervical cancer has dropped by 40% after vaccination.

**Slide 16:** Disparity factors (transitional slide)

**Slide 17:** Cervical cancer incidence and mortality by race and ethnicity

*The facilitator will discuss cervical cancer incidence and mortality by race and ethnicity^32^.*

- This table presents cervical cancer incidence and mortality by race and ethnicity. Data was retrieved from the National Cancer Institute, and rates are reported per 100,000 women.
- In 2020, Hispanic women had the highest incidence rate and second highest mortality rate of cervical cancer when compared with other races and ethnicities. Black or African American women have the highest mortality rate among all races and ethnicities.

**Slide 18:** Socioeconomic factors

*The facilitator will discuss socioeconomic factors related to disparities in cervical cancer incidence and mortality among LHS+ women.*

- There are socioeconomic factors that affect LHS+ patients in greater proportion when compared with Non-Hispanic Whites^33^:
  - For example, 33% of LHS+ individuals in U.S. are foreign-born when compared with only 4% of Non-Hispanic Whites. For instance, foreign-born women who spent less than 25% of their life in U.S. had a higher prevalence of being unscreened for cervical cancer.
  - 24% of LHS+ individuals are not proficient in English when compared with 11% of Non-Hispanic Whites.
  - 31% of LHS+ individuals who are 25 years old or older do not have a high school diploma or its equivalent when compared with 7% of Non-Hispanic Whites.
  - And 20% of LHS+ individuals live under the federal poverty level when compared with 10% of Non-Hispanic Whites.
- Studies have shown a decrease in cervical cancer incidence in LHS+ communities when socioeconomic factors improve ^33^. For instance, a high income and education level have a positive correlation with medical adherence.

**Slides 19:** Language barriers

*The facilitator will introduce the topic of language barriers.*

- In 2013, 12.5 million Hispanics said they speak English but rate their speaking ability as less than “very well”^34^. And an additional 3.2 million say they do not speak English at all. Together, these groups of Hispanics make up one-third (32%) of all Hispanics ages 5 and older.

**Slide 20:** Knowledge checkpoint

*The facilitator will ask the audience how language barriers impact healthcare.*

**Slide 21:** Language barriers: effects

*The facilitator will explain how language barriers impact healthcare and offer examples^35, 36^.*

- Language barriers negatively impact the quality of healthcare, patient safety, and satisfaction of medical professionals and patients.
- Spanish-speaking patients may exhibit lower rates of mentioning symptoms, feelings, expectations, and thoughts compared to English-speaking patients during clinical conversations, impacting patient-physician communication.
- LHS+ patients with limited English proficiency presents with challenges that difficult their health system journey. Examples include difficulty scheduling appointments, misidentification in medical records, difficulty receiving health education, and lack of compliance and adherence, all of which can result in poor health outcomes. For example, limited English proficiency is associated with lower adherence to oral diabetic medication and insulin.
- There is also the issue of perceived discrimination. Asking questions such as “Where are you from?” can shut down a conversation with an LHS+ patient because it can be interpreted as either trying to uncover a person’s immigration status or implying that they do not belong because they were not born in the country.
- Finally, a survey of 48 hospitals in the midwestern United States found that not all hospitals had patient materials translated into Spanish, professional interpreters, or means to validate the interpretation provided by family members, staff, or clinicians. While some health organizations provide interpretation, these services indirectly increase the cost of health services and increase the length of treatment visits.

**Slide 22**: Socioeconomic factors: The Hispanic Paradox

*The facilitator will explain the concept of Hispanic Paradox and its importance.*

- The Hispanic Paradox may be explained by the Salmon Bias; for some cancers, LHS+ populations have greater or similar survival rates than Non-Hispanic Whites because they return to their homeland after cancer diagnosis (mostly to be w/ their family)^37^. This renders the individual "statistically immortal" and thus artificially lowers mortality for LHS+ population in U.S.^38^ Can be noted that foreign-born women who spent less than 25% of their life in the United States had a higher prevalence of being unscreened for cervical cancer^39^.

**Slide 23:** Limited access to healthcare

*The facilitator will present statistics from LHS+ individuals in the United States regarding their access to healthcare^33^.*

- In the United States, 26% of all LHS+ individuals between 18-64 years have no insurance coverage: Among LHS+ patients over the age of 65, only 3% have no insurance coverage.
- 32% of foreign-born LHS+ individuals have no insurance coverage, compared with only 12% of U.S.-born individuals.
- Lastly, 1/4 of LHS+ individuals between 18-64 years do not have a usual source of medical care.
  - 30% of LHS+ men do not have a usual source of medical care.
  - 19% of LHS+ women do not have usual source of medical care.

**Slide 24:** Figure 3. *The facilitator should use this slide to expand on the previously discussed information on how language barriers affect the patient's experience^40^. The data on this slide should be used to draw emphasis on how language can profoundly impact different aspects of patient care.*

- In a survey from Pew Research Institute in 2021, half (52%) of LHS+ individuals said they had a negative healthcare experience, where they experienced one of the following:
  - They had to speak up to get proper care
  - Felt rushed by their provider
  - Were treated with less respect than other patients
  - Felt their pain was not taken seriously
  - Were looked down because of weight or eating habits
  - They felt they received a lower quality of care than other patients.
- Interestingly, 30% of LHS+ women reported that their health concerns and symptoms were not taken seriously. Younger LHS+ women reported more negative healthcare experiences when compared to older LHS+ women or LHS+ men. Although 58% of LHS+ individuals prefer LHS+ providers, there are only 5.8% U.S. active LHS+ physicians^41^.
- Given LHS+ physicians are scarce, current and future providers must be trained to become aware of their own cultural biases, establish rapport with patients from different cultural backgrounds, and develop cultural humility to achieve a successful physician-patient relationship and communication.

**Slide 25:** Cultural barriers

*The facilitator must discuss how cultural barriers might be an obstacle to cervical cancer awareness, treatment, and patient outcomes. The facilitator must refrain from making vague generalizations, all issues discussed must be presented based on literature reviewed evidence and must be presented as such. These issues are sensitive, and facilitators must prepare to avoid committing micro aggressions during the presentation. Once this is achieved, the facilitator can continue expanding on the following topics^35^:*

- The intersection of cultural traditions such as machismo, familism, and degree of acculturation may impact the healthcare experience of LHS+ individuals in the United States.
- Machismo generally refers to traditional cultural norms and attitudes related to masculinity in Hispanic and Latino cultures. It often includes values associated with traditional roles and expectations within family and societal contexts.
- Studies have examined how traditional gender roles and male-dominated norms may influence experiences of discrimination and medical mistrust among LHS+ patients.
- Machismo culture may impact healthcare experiences for LHS+ individuals, as evidenced by its association with lower rates of condom use among sexually active LHS+ patients.
- LHS+ patients may have unique social needs, including competing family priorities and feelings of loyalty to family, which can impact hypertension management and overall healthcare satisfaction.
- Faith may play a significant role in LHS+ communities, with participation in church groups/ministries.
- Additionally, having immigrant parents may be associated with higher levels of medical mistrust.

**Slide 26:** Lack of knowledge and awareness

*The facilitator should transition to discuss how the lack of knowledge and awareness about cervical cancer impacts health outcomes. Like previously, generalizations must be avoided. When discussing the following topics, the facilitator must relate back to the previously discussed topics of language and cultural barriers and its impact in the risk factors, prevention efforts, symptoms recognition, and diagnosis of cervical cancer.*

- Lack of knowledge and awareness of signs and symptoms of cervical cancer, such as pain or discomfort, is considered a personal barrier^35^.
- HPV vaccination awareness
- Rates of unscreened women varied by country of origin: Mexico (9.8%), Caribbean (14.6%)^35^.
- Foreign-born women were twice as likely as women born in the United States to have never received a Pap test for cervical cancer screening^42^.
- Patient health education significantly improves cervical cancer screening adherence^35^.

**Slide 27:** Patient-physician communication (transitional slide)

**Slide 28:** Patient-physician communication

*Suggested dialogue can be the following:*

The American College of Obstetricians and Gynecologists (ACOG) reviewed different interviewing techniques to improve effective and compassionate communication between patients and physicians^43^.

Successful doctor-patient relationship and communication can encourage open communication to obtain more complete information, enhance the prospect of a more accurate diagnosis, and facilitate appropriate counseling, thus potentially improving adherence to treatment plans that benefits long-term health.

Differences between physicians and patients, including culture, gender, race, and religion, can introduce bias into patient–physician communication.

Among communication tools reviewed, the RESPECT model is widely used to promote physicians’ awareness of their own cultural biases and to develop physicians’ rapport with patients from distinct cultural backgrounds.

**Slide 29:** The RESPECT model

This model includes the following 7 core elements: rapport, empathy, support, partnership, explanations, cultural competence, and trust^43-45^.

*The facilitator will explain and discuss the 7 core elements of the RESPECT mode. A fact sheet can be developed to be shared with participants as a paper copy or through a QR code included in the presentation.*

Rapport

- Connect on a social level.
- See the patient’s point of view.
- Consciously attempt to suspend judgement.
- Recognize and avoid making assumptions.

Empathy

- Remember that the patient has come to you for help.
- Seek out and understand the patient’s rationale for her behavior or illness.
- Verbally acknowledge and legitimize the patient’s feelings.

Support

- Ask about and try to understand barriers to care and compliance.
- Help the patient overcome barriers.
- Involve family members if appropriate.
- Reassure the patient you are and will be available to help.

Partnership

- Stress that you will be working together to address medical problems.
- Be flexible, offer options.
- Negotiate roles when necessary.

Explanations

- Check often for understanding.
- Use verbal clarification techniques.

Cultural Competence

- Respect the patient and their culture and beliefs.
- Understand that the patient’s view of you may be defined by ethnic or cultural stereotypes.
- Be aware of your own biases and preconceptions.
- Know your limitations in addressing medical issues across cultures.
- Understand your personal style and recognize when it may not be working with a given patient.

Trust

- Self-disclosure may be an issue for some patients who are not accustomed to Western medical approaches.
- Take the necessary time and consciously work to establish trust.

**Slide 30:** Video: First Encounter (transitional slide)

*Suggested dialogue can be the following:*

We are going to watch a video recreating a first encounter between a young, female, Latina woman and a male doctor. The purpose of this video is to portray some of the dismissive approaches which impair the identification and acknowledgement of risk factors and health disparities among LHS+ women. This video will set the stage for a discussion about the portrayed disparities, attitudes, and biases.

*Please make note of and mention the following disclaimer:*

This video portrays a hypothetical situation without basis on any factual encounter known to the actors, writers, producers, or any one person working on the project. The objective is to set in motion an honest conversation considering and validating the learners’ sentiments and reactions towards the situation presented. We encourage you, the learners, to share your views, including reactions based upon your own experiences, if you choose, with the assurance that this room provides a safe space for discussion.

**Slide 31:** Keep these in mind...

Please keep in mind the following questions while watching the video:

*Please read the prompts/questions:*

1. Please take note of any errors and gaps in the physician’s history-taking and interview. Why do you think the physician carries out the interview this way?
2. What social and physiologic risk factors are present which predispose this patient to cervical cancer?
3. What would you have done differently as the physician depicted in the video?

**Slide 32:** Video

*The facilitator will click on the link and show the video.*

**Slide 33:** Video discussion

*The facilitator will lead the discussion based on the listed questions. Potential discussion points/answers are displayed below:*

| **Discussion Questions** | **Potential Discussion Points** |
| --- | --- |
| Please take note of any errors and gaps in the physician’s history-taking and interview. Why do you think the physician carries out the interview this way? | The doctor did not obtain a complete history of present illness, made assumptions and generalizations about social and sexual history, and did not obtain obstetric or gynecologic history. The physician did not take the patient’s symptoms seriously. Any mention of abnormal bleeding, which patients may sometimes describe as an irregular period, could be associated with cancer. The doctor is avoidant and presents with uninterested body language with lack of eye contact and should have contacted an interpreter earlier during the encounter.  Microaggression^35, 36, 46^ asks where the patient is from without purpose or justification.  Due to socioeconomic and ethnic bias, the doctor assumes the patient is low-income and/or has low-paying insurance, is uneducated, exaggerating, and wasting his time. The doctor allows harmful stereotypes to influence patient treatment. |
| What social and/or physiologic risk factors are present which predispose this patient to cervical cancer? | Risk factors^5-21^, language barrier, immigrant without social support from loved ones, fear due to bias prevents the patient from seeking help earlier until symptoms cannot be ignored and interfere with daily life, sexual practices, family history of cancer, possible educational barriers which include achieved level of education and general awareness of cancer prevention, awareness of HPV vaccination, socio-cultural barriers such as taboos regarding sexual education, machismo, and religious beliefs. |
| What would you have done differently than the physician depicted in the video? | Present with attentive body language, make eye contact, complete HPI correctly as well as social history, sexual history etc. *(see question 1 discussion point);* contact an interpreter earlier, be patient, provide options to correct or lessen social determinants of health such as access to a social worker^47, 48^. Doctors should emphasize patient education about cervical cancer prevention including vaccination, and encourage the patient by asking questions such as “What worries you about your current symptoms?” and “What do you know about cervical cancer?” Remember to use open-ended questions that allow the patient to express their concerns and history, these questions will guide to an appropriate diagnosis, treatment, and promote patient compliance. |

*In question #1, we encourage the facilitator to ask the learners if any other phrases or words jump out at them as microaggressions. Remember to keep the conversation centered on the discussion points to provide ample time for the case studies. If the learners overlook any points the facilitator may guide the conversation or state, the points.*

**Slide 34:** Case studies (transitional slide)

*Suggested dialogue can be the following:*

Now we are going to discuss some case studies. The purpose of this exercise is to apply the gained knowledge and understanding of cervical cancer risk factors, potential barriers that may result in disparities, and communication skills needed as health professionals, to 3 case studies of LHS+ women.

**Slide 35:** Keep these in mind...

Please keep in mind the following questions while reading the case study:

*The facilitator will now read the following 3 questions and implore the learners to read the case studies with these questions in mind. The same 3 questions will be answered after each case study.*

1. What are the patient’s risk factors for cervical cancer?
2. What social determinants can be identified in this case that may contribute to disparities in incidence, morbidity, and mortality?
3. What questions would you ask the patient to find out more about the social determinants of health?

**Slide 36:** Case #1

*Please read the following case study or call for a volunteer:*

Maria is a 40-year-old woman who presents with vaginal bleeding and pain after intercourse for 6 months. Vitals include temperature = 36.9⁰C, heart rate = 76 beats per minute, respiratory rate = 15 breaths per minute, blood pressure = 113/75 mm Hg, saturation: 99%, and BMI = 23. She was born in Central America and immigrated to the United States 4 years ago to join her husband. He is an agricultural worker in Texas and has been in the United States for 8 years now. Her father was killed in a fight and her mother died of an unspecified cancer at 43 years old. She has no allergies and takes no medications. She does not smoke, drink or use illicit drugs. She has been married for 23 years and has 4 healthy adult children (G5P4A1). Her surgical history includes an appendectomy. Findings from her physical exam and review of systems were unremarkable.

*The facilitator will lead the discussion of case #1 based on the listed questions. Recommended discussion points/answers are displayed below:*

| **Discussion questions** | **Recommended discussion points/answers** |
| --- | --- |
| What are the patient’s risk factors for cervical cancer? | Multiple pregnancies^5, 17, 20^  Family history |
| What social determinants can be identified in this case that may contribute to disparities in incidence, morbidity, and mortality? | Immigrant status^34-36^  Language  Culture and religion  Lack of knowledge about cervical cancer and its prevention and screening  Low income  Health insurance status  Transportation  Even though religion is not directly mentioned in the case, LHS+ population may belong to religious groups or hold religious beliefs that can impact their comfort level when talking openly about sexual health topics. Religion can impact decisions such as diet, medicine, and the preferred gender of their health providers. Therefore, health care professionals are faced with the challenge of understanding and accommodating the patient’s religious needs/beliefs when evaluating and treating the patient.^37^ |
| What questions would you ask the patient to find out more about the social determinants of health? | Examples: Have you had access to a physician or had any screening test? What are some challenges when seeking healthcare (i.e., transportation, health insurance, communication)?^34, 44^ |

**Slide 37:** Case #2

*Please read the following case study:*

Ana is a 35-year-old woman who presents with abnormal uterine and vaginal bleeding and discharge for 9 months. Vitals include temperature = 36.7⁰C, heart rate = 72 beats per minute, respiratory rate = 13 breaths per minute, blood pressure = 110/72 mm Hg, saturation: 99%, and BMI = 18. She is allergic to penicillin, has no surgeries and takes no medications. She was born in the United States to foreign-born LHS+ parents. Her father was diagnosed with hypertension, diabetes, chronic kidney disease, and depression and her mother has Alzheimer’s disease. She smokes one pack a day, drinks, uses illicit drugs, and has multiple sex partners. Her 2 children (G5P2A3) have been under state custody the last 5 years after she lost her job as a waitress. Her medical history includes Hep C, HIV, depression and opioids abuse. Findings from her physical exam and review of systems were unremarkable, except for needle tracks and bruises in the left antecubital fossa.

*The facilitator will lead the discussion of Case #2 based on the listed questions. Recommended discussion points/answers are displayed below:*

| **Discussion questions** | **Recommended discussion points/answers** |
| --- | --- |
| What are the patient’s risk factors for cervical cancer? | Multiple sex partners^5-9, 12-14, 17-19^  HIV  Smoking  Multiple pregnancies  Poor diet |
| What social determinants can be identified in this case that may contribute to disparities in incidence, morbidity, and mortality? | Lack of knowledge about cervical cancer and its prevention and screening^34, 35^  Low income  Health insurance status  Transportation |
| What questions would you ask the patient to find out more about the social determinants of health? | Examples: Do you have access to treatment for your STDs (transportation or insurance)? Do you know the risk factors for cervical cancer and how to actively prevent it or screen for cervical cancer?^12-14, 34, 44^ |

**Slide 38:** Case #3

*Please read the following case study:*

Gabriela is a 37-year-old woman who presents with abnormal uterine and vaginal bleeding for 3 months. Vitals include temperature = 37.1⁰C, heart rate = 85 beats per minute, respiratory rate = 15 breaths per minute, blood pressure = 121/79 mm Hg, saturation: 99%, and BMI = 32. She reports no allergies or surgeries. Medications include a daily multivitamin, oral contraceptives, metformin and candesartan. She is a foreign-born LHS+ women that immigrated with her family 30 years ago. At 15 years old she returned and stayed in her native country for a few years due to family affairs and later in life due to work affairs. Both parents are alive and well. She smokes half a pack daily and drinks socially but does not use illicit drugs. Due to her engineering studies and work hours as an operator in a pharmaceutical company, her diet consists of fast food and microwaved meals. She defines herself as bisexual and is currently sexually active with her boyfriend. Her medical history includes diabetes mellitus type 2, hypertension, and recurrent vaginal and urinary tract infections. She reports no children (G0P0A0). Findings from her physical exam and review of systems were unremarkable, except for fatigue.

*The facilitator will lead the discussion of Case #3 based on the listed questions. Recommended discussion points/answers are displayed below:*

| **Discussion questions** | **Recommended discussion points/answers** |
| --- | --- |
| What are the patient’s risk factors for cervical cancer? | Obesity^5-7, 10, 11, 15, 16, 18, 19^  Prolonged use of oral contraceptives  Smoking  Poor diet |
| What social determinants can be identified in this case that may contribute to disparities in incidence, morbidity, and mortality? | Hispanic paradox / Salmon bias^35, 38, 39^  Lack of knowledge about cervical cancer and its prevention and screening |
| What questions would you ask the patient to find out more about the social determinants of health? | Examples: What do you know about cervical cancer prevention and screening opportunities offered? Do you know the potential risks of cervical cancer?^4, 5, 28-31, 44^ |

**Slide 39:** Key Takeaways

*The facilitator will share the following key takeaways:*

- LHS+ women have the highest incidence and second highest mortality of cervical cancer in U.S.
- These disparities are the result of socioeconomic factors, limited access to healthcare, language and cultural barriers, and negative experiences with health care providers.
- More than half of LHS+ women have had a negative experience with health care providers, including not taking their health concerns or symptoms seriously.
- Besides promoting a diverse workforce, every physician must be trained to become aware of the disparities presented, improve the rapport and communication with their LHS+ women patients, and become an advocate for their health and well-being.

**Slide 40:** Post-test

*The facilitator will instruct participants to complete the post-test. Please include QR code for evaluation form.*

**Slide 41:** Questions? (End of presentation)

**References**

1. Williams PA, Zaidi SK, Sengupta R. AACR Cancer Disparities Progress Report 2022. Cancer Epidemiology, Biomarkers & Prevention. 2022;31(7):1249-1250. doi:10.1158/1055-9965.epi-22-0542
2. Cervical cancer. World Health Organization. Accessed November 23, 2023. <https://www.who.int/news-room/fact-sheets/detail/cervical-cancer>
3. United States Cancer Statistics: Data Visualizations. Centers for Disease Control and Prevention. Accessed November 23, 2023. <https://gis.cdc.gov/Cancer/USCS/#/Demographics/>
4. Cohen PA, Jhingran A, Oaknin A, Denny L. Cervical cancer. The Lancet. 2019;393(10167):169-182. doi:10.1016/s0140-6736(18)32470-x
5. Risk factors for cervical cancer. Cervical Cancer Risk Factors. Published January 3, 2020. Accessed November 23, 2023. <https://www.cancer.org/cancer/types/cervical-cancer/causes-risks-prevention/risk-factors.html>
6. Kristman-Valente AN, Flaherty BP. Latino Cigarette Smoking Patterns by Gender in a US-National Sample. Ann Behav Med. 2016;50(1):34-47. doi:10.1007/s12160-015-9729-9
7. Hispanic and Latino people encounter barriers to quitting successfully. Centers for Disease Control and Prevention. Published June 27, 2022. Accessed November 23, 2023. <https://www.cdc.gov/tobacco/health-equity/hispanic-latino/quitting-tobacco.html>
8. Kashyap N, Krishnan N, Kaur S, Ghai S. Risk factors of Cervical Cancer: A Case-Control Study. Asia Pac J Oncol Nurs. 2019;6(3):308-314. <https://doi.org/10.4103/apjon.apjon_73_18>
9. Frega A, Stentella P, De Ioris A, et al. Young women, cervical intraepithelial neoplasia and human papillomavirus: risk factors for persistence and recurrence. Cancer Lett. 2003;196(2):127-134. doi:10.1016/s0304-3835(03)00218-0
10. Maruthur NM, Bolen SD, Brancati FL, Clark JM. The association of obesity and cervical cancer screening: a systematic review and meta-analysis. Obesity (Silver Spring). 2009;17(2):375-381. doi:10.1038/oby.2008.480
11. Fryar, CD, Carroll, MD, Ogden, CL. Prevalence of Overweight, Obesity, and Severe Obesity Among Adults Aged 20 and Over: United States, 1960–1962 Through 2015–2016. Publish September 5, 2018. Accessed November 23, 2023. https://www.cdc.gov/nchs/data/hestat/obesity_adult_15_16/obesity_adult_15_16.htm
12. Salihu H, Dongarwar D, Ikedionwu CA, et al. Racial/Ethnic Disparities in the Burden of HIV/Cervical Cancer Comorbidity and Related In-hospital Mortality in the USA. J Racial Ethn Health Disparities. 2021;8(1):24-32. doi:10.1007/s40615-020-00751-5
13. Ortiz AP, Engels EA, Nogueras-González GM, et al. Disparities in human papillomavirus-related cancer incidence and survival among human immunodeficiency virus-infected Hispanics living in the United States. Cancer. 2018;124(23):4520-4528. doi:10.1002/cncr.31702
14. Berman TA, Schiller JT. Human papillomavirus in cervical cancer and oropharyngeal cancer: One cause, two diseases. Cancer. 2017;123(12):2219-2229. doi:10.1002/cncr.30588
15. Gadducci A, Cosio S, Fruzzetti F. Estro-progestin Contraceptives and Risk of Cervical Cancer: A Debated Issue. Anticancer Res. 2020;40(11):5995-6002. doi:10.21873/anticanres.14620
16. Oral contraceptives (birth control pills) and cancer risk. National Cancer Institute. Published February 22, 2018. Accessed November 23, 2023. <https://www.cancer.gov/about-cancer/causes-prevention/risk/hormones/oral-contraceptives-fact-sheet#:~:text=In%20addition%2C%20oral%20contraceptives%20might,of%20virtually%20all%20cervical%20cancers>
17. Hinkula M, Pukkala E, Kyyrönen P, et al. A population-based study on the risk of cervical cancer and cervical intraepithelial neoplasia among grand multiparous women in Finland. Br J Cancer. 2004;90(5):1025-1029. doi:10.1038/sj.bjc.6601650
18. Atalah E, Urteaga C, Rebolledo A, Villegas RA, Medina E, Csendes A. Alimentación, tabaquismo e historia reproductiva como factores de riesgo del cáncer de cuello del útero [Diet, smoking and reproductive history as risk factor for cervical cancer]. Rev Med Chil. 2001;129(6):597-603.
19. Hajiesmaeil M, Mirzaei Dahka S, Khorrami R, et al. Intake of food groups and cervical cancer in women at risk for cervical cancer: A nested case-control study. Caspian J Intern Med. 2022;13(3):599-606. doi:10.22088/cjim.13.3.599
20. De M. Zelmanowicz A, Hildesheim A. Family history of cancer as a risk factor for cervical carcinoma: A review of the literature. Papillomavirus Report. 2004;15(3):113-120. doi: 10.1179/095741904225005115
21. Immunizations and Hispanic Americans. Office of Minority Health. Accessed November 23, 2023. <https://minorityhealth.hhs.gov/immunizations-and-hispanic-americans>
22. Graham SV. The human papillomavirus replication cycle, and its links to cancer progression: a comprehensive review. Clin Sci (Lond). 2017;131(17):2201-2221. Published 2017 Aug 10. doi:10.1042/CS20160786
23. Petca A, Borislavschi A, Zvanca ME, Petca RC, Sandru F, Dumitrascu MC. Non-sexual HPV transmission and role of vaccination for a better future (Review). Exp Ther Med. 2020;20(6):186. doi:10.3892/etm.2020.9316
24. Okunade KS. Human papillomavirus and cervical cancer [published correction appears in J Obstet Gynaecol. 2020 May;40(4):590]. J Obstet Gynaecol. 2020;40(5):602-608. doi:10.1080/01443615.2019.1634030
25. Crosbie EJ, Einstein MH, Franceschi S, Kitchener HC. Human papillomavirus and cervical cancer. The Lancet. 2013 Sep 7;382(9895):889-99. doi: 10.1016/S0140-6736(13)60022-7.
26. Huber J, Mueller A, Sailer M, Regidor PA. Human papillomavirus persistence or clearance after infection in reproductive age. What is the status? Review of the literature and new data of a vaginal gel containing silicate dioxide, citric acid, and selenite. Womens Health (Lond). 2021;17:17455065211020702. doi:10.1177/17455065211020702
27. Human Papillomavirus. World Health Organization. Accessed November 23, 2023. <https://www.who.int/teams/health-product-policy-and-standards/standards-and-specifications/vaccine-standardization/human-papillomavirus>
28. Pal A, Kundu R. Human Papillomavirus E6 and E7: The Cervical Cancer Hallmarks and Targets for Therapy. Front Microbiol. 2020;10:3116. Published 2020 Jan 21. doi:10.3389/fmicb.2019.03116
29. White EA. Manipulation of Epithelial Differentiation by HPV Oncoproteins. Viruses. 2019;11(4):369. Published 2019 Apr 22. doi:10.3390/v11040369
30. Updated cervical cancer screening guidelines. The American College of Obstetricians and Gynecologists. Published April 2021. Accessed November 23, 2023. <https://www.acog.org/clinical/clinical-guidance/practice-advisory/articles/2021/04/updated-cervical-cancer-screening-guidelines>
31. HPV vaccination recommendations. Centers for Disease Control and Prevention. Published November 16, 2021. Accessed November 23, 2023. <https://www.cdc.gov/vaccines/vpd/hpv/hcp/recommendations.html>.
32. Surveillance, Epidemiology, and End Results Program. National Cancer Institute. Accessed November 23, 2023. <https://seer.cancer.gov/statistics->network/explorer/application.html
33. Miller KD, Ortiz AP, Pinheiro PS, et al. Cancer statistics for the US Hispanic/Latino population, 2021. CA Cancer J Clin. 2021;71(6):466-487. doi:10.3322/caac.21695
34. DeNavas-Walt C, Proctor BD, Smith, JC. Income, Poverty, and Health Insurance Coverage in the United States: 2012. U.S. Census Bureau. Published September 2013. Accessed November 23, 2022. https://www.census.gov/library/publications/2013/demo/p60-245.html
35. Escobedo LE, Cervantes L, Havranek E. Barriers in Healthcare for Latinx Patients with Limited English Proficiency-a Narrative Review. J Gen Intern Med. 2023;38(5):1264-1271. doi:10.1007/s11606-022-07995-3
36. Al Shamsi H, Almutairi AG, Al Mashrafi S, Al Kalbani T. Implications of Language Barriers for Healthcare: A Systematic Review. Oman Med J. 2020;35(2):e122. Published 2020 Apr 30. doi:10.5001/omj.2020.40
37. Swihart DL, Yarrarapu SNS, Martin RL. Cultural Religious Competence in Clinical Practice. [Updated 2023 Jul 24]. In: StatPearls [Internet]. Treasure Island (FL): StatPearls Publishing; 2024 Jan-. Available from: https://www.ncbi.nlm.nih.gov/books/NBK493216/
38. Turra CM, Elo IT. The Impact of Salmon Bias on the Hispanic Mortality Advantage: New Evidence from Social Security Data. Popul Res Policy Rev. 2008;27(5):515-530. doi:10.1007/s11113-008-9087-4
39. Franzini L, Ribble JC, Keddie AM. Understanding the Hispanic paradox. *Ethn Dis*. 2001;11(3):496-518.
40. Tamayo LI, Perez F, Perez A, et al. Cancer screening and breast cancer family history in Spanish-speaking Hispanic/Latina women in California [published correction appears in Front Oncol. 2023 Jan 06;12:1087022]. Front Oncol. 2022;12:940162. Published 2022 Oct 26. doi:10.3389/fonc.2022.940162
41. Hispanic Americans’ Trust in and engagement with science. Pew Research Center. Published June 14, 2022. Accessed November 23, 2023. <https://www.pewresearch.org/science/2022/06/14/hispanic-americans-trust-in-and-engagement-with-science/>
42. Diversity in Medicine: Facts and Figures 2019. Association of American Medical Colleges. Accessed November 23, 2023. <https://www.aamc.org/data-reports/workforce/data/figure-18-percentage-all-active-physicians-race/ethnicity-2018#:~:text=Diversity%20in%20Medicine%3A%20Facts%20and%20Figures%202019,-Diversity%20in%20Medicine&text=Figure%2018%20shows%20the%20percentage,as%20Black%20or%20African%20American>
43. Endeshaw M, Clarke T, Senkomago V, Saraiya M. Cervical Cancer Screening Among Women by Birthplace and Percent of Lifetime Living in the United States. J Low Genit Tract Dis. 2018;22(4):280-287. doi:10.1097/LGT.0000000000000422
44. Effective Patient–Physician Communication. The American College of Obstetricians and Gynecologists. Published February 2014. Accessed November 23, 2023.
45. The Respect Model. U.S. Department of Health and Human Services. Accessed November 23, 2023. https://hclsig.thinkculturalhealth.hhs.gov/ProviderContent/PDFs/RESPECTModel.pdf
46. Respect Model. U.S. Department of Health and Human Services. Accessed November 23, 2023. https://thinkculturalhealth.hhs.gov/assets/pdfs/RESPECTModel_BH.pdf
47. Acholonu RG, Cook TE, Roswell RO, Greene RE. Interrupting Microaggressions in Health Care Settings: A Guide for Teaching Medical Students. MedEdPORTAL. 2020;16:10969. <https://doi.org/10.15766/mep_2374-8265.10969>
48. Goldberg C. Practical Guide to Clinical Medicine. University of California San Diego School of Medicine. Published Match 2020. Accessed November 23, 2023. <https://meded.ucsd.edu/clinicalmed/introduction.html>
49. Patient interview guide. Nova Southeastern University. Accessed November 23, 2023. <https://cfsecure.nova.edu/PEPrx/documents/evaluation_IPPE_COMMEE_II_MedicationProfile.pdf>
