# Supplementary material for: Disparities in Cervical Cancer Among LHS+ Women: A Primer for Medical Students
Source: MedEdPORTAL. 2024 Dec 24;20:11482. doi: 10.15766/mep_2374-8265.11482 (PMC11668185; doi:10.15766/mep_2374-8265.11482)
Supplement: Supplementary file 1 — Facilitator Guide.docxPowerPoint Presentation.pptxEvaluation Form.docxVideo.movVideo Script.docxCase Studies.docx [file mep_2374-8265.11482-s001.zip › B. PowerPoint Presentation.pptx]

## Slide 1
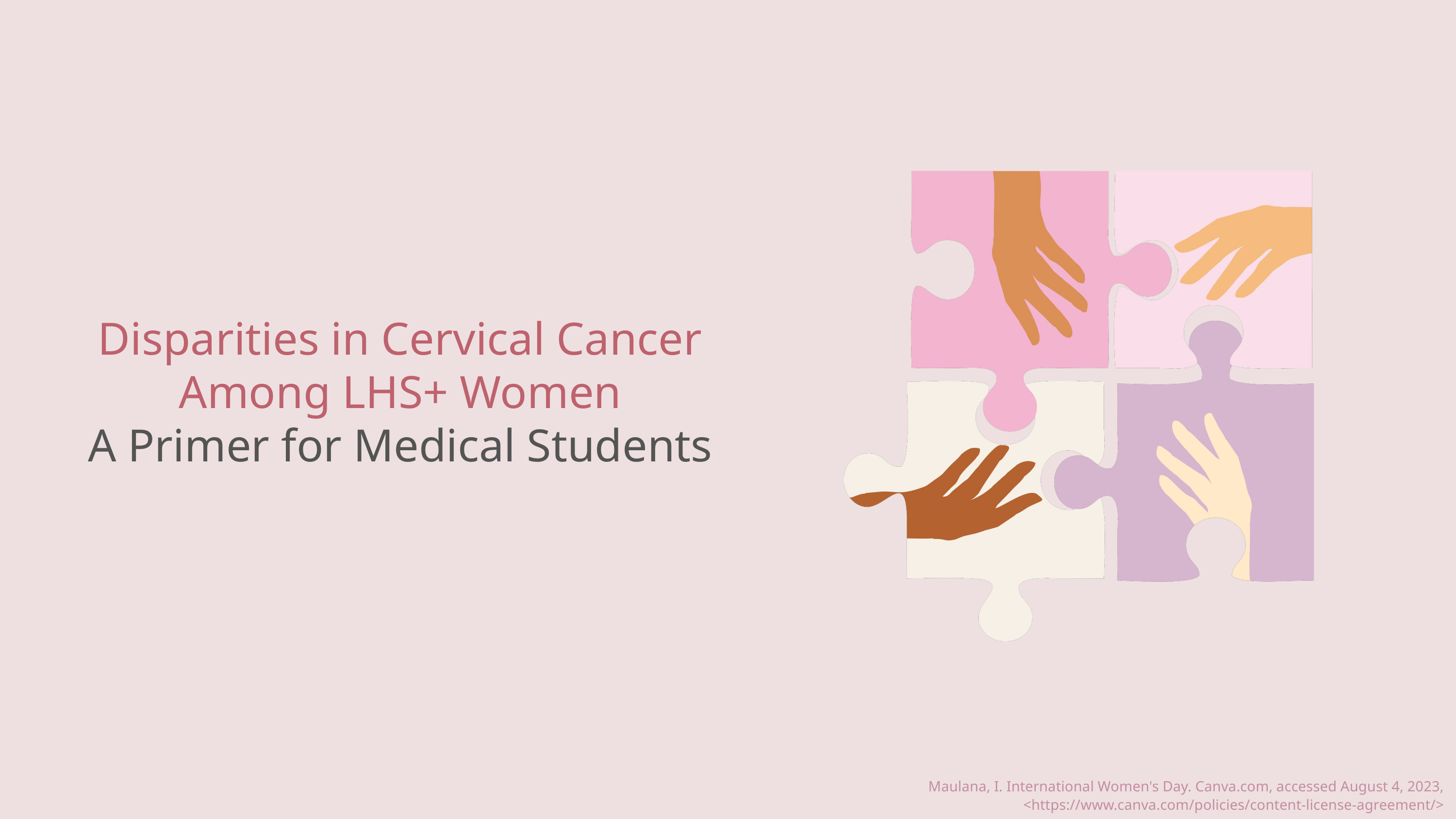

Disparities in Cervical Cancer Among LHS+ Women​
A Primer for Medical Students
Maulana, I. International Women's Day. Canva.com, accessed August 4, 2023, <https://www.canva.com/policies/content-license-agreement/>

## Slide 2
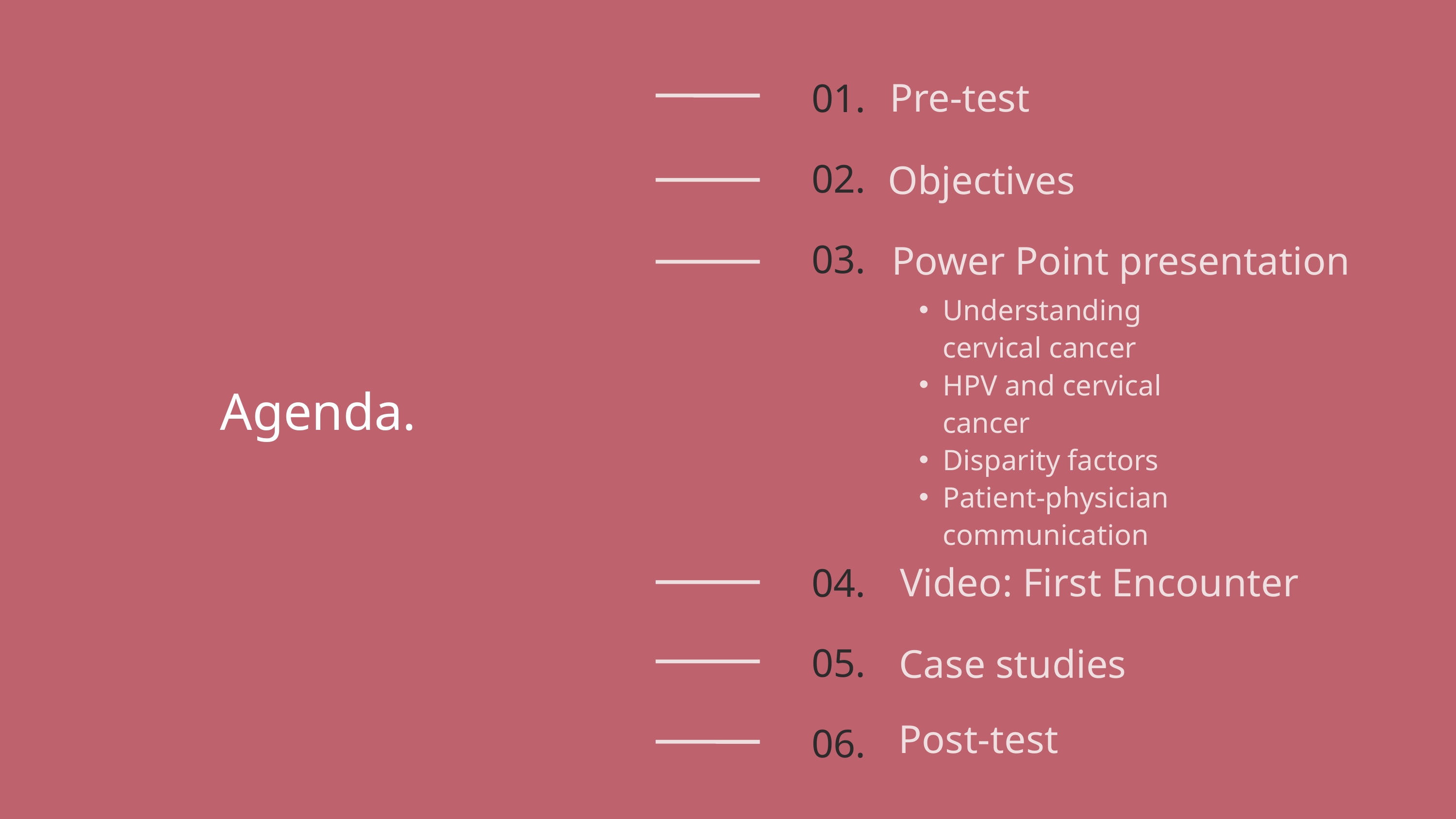

Pre-test
01.
02.
Objectives
03.
Power Point presentation
Understanding cervical cancer
HPV and cervical cancer
Disparity factors
Patient-physician communication
Agenda.
Video: First Encounter
04.
Case studies
05.
Post-test
06.

## Slide 3
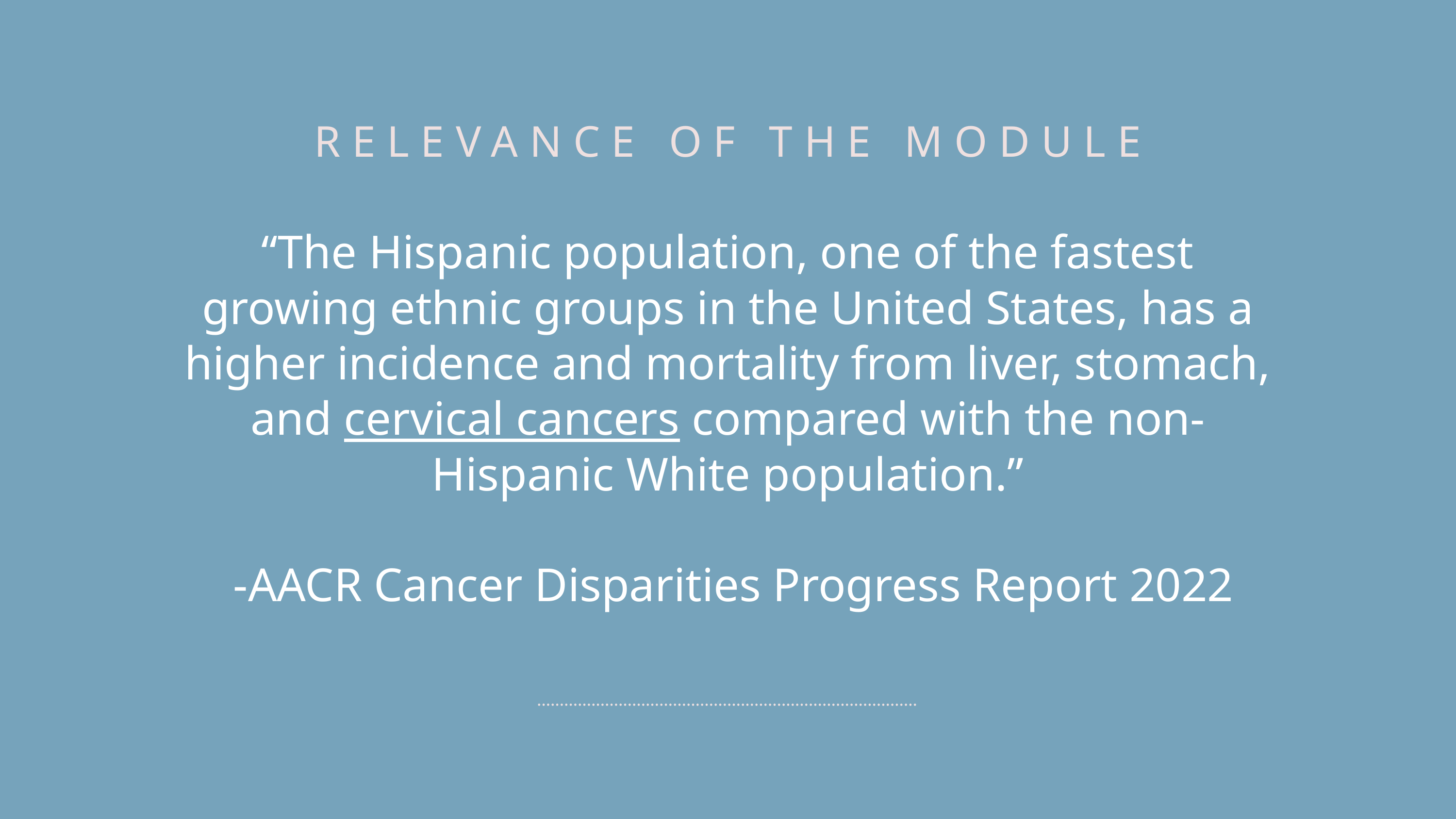

RELEVANCE OF THE MODULE
“The Hispanic population, one of the fastest growing ethnic groups in the United States, has a higher incidence and mortality from liver, stomach, and cervical cancers compared with the non-Hispanic White population.”
​
 -AACR Cancer Disparities Progress Report 2022

## Slide 4
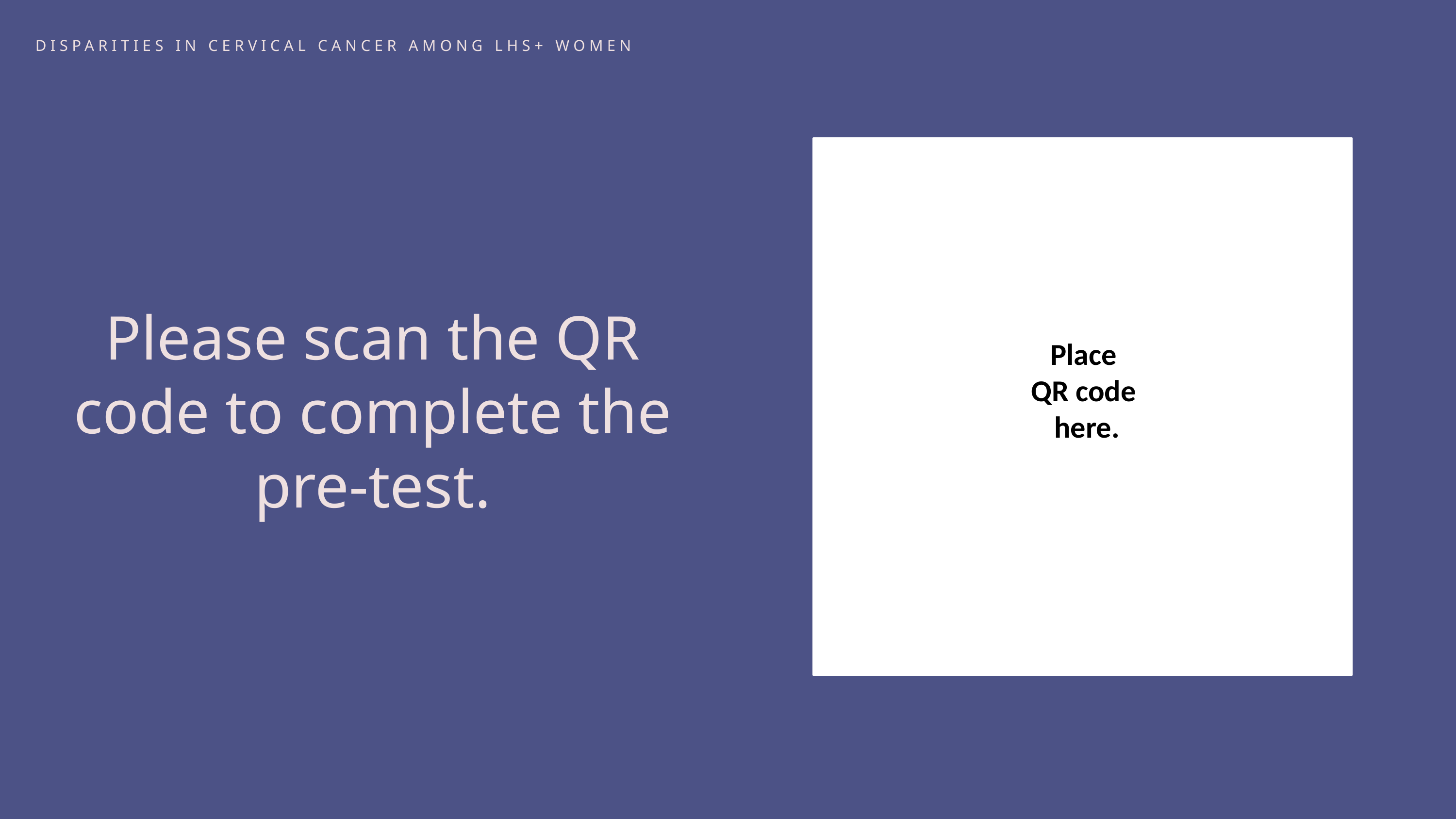

DISPARITIES IN CERVICAL CANCER AMONG LHS+ WOMEN​
Please scan the QR code to complete the pre-test.
Place
QR code
here.

## Slide 5
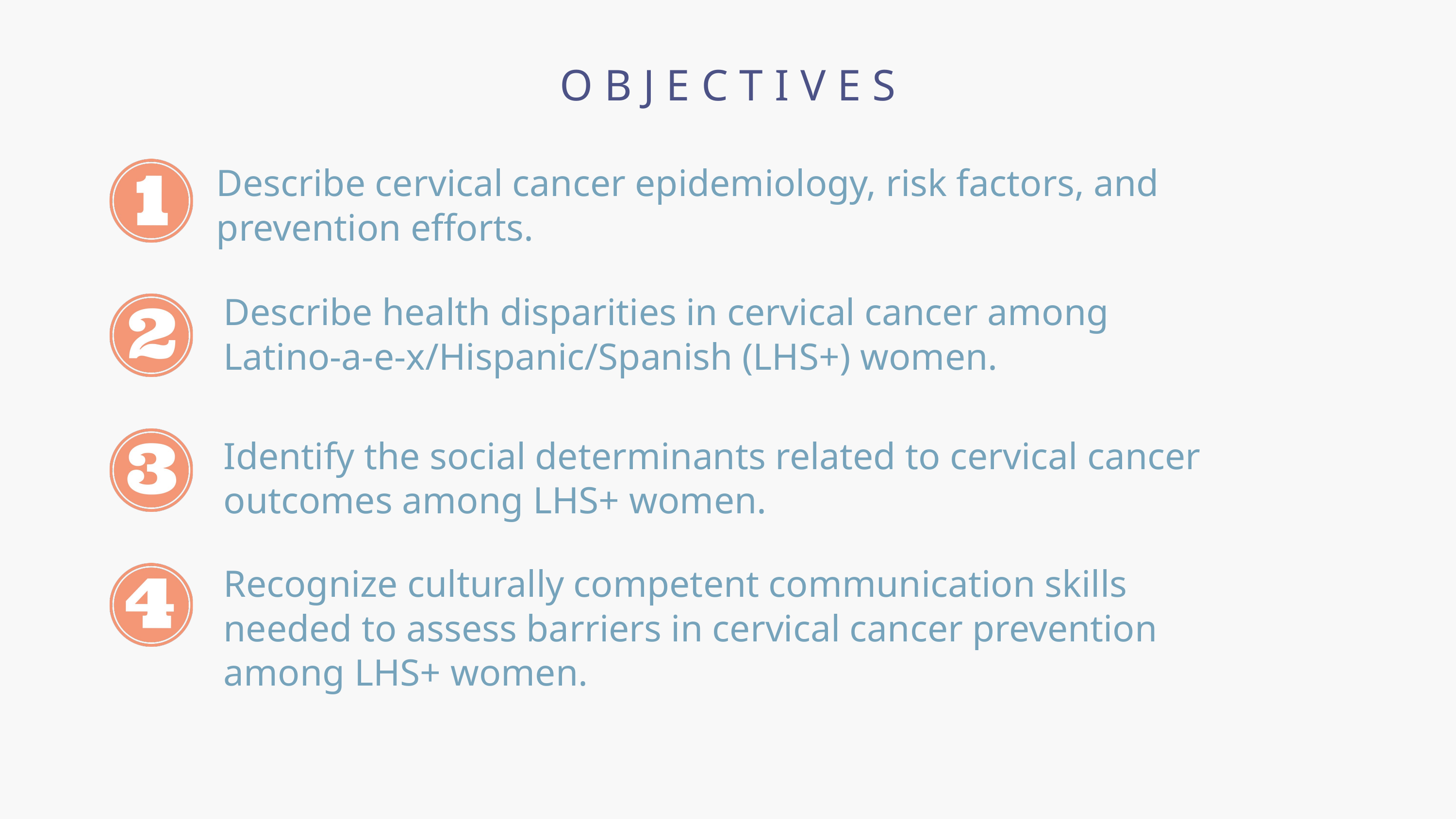

OBJECTIVES
Describe cervical cancer epidemiology, risk factors, and prevention efforts.
Describe health disparities in cervical cancer among Latino-a-e-x/Hispanic/Spanish (LHS+) women.
Identify the social determinants related to cervical cancer outcomes among LHS+ women.
Recognize culturally competent communication skills needed to assess barriers in cervical cancer prevention among LHS+ women.

## Slide 6
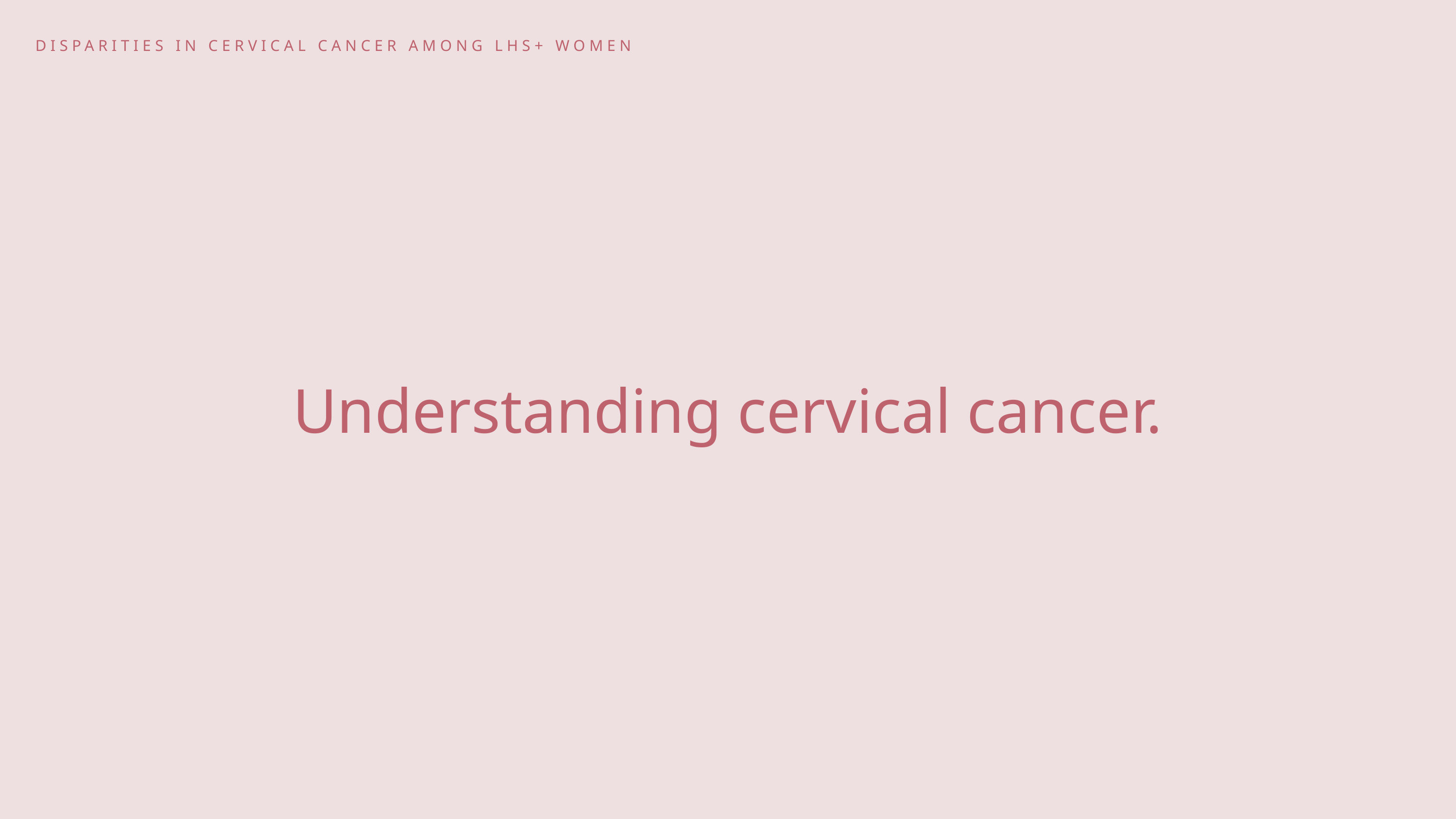

DISPARITIES IN CERVICAL CANCER AMONG LHS+ WOMEN​
Understanding cervical cancer.

## Slide 7
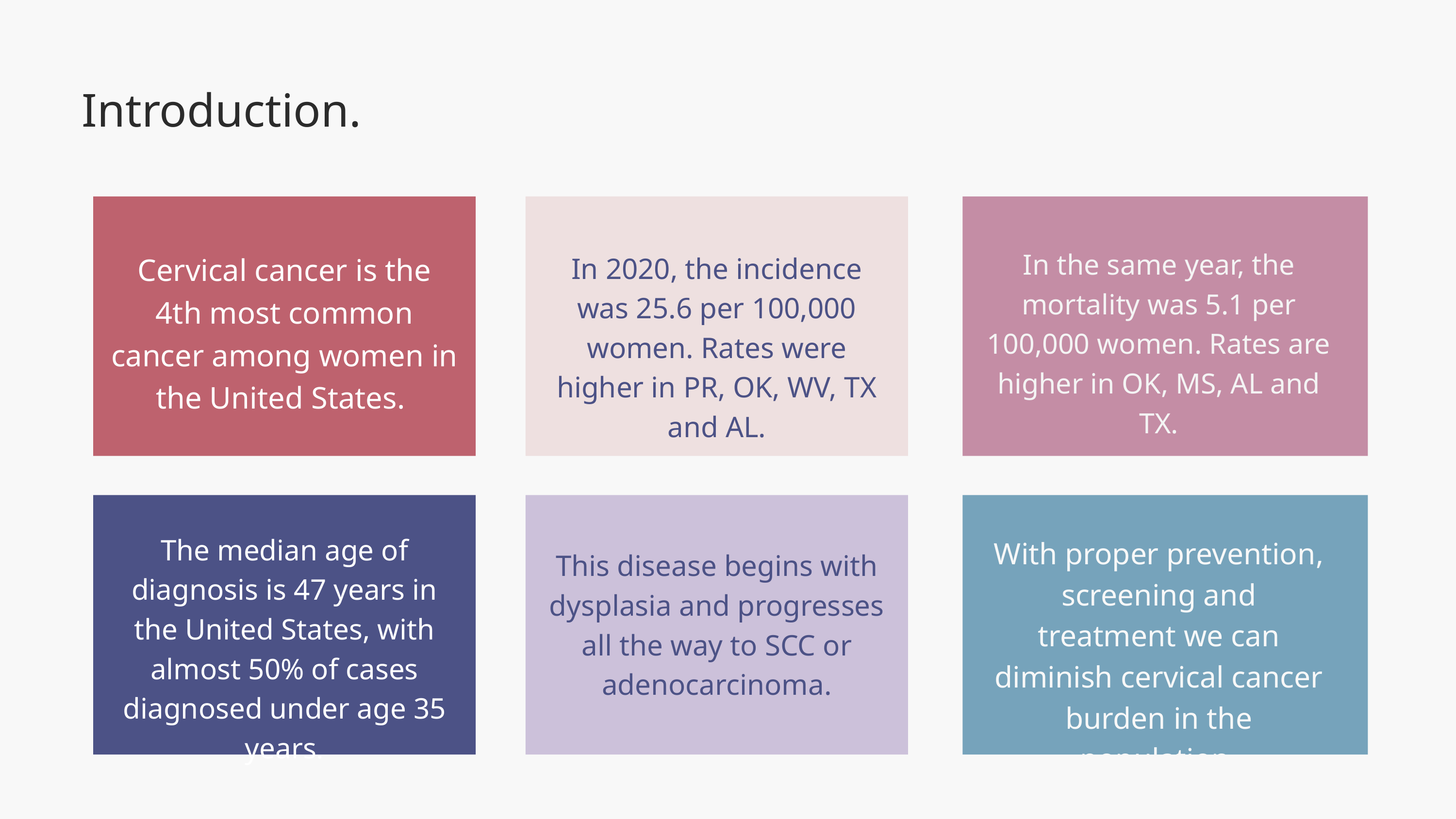

Introduction.
In the same year, the mortality was 5.1 per 100,000 women. Rates are higher in OK, MS, AL and TX.
Cervical cancer is the 4th most common cancer among women in the United States.
In 2020, the incidence was 25.6 per 100,000 women. Rates were higher in PR, OK, WV, TX and AL.
The median age of diagnosis is 47 years in the United States, with almost 50% of cases diagnosed under age 35 years.
With proper prevention, screening and treatment we can diminish cervical cancer burden in the population.​
This disease begins with dysplasia and progresses all the way to SCC or adenocarcinoma.

## Slide 8
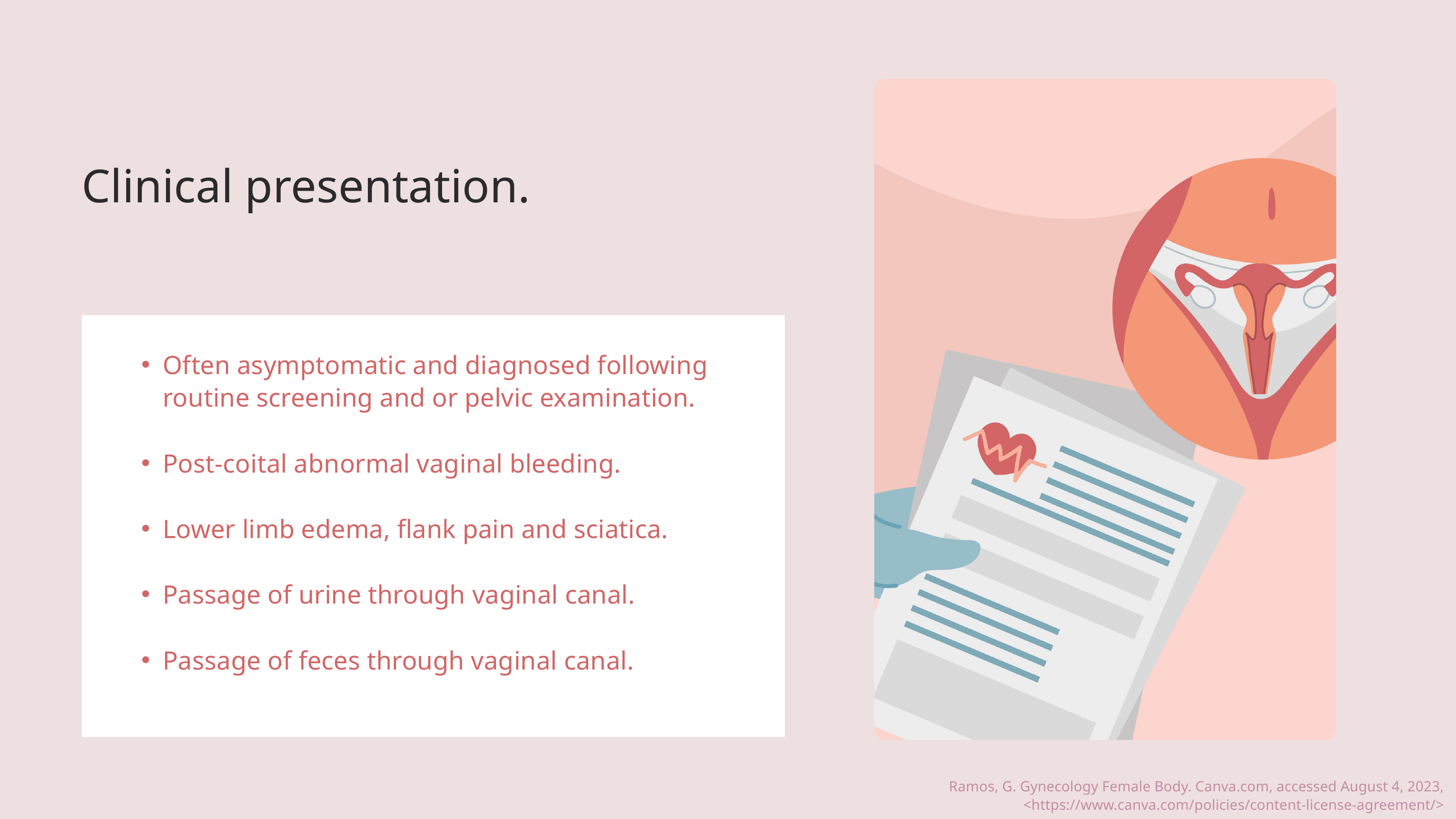

Clinical presentation.
Often asymptomatic and diagnosed following routine screening and or pelvic examination.
Post-coital abnormal vaginal bleeding.
Lower limb edema, flank pain and sciatica.
Passage of urine through vaginal canal.
Passage of feces through vaginal canal.
Ramos, G. Gynecology Female Body. Canva.com, accessed August 4, 2023, <https://www.canva.com/policies/content-license-agreement/>

## Slide 9
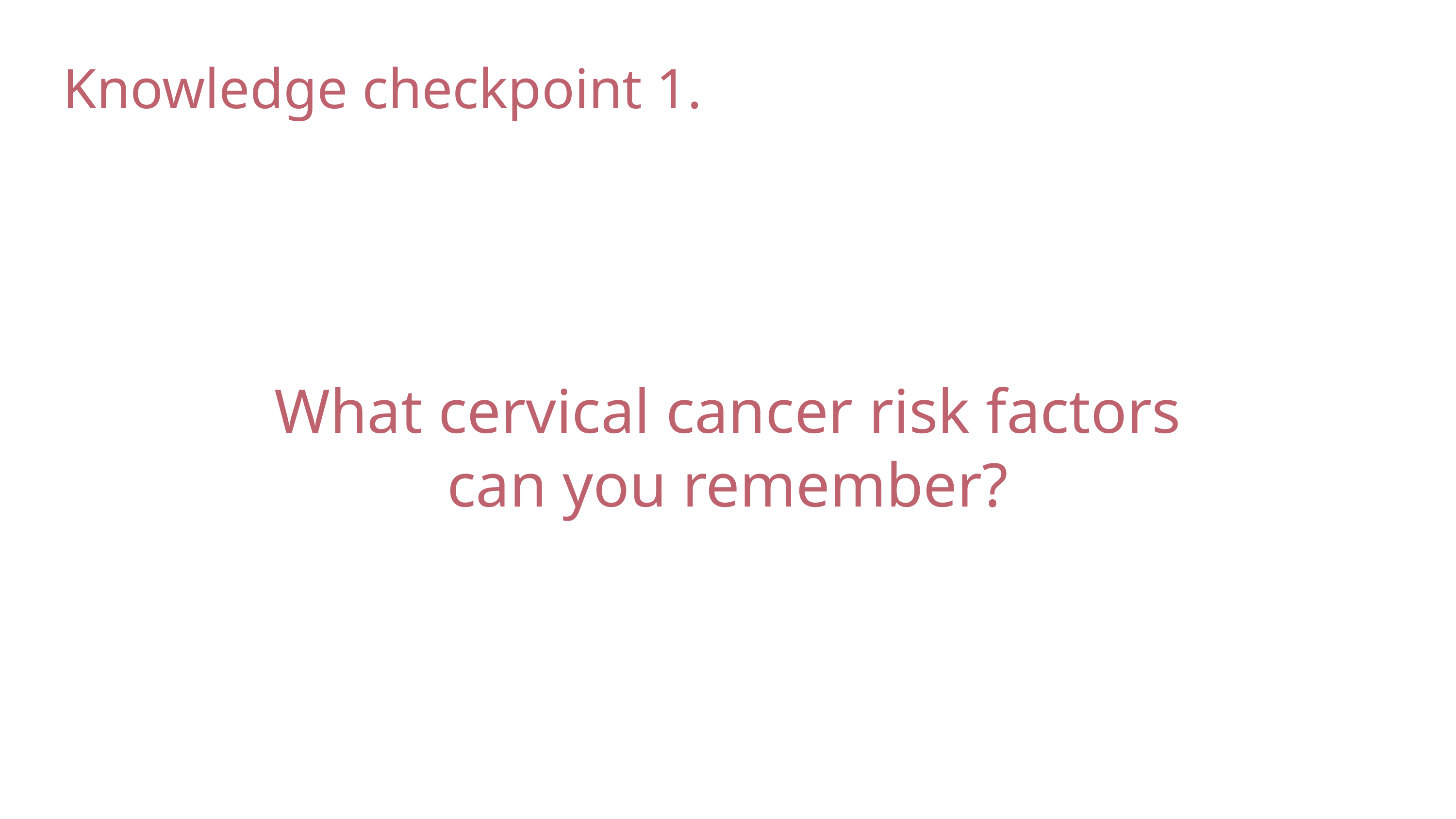

Knowledge checkpoint 1.
What cervical cancer risk factors can you remember?

## Slide 10
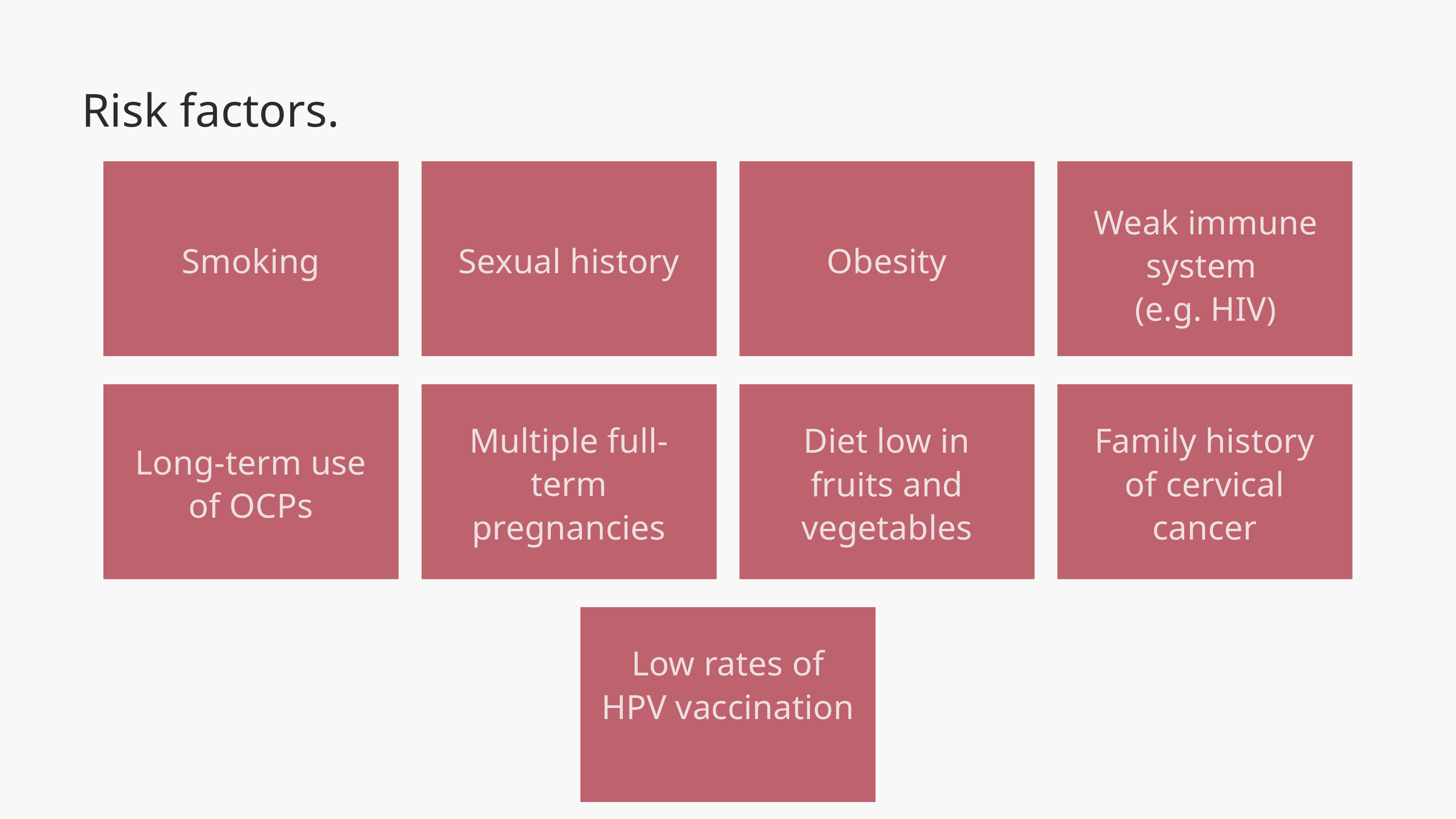

Risk factors.
Weak immune system
(e.g. HIV)
Smoking
Sexual history
Obesity
Multiple full-term pregnancies
Diet low in fruits and vegetables
Family history of cervical cancer
Long-term use of OCPs
Low rates of HPV vaccination

## Slide 11
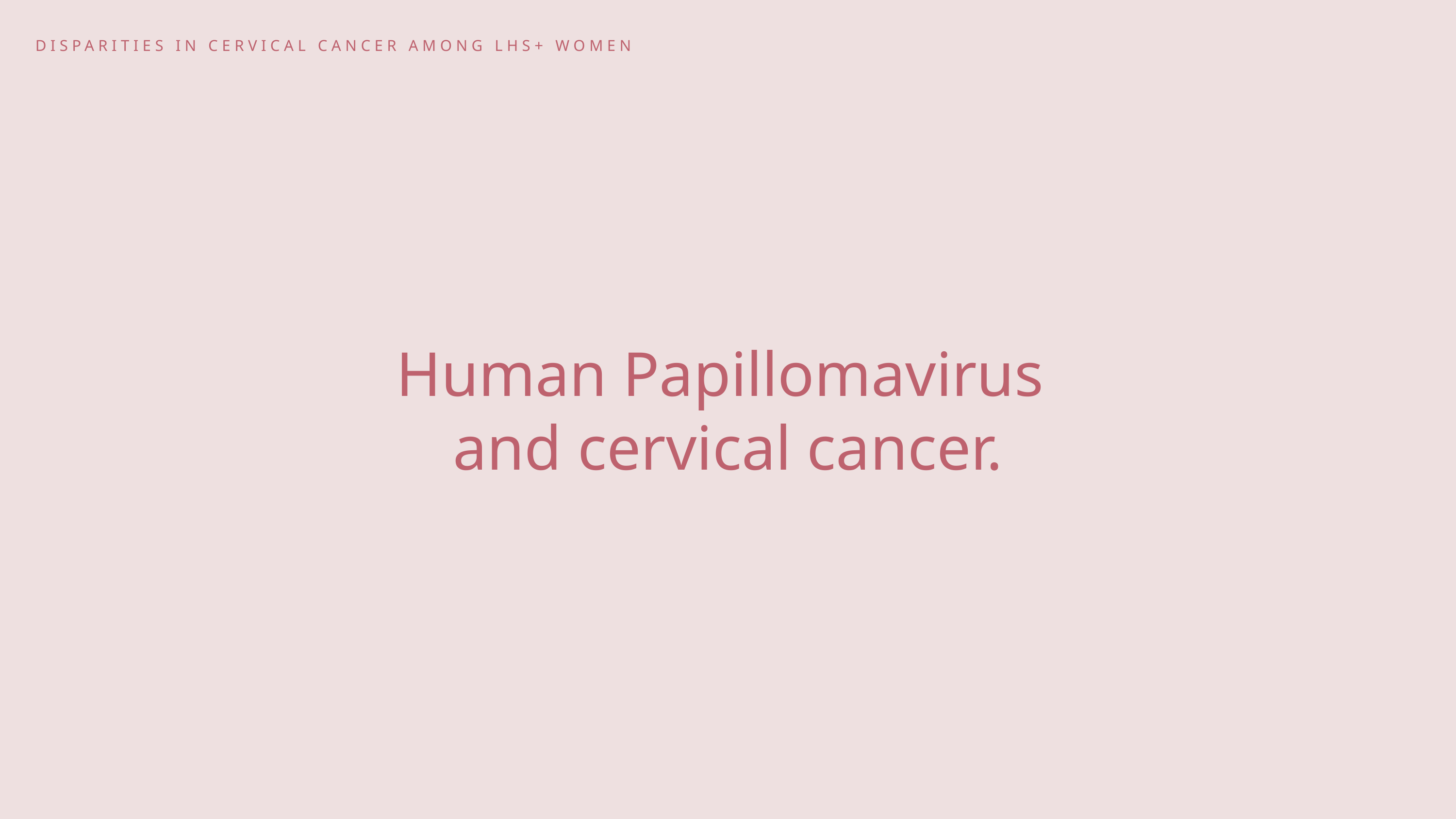

DISPARITIES IN CERVICAL CANCER AMONG LHS+ WOMEN​
Human Papillomavirus
and cervical cancer.

## Slide 12
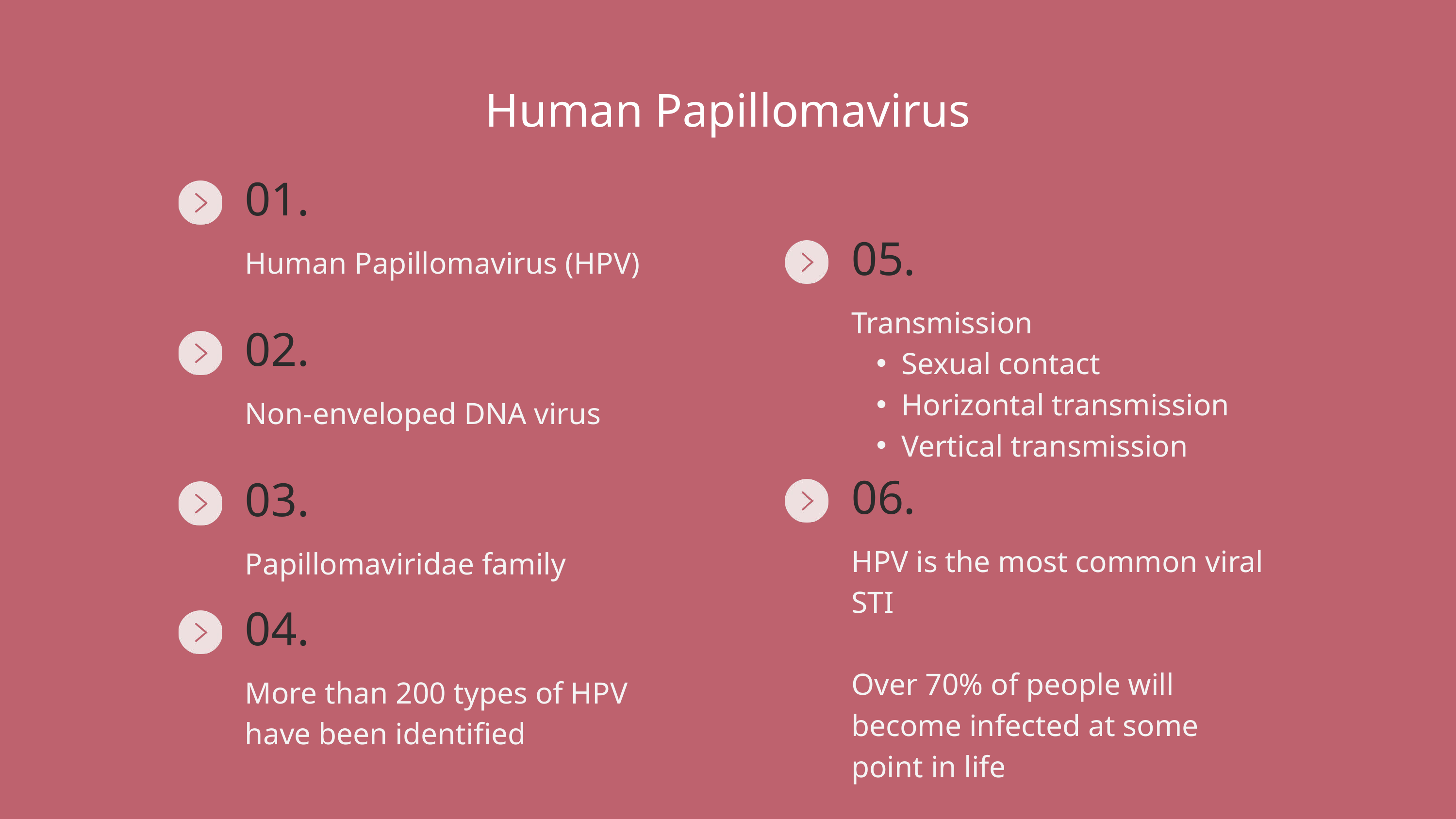

Human Papillomavirus
01.
Human Papillomavirus (HPV)
@REALLYGREATSITE
05.
Transmission
Sexual contact​
Horizontal transmission​
Vertical transmission
@REALLYGREATSITE
02.
Non-enveloped DNA virus
@REALLYGREATSITE
06.
HPV is the most common viral STI
Over 70% of people will become infected at some point in life
@REALLYGREATSITE
03.
Papillomaviridae family
@REALLYGREATSITE
04.
More than 200 types of HPV have been identified
@REALLYGREATSITE

## Slide 13
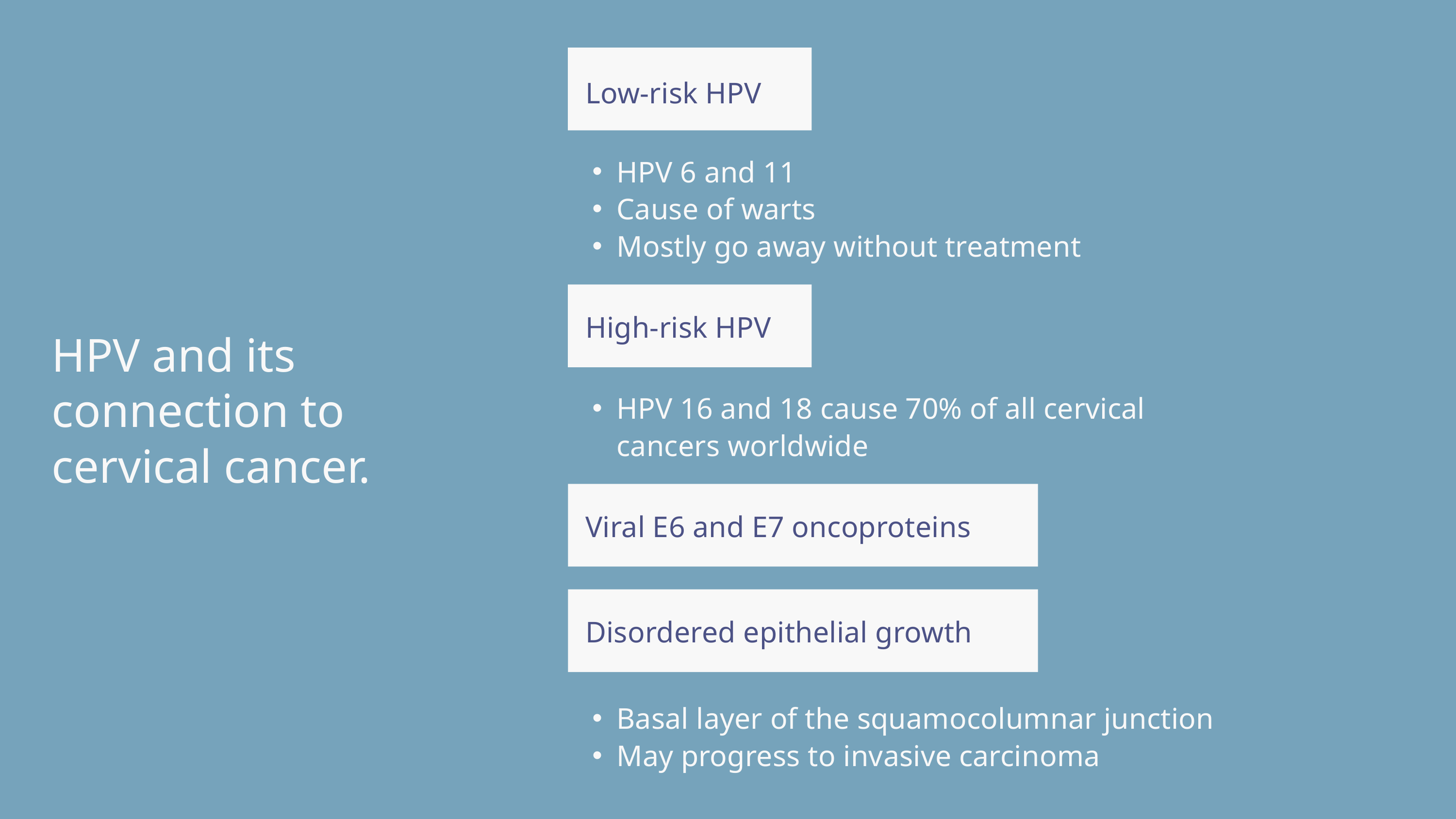

Low-risk HPV
HPV 6 and 11
Cause of warts
Mostly go away without treatment
High-risk HPV
HPV and its connection to cervical cancer.
HPV 16 and 18 cause 70% of all cervical cancers worldwide
Viral E6 and E7 oncoproteins
Disordered epithelial growth
Basal layer of the squamocolumnar junction ​
May progress to invasive carcinoma

## Slide 14
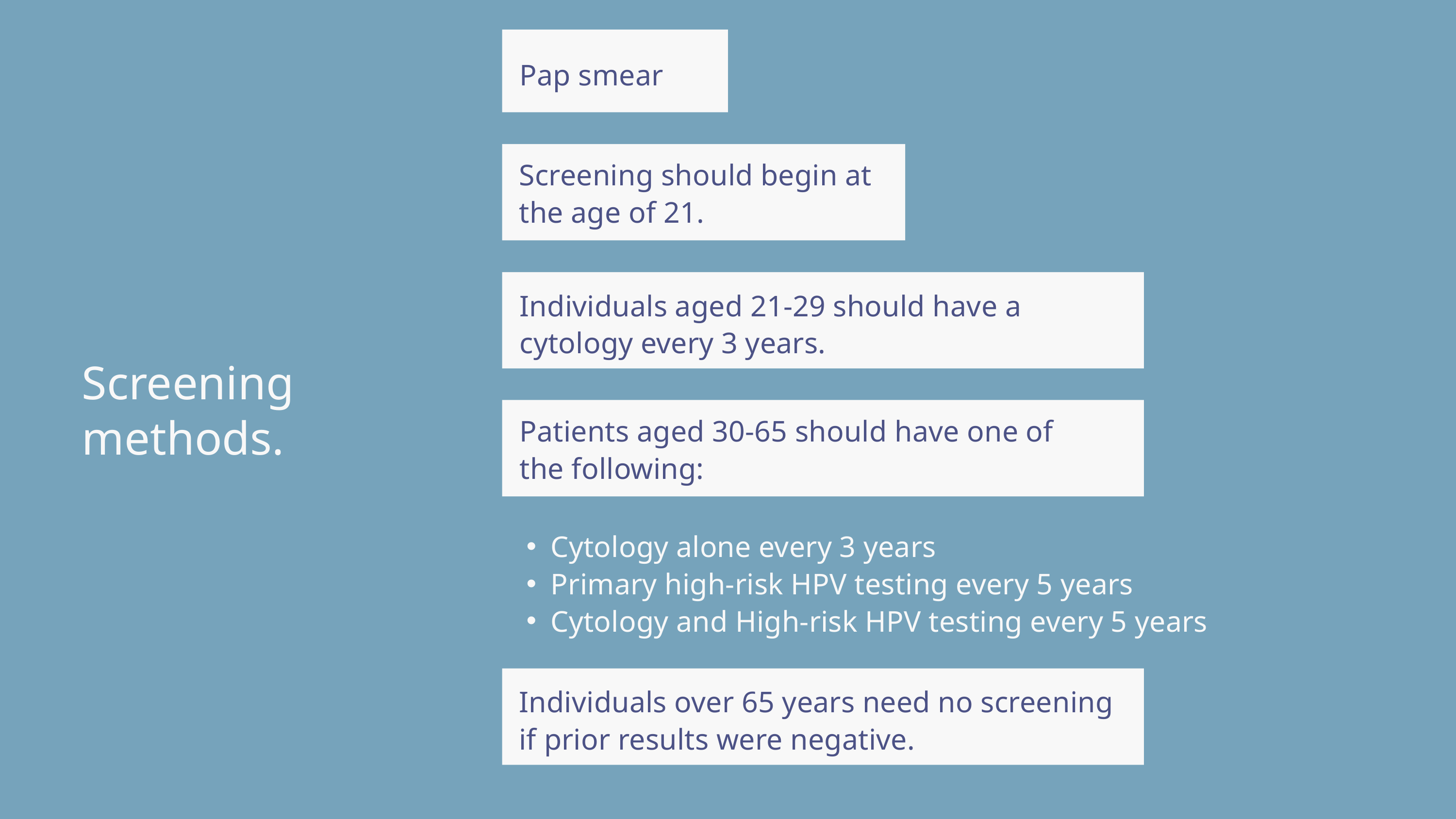

Pap smear
Screening should begin at the age of 21.
Individuals aged 21-29 should have a cytology every 3 years.
Screening methods.
Patients aged 30-65 should have one of the following:
Cytology alone every 3 years​
Primary high-risk HPV testing every 5 years​
Cytology and High-risk HPV testing every 5 years
Individuals over 65 years need no screening if prior results were negative.

## Slide 15
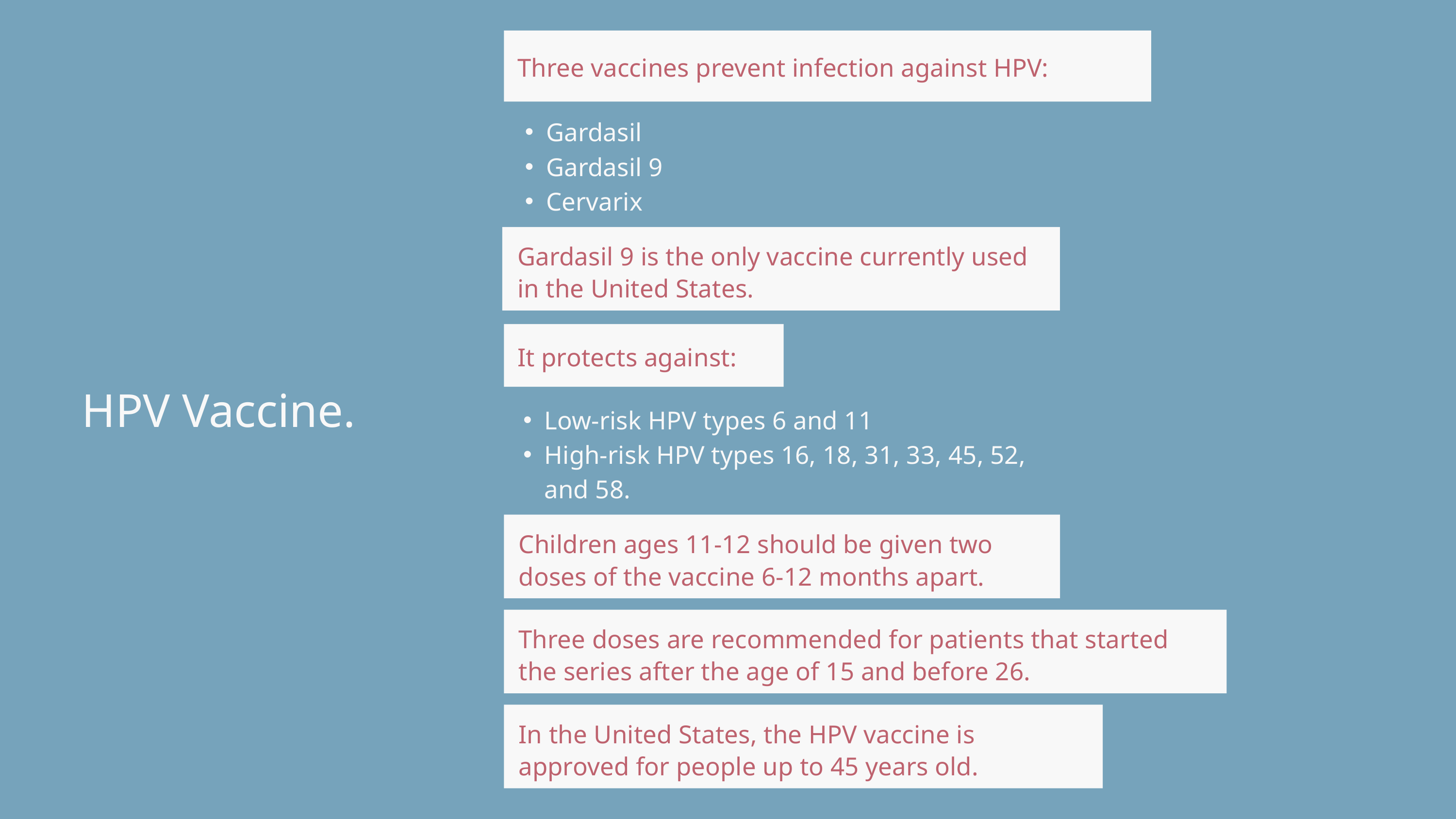

Three vaccines prevent infection against HPV:
Gardasil
Gardasil 9
Cervarix
Gardasil 9 is the only vaccine currently used in the United States.
It protects against:
HPV Vaccine.
Low-risk HPV types 6 and 11​
High-risk HPV types 16, 18, 31, 33, 45, 52, and 58.
Children ages 11-12 should be given two doses of the vaccine 6-12 months apart.
Three doses are recommended for patients that started the series after the age of 15 and before 26.
In the United States, the HPV vaccine is approved for people up to 45 years old.

## Slide 16
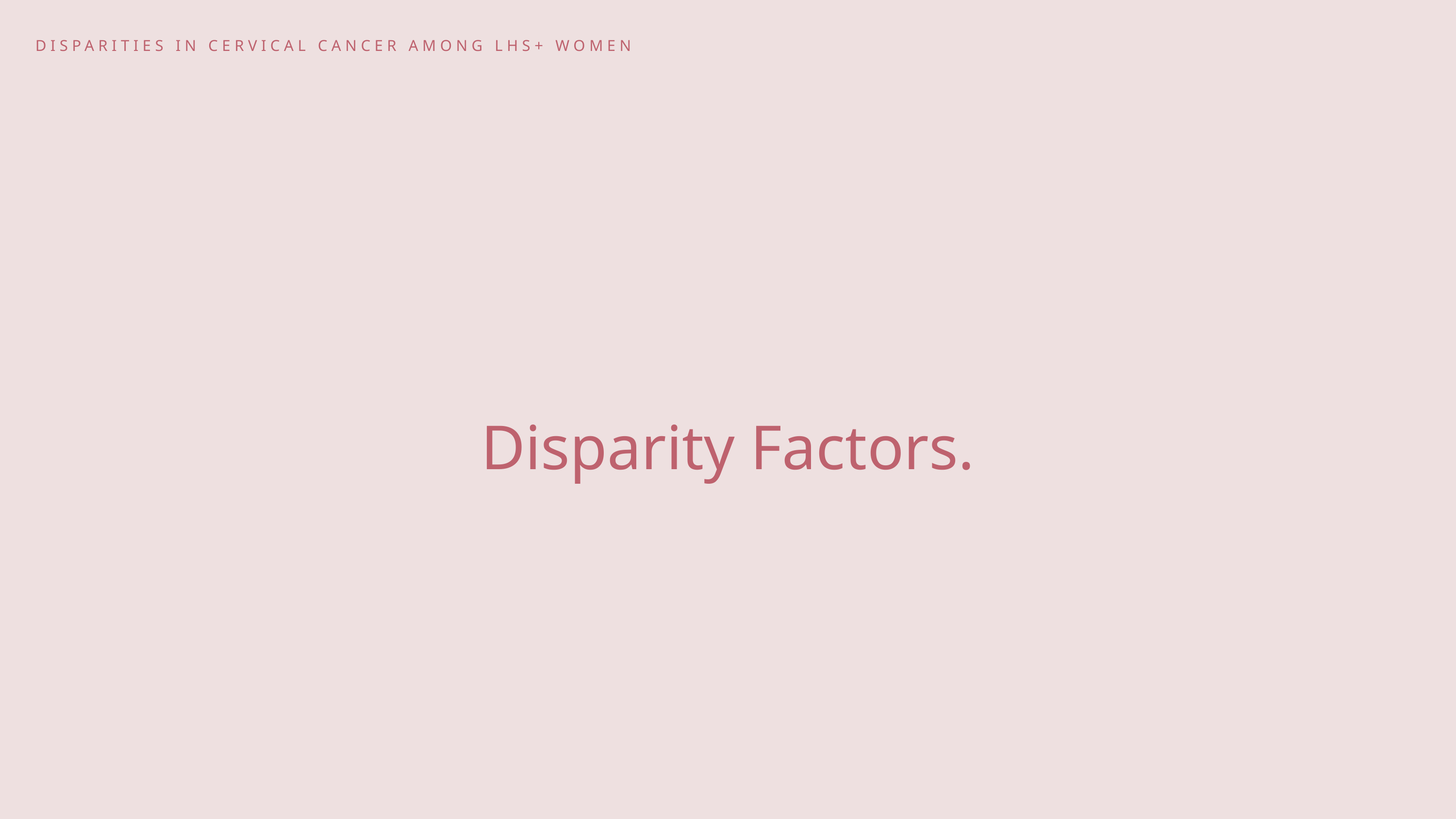

DISPARITIES IN CERVICAL CANCER AMONG LHS+ WOMEN​
Disparity Factors.

## Slide 17
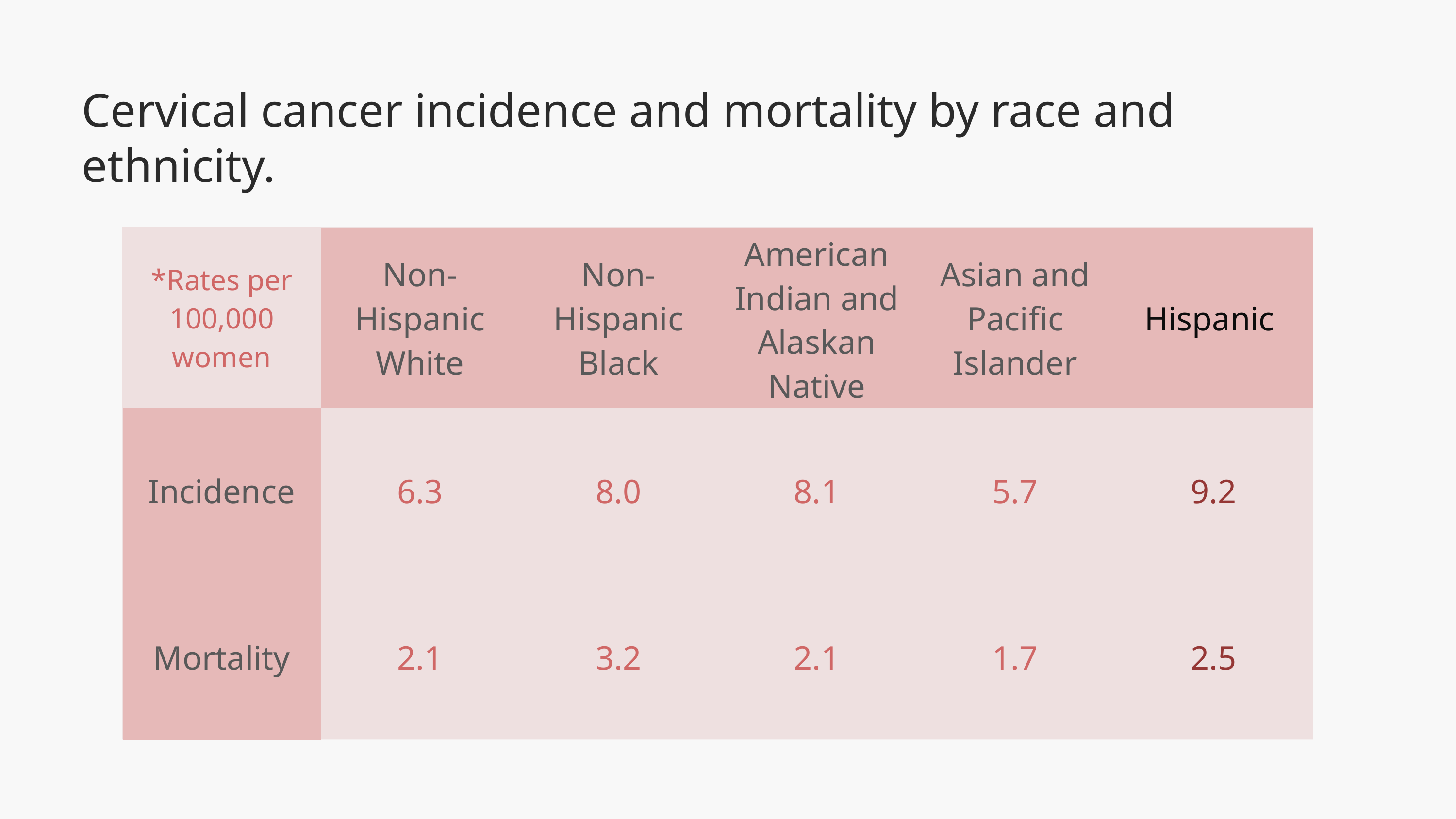

Cervical cancer incidence and mortality by race and ethnicity.
| \*Rates per 100,000 women | Non-Hispanic White | Non-Hispanic Black | American Indian and Alaskan Native | Asian and Pacific Islander | Hispanic |
| --- | --- | --- | --- | --- | --- |
| Incidence | 6.3 | 8.0 | 8.1 | 5.7 | 9.2 |
| Mortality | 2.1 | 3.2 | 2.1 | 1.7 | 2.5 |

## Slide 18
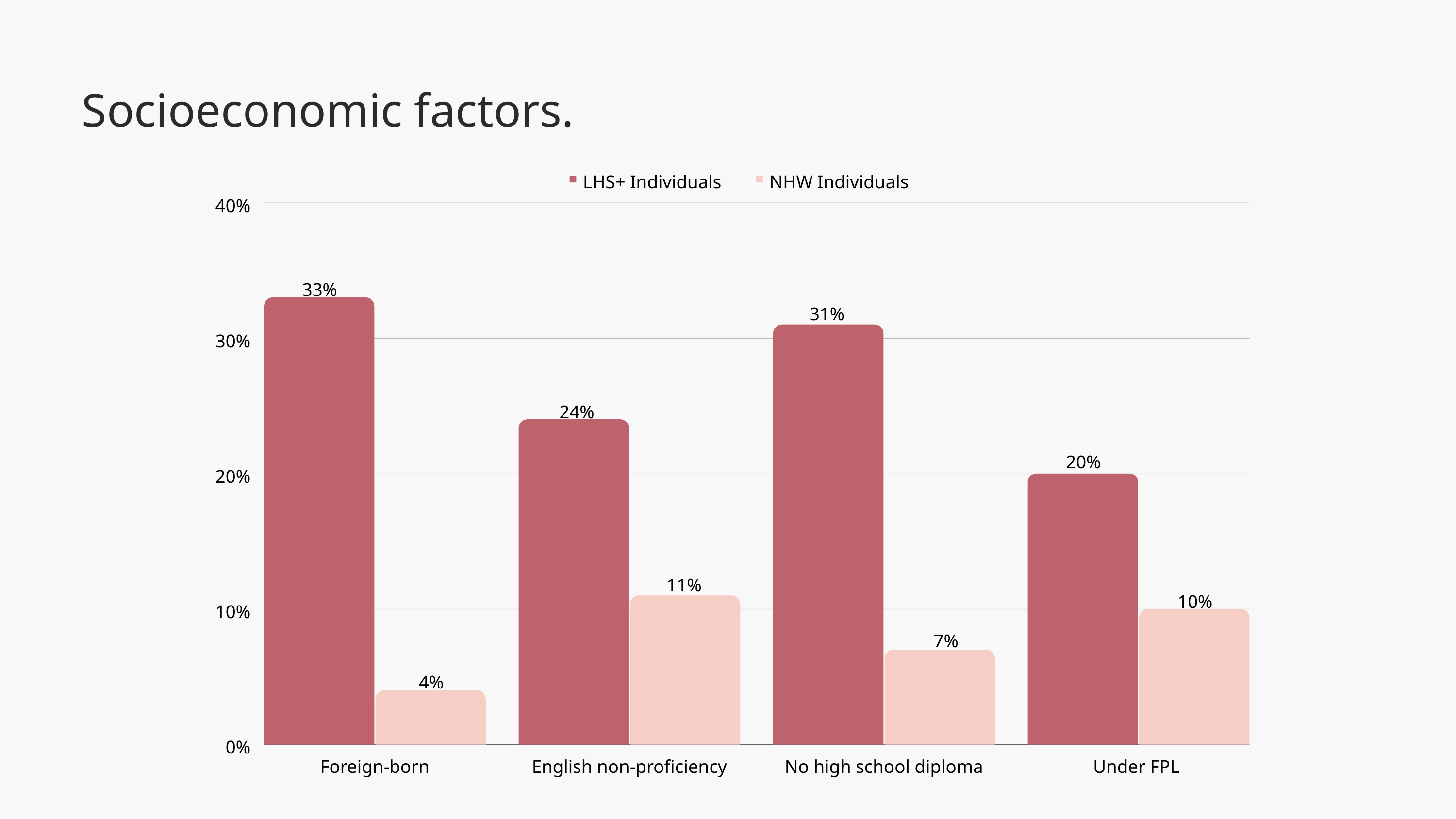

Socioeconomic factors.
LHS+ Individuals
NHW Individuals
40%
30%
20%
10%
0%
Foreign-born
English non-proficiency
No high school diploma
Under FPL
33%
31%
24%
20%
11%
10%
7%
4%

## Slide 19
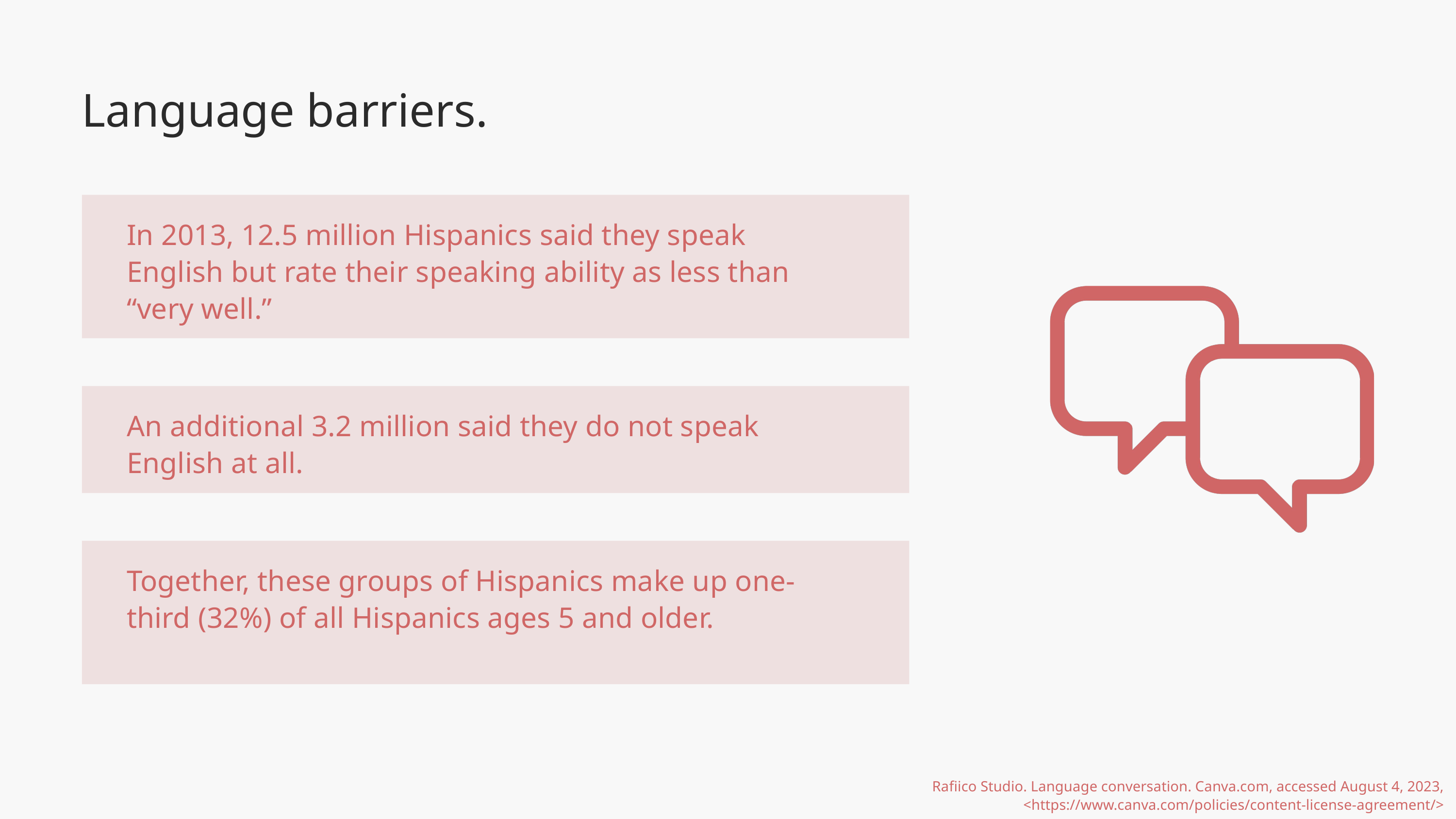

Language barriers.
In 2013, 12.5 million Hispanics said they speak English but rate their speaking ability as less than “very well.”
An additional 3.2 million said they do not speak English at all.
Together, these groups of Hispanics make up one-third (32%) of all Hispanics ages 5 and older.
Rafiico Studio. Language conversation. Canva.com, accessed August 4, 2023, <https://www.canva.com/policies/content-license-agreement/>

## Slide 20
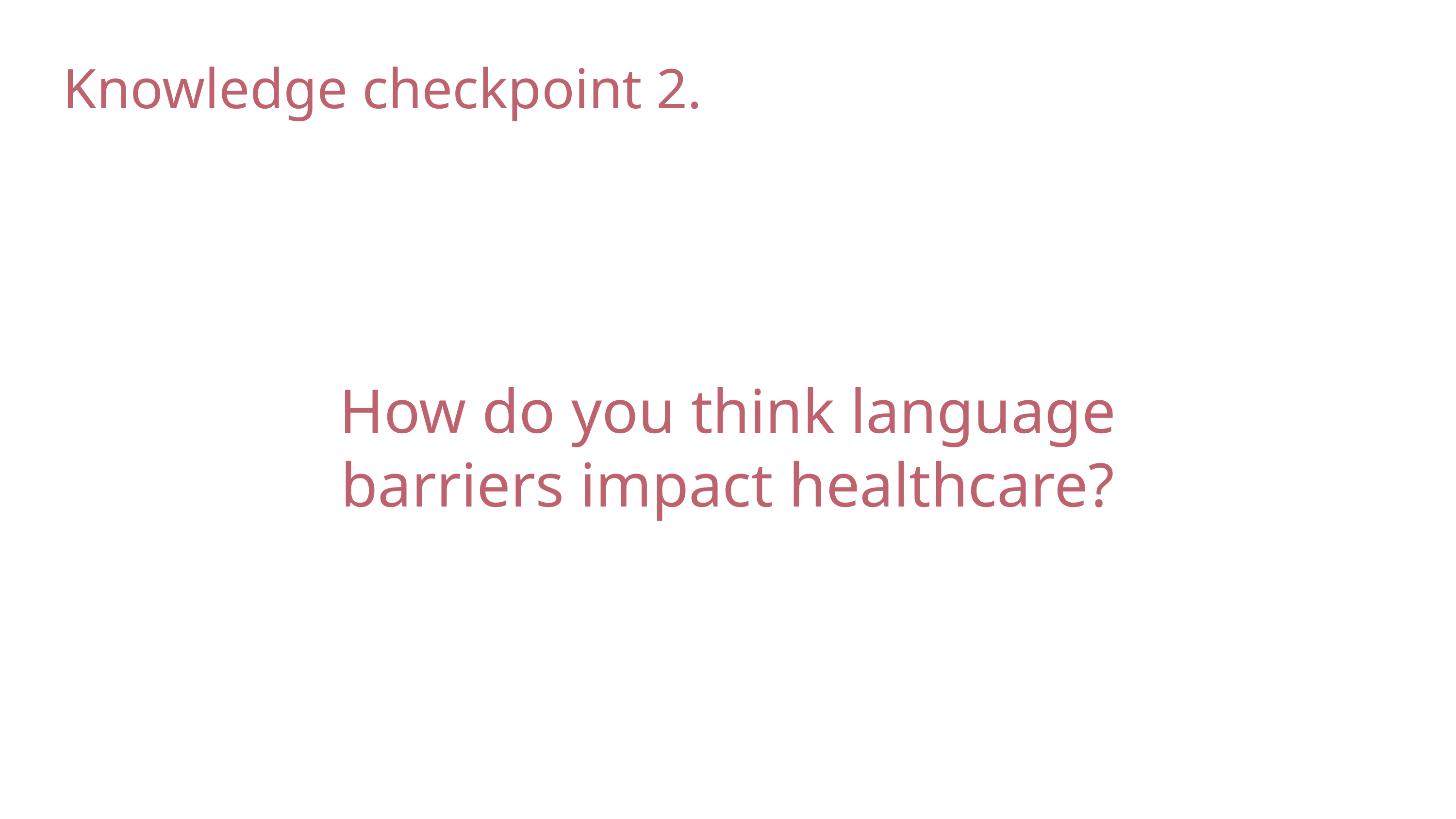

Knowledge checkpoint 2.
How do you think language barriers impact healthcare?

## Slide 21
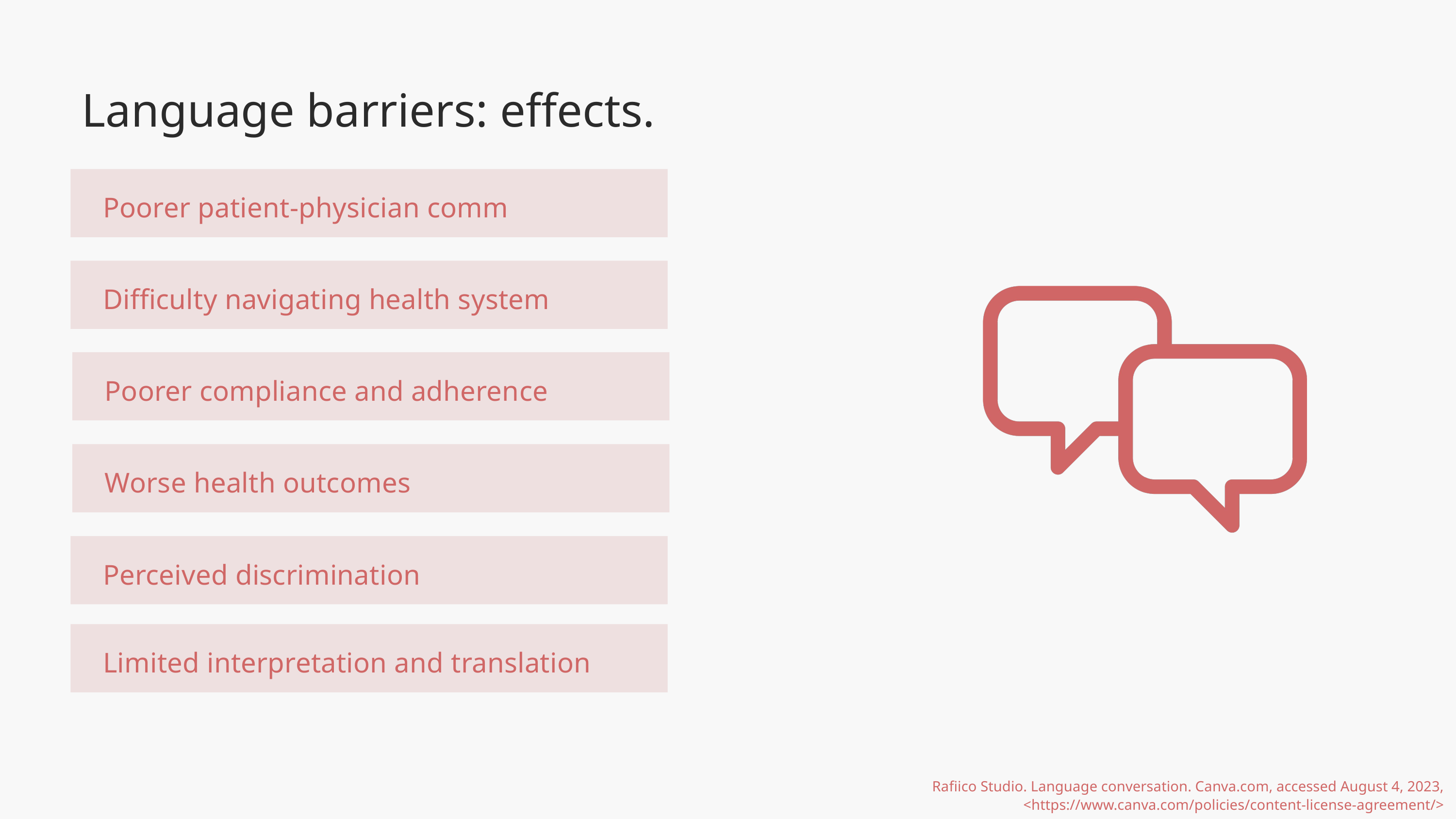

Language barriers: effects.
Poorer patient-physician comm
Difficulty navigating health system
Poorer compliance and adherence
Worse health outcomes
Perceived discrimination
Limited interpretation and translation
Rafiico Studio. Language conversation. Canva.com, accessed August 4, 2023, <https://www.canva.com/policies/content-license-agreement/>

## Slide 22
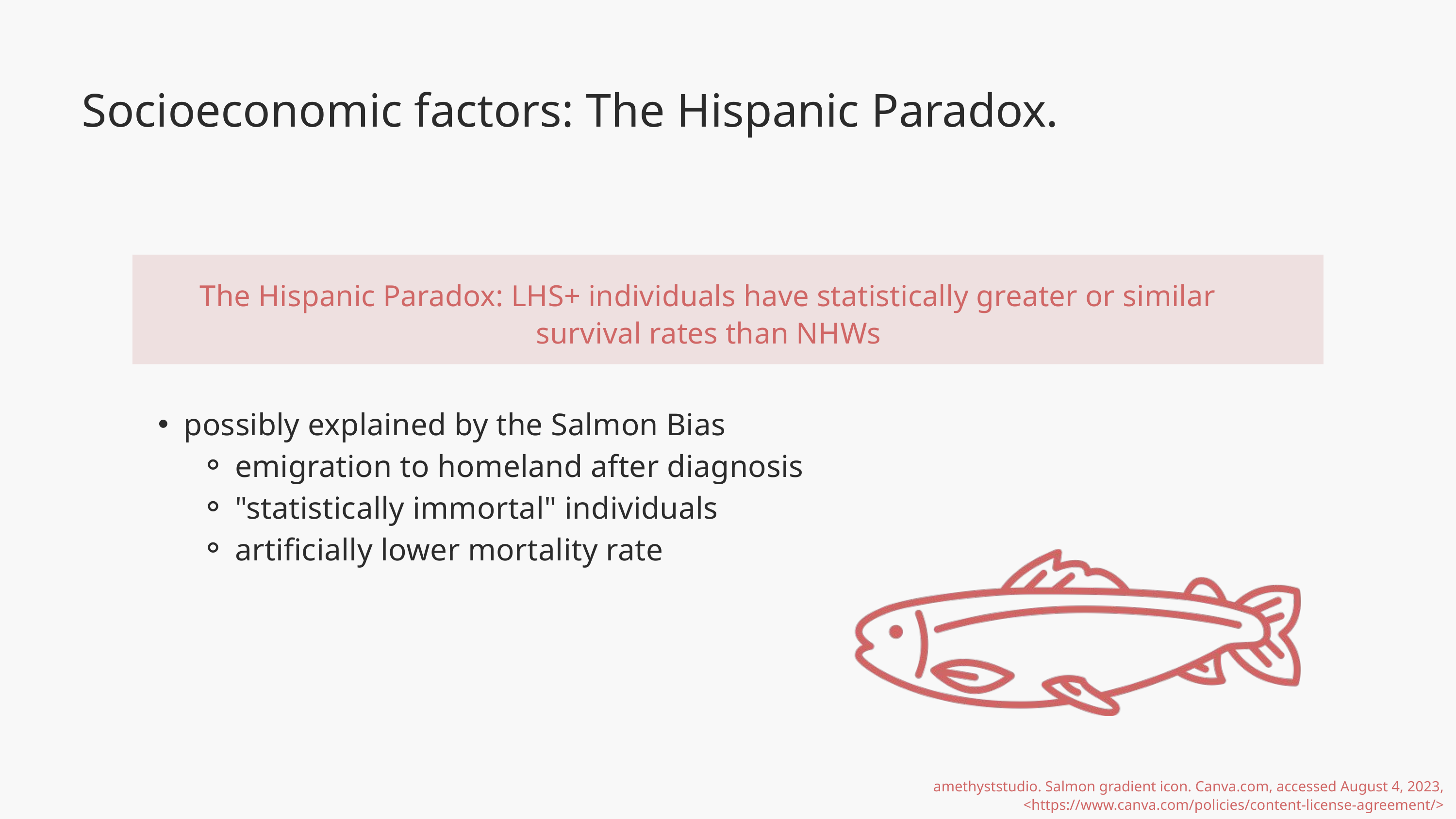

Socioeconomic factors: The Hispanic Paradox.
The Hispanic Paradox: LHS+ individuals have statistically greater or similar survival rates than NHWs
possibly explained by the Salmon Bias
emigration to homeland after diagnosis
"statistically immortal" individuals
artificially lower mortality rate
amethyststudio. Salmon gradient icon. Canva.com, accessed August 4, 2023, <https://www.canva.com/policies/content-license-agreement/>

## Slide 23
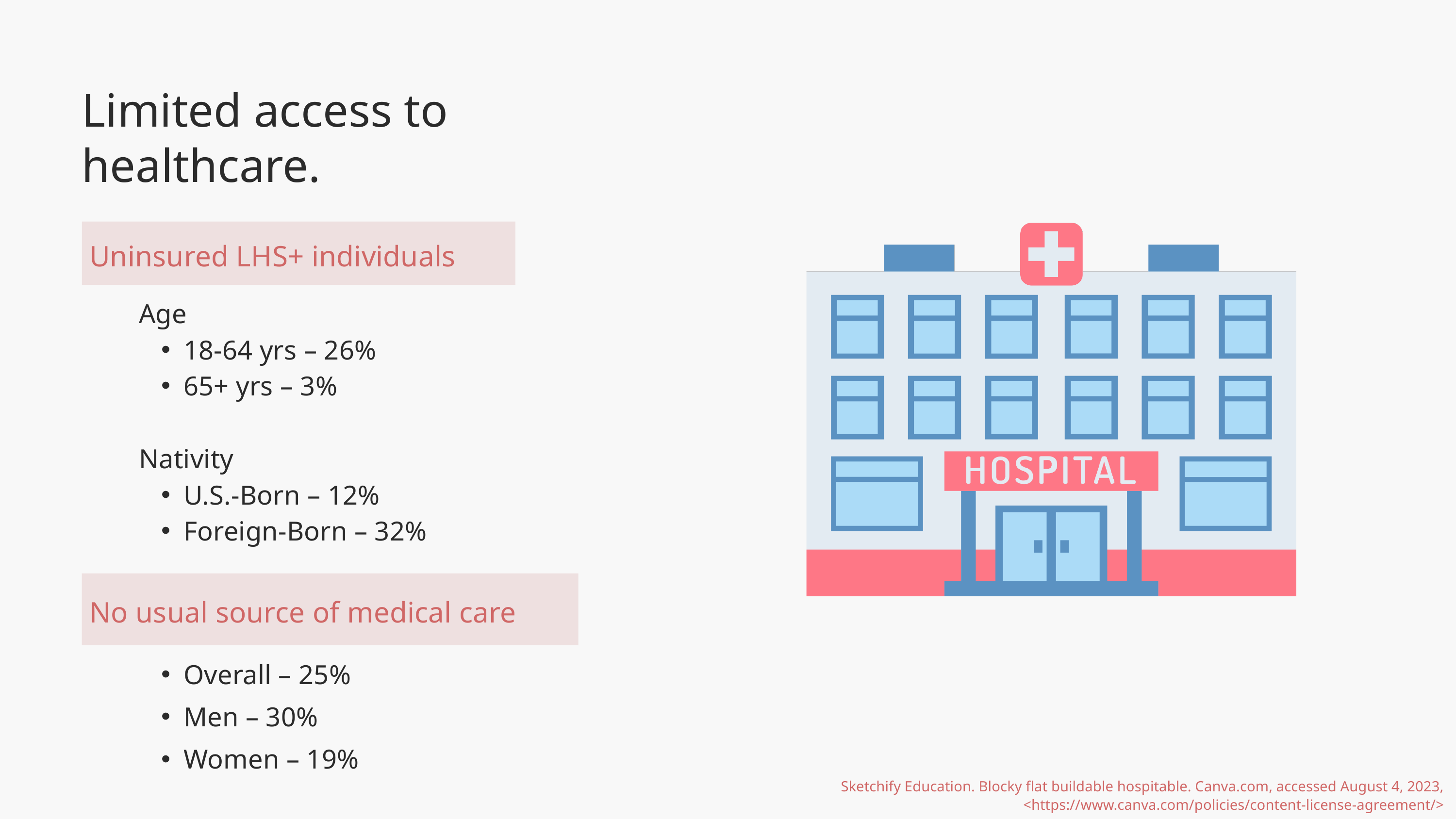

Limited access to healthcare.
Uninsured LHS+ individuals
Age​
18-64 yrs – 26%
65+ yrs – 3%
Nativity​
U.S.-Born – 12%
Foreign-Born – 32%
No usual source of medical care
Overall – 25%
Men – 30%
Women – 19%
Sketchify Education. Blocky flat buildable hospitable. Canva.com, accessed August 4, 2023, <https://www.canva.com/policies/content-license-agreement/>

## Slide 24
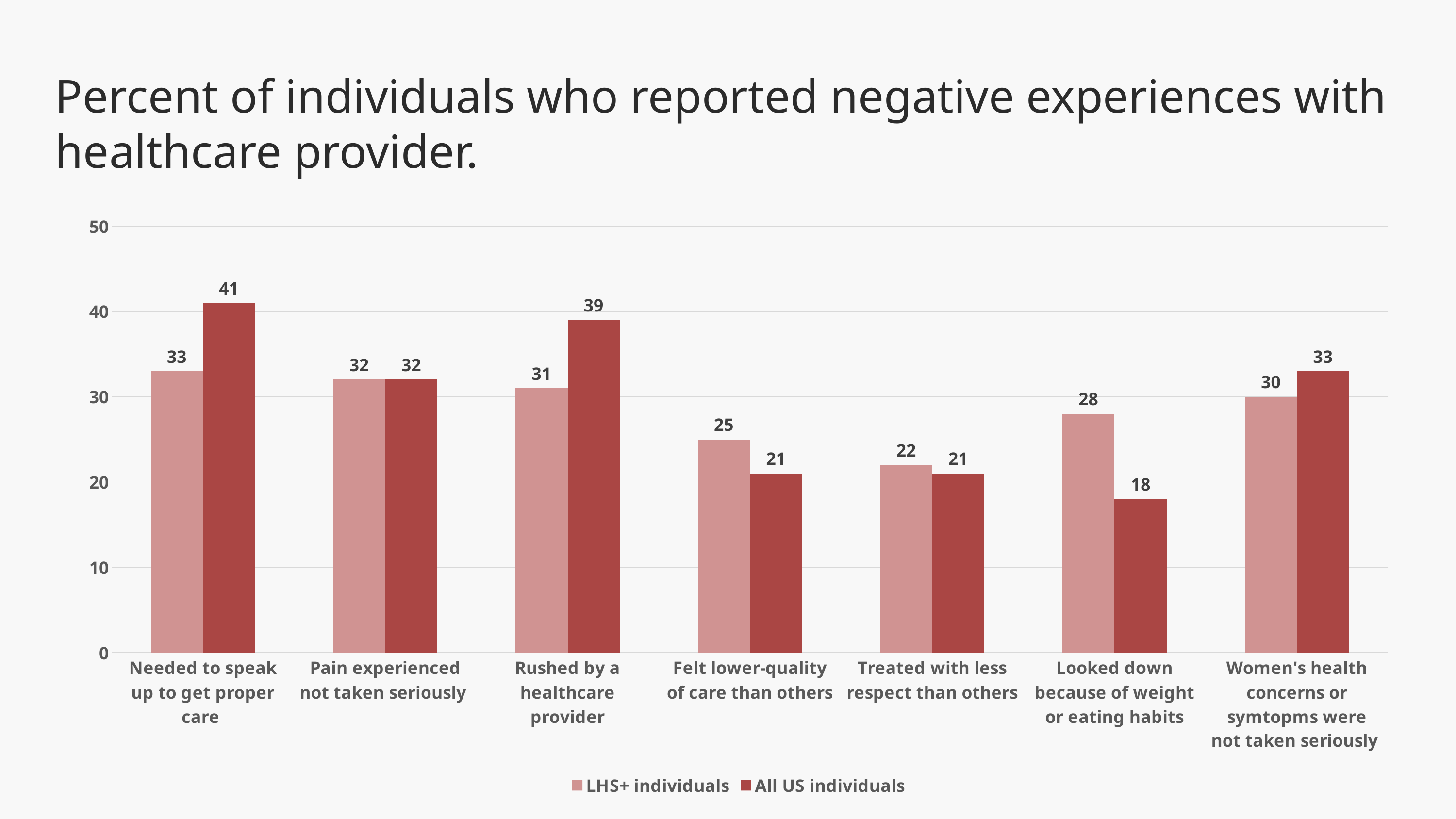

Percent of individuals who reported negative experiences with healthcare provider.
### Chart
| Category | LHS+ individuals | All US individuals |
|---|---|---|
| Needed to speak up to get proper care | 33.0 | 41.0 |
| Pain experienced not taken seriously | 32.0 | 32.0 |
| Rushed by a healthcare provider | 31.0 | 39.0 |
| Felt lower-quality of care than others | 25.0 | 21.0 |
| Treated with less respect than others | 22.0 | 21.0 |
| Looked down because of weight or eating habits | 28.0 | 18.0 |
| Women's health concerns or symtopms were not taken seriously | 30.0 | 33.0 |

## Slide 25
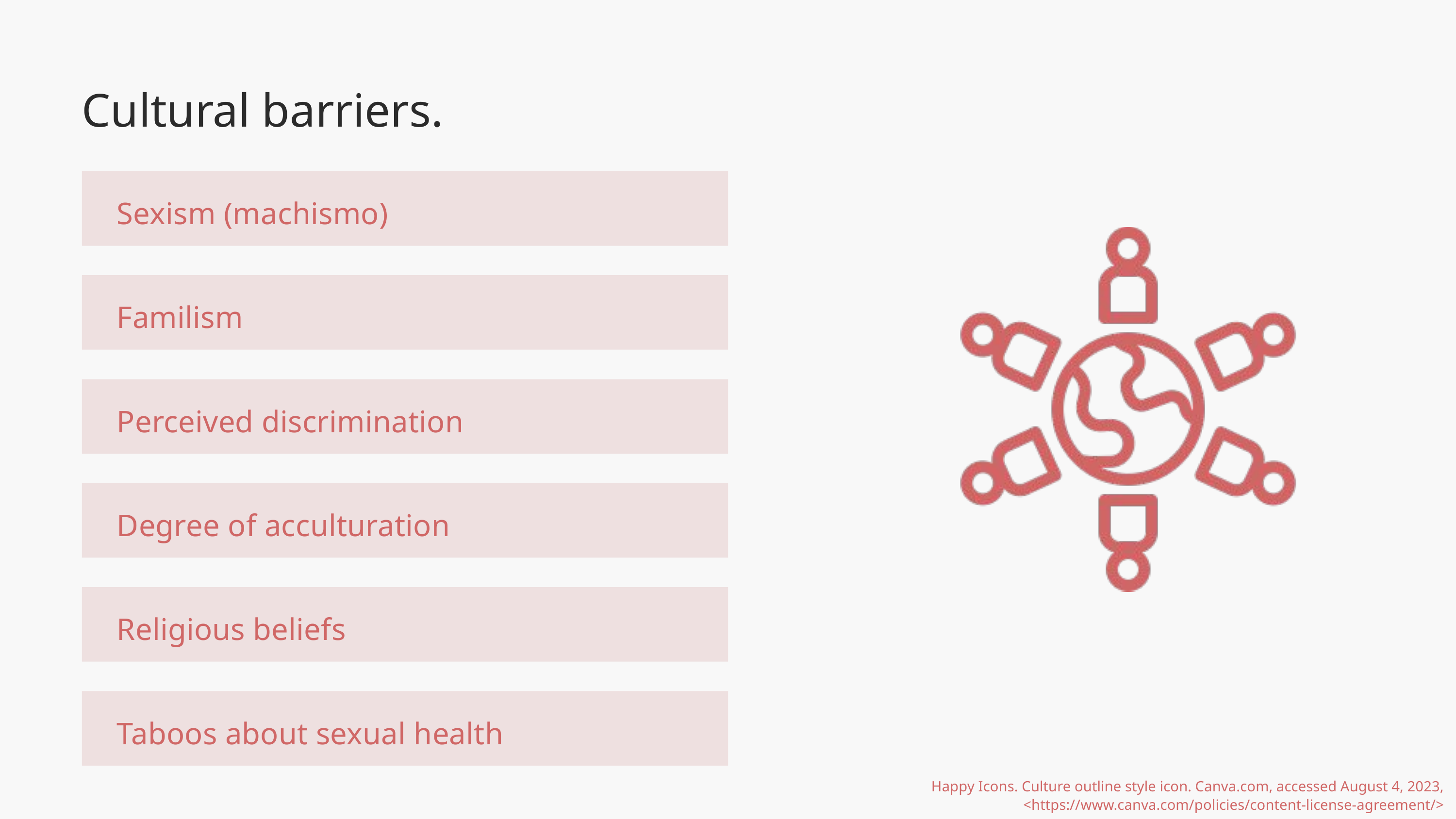

Cultural barriers.
Sexism (machismo) ​
Familism
Perceived discrimination
Degree of acculturation
Religious beliefs
Taboos about sexual health
Happy Icons. Culture outline style icon. Canva.com, accessed August 4, 2023, <https://www.canva.com/policies/content-license-agreement/>

## Slide 26
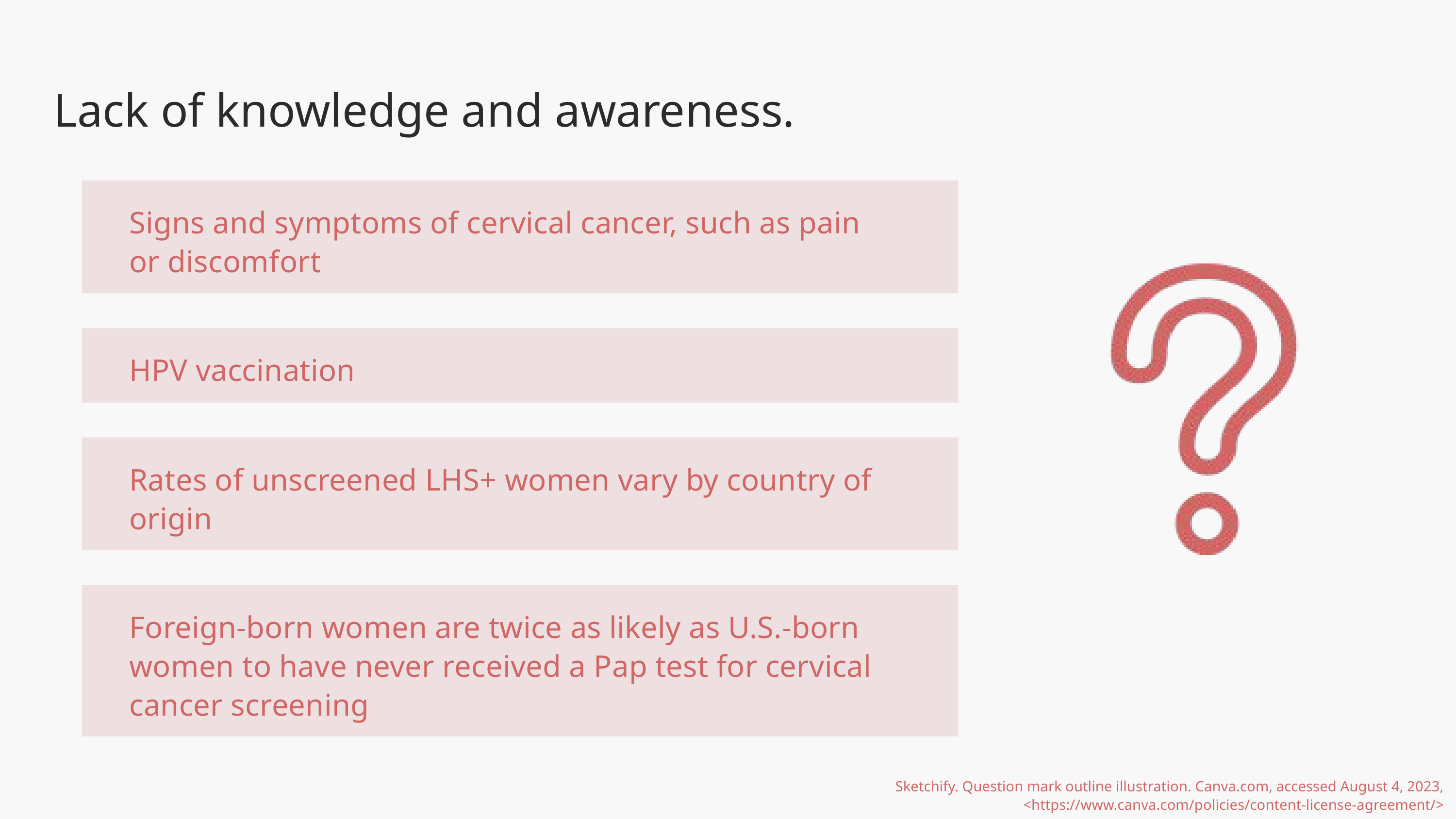

Lack of knowledge and awareness.
Signs and symptoms of cervical cancer, such as pain or discomfort
HPV vaccination
Rates of unscreened LHS+ women vary by country of origin
Foreign-born women are twice as likely as U.S.-born women to have never received a Pap test for cervical cancer screening
Sketchify. Question mark outline illustration. Canva.com, accessed August 4, 2023, <https://www.canva.com/policies/content-license-agreement/>

## Slide 27
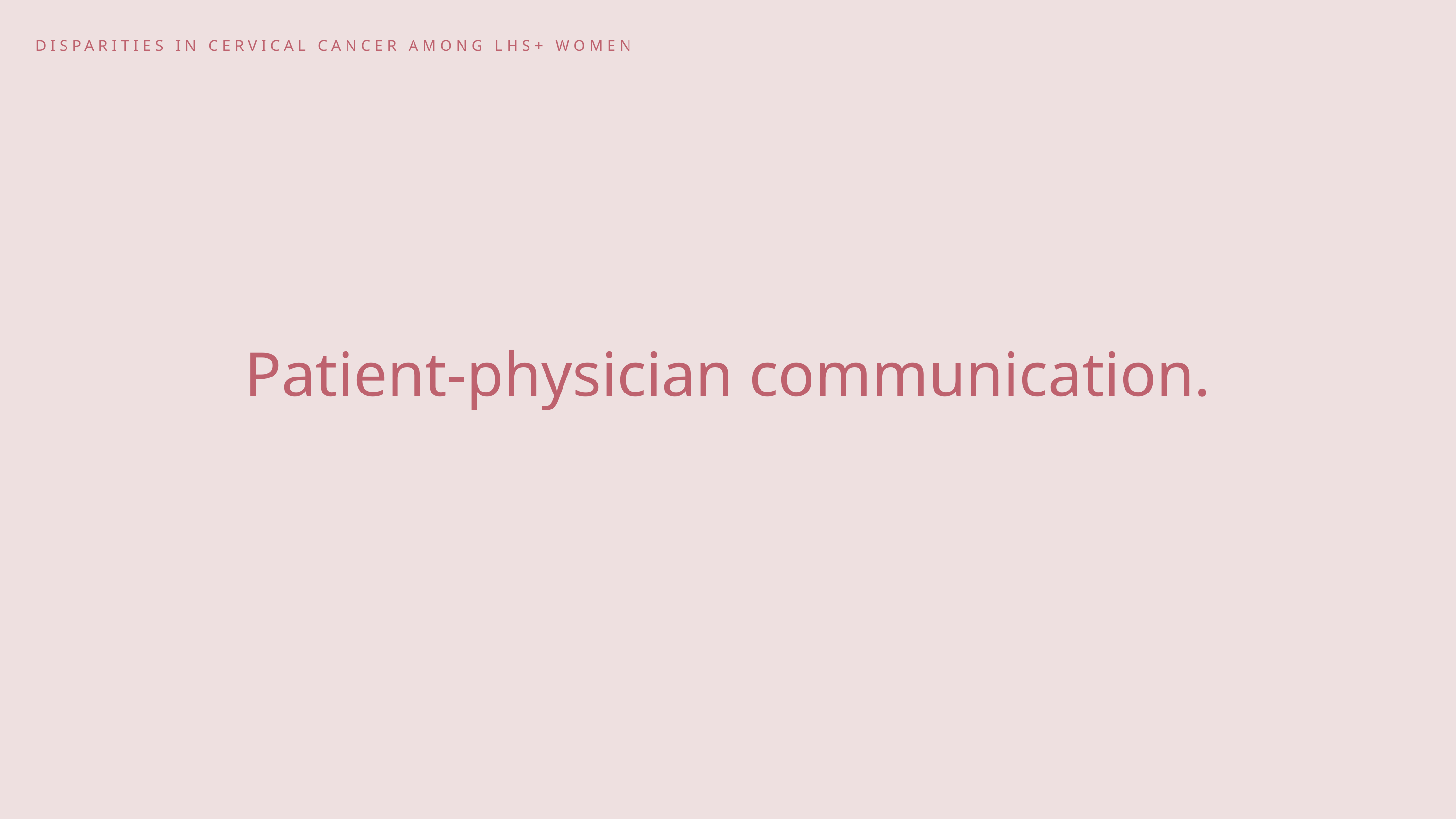

DISPARITIES IN CERVICAL CANCER AMONG LHS+ WOMEN​
Patient-physician communication.

## Slide 28
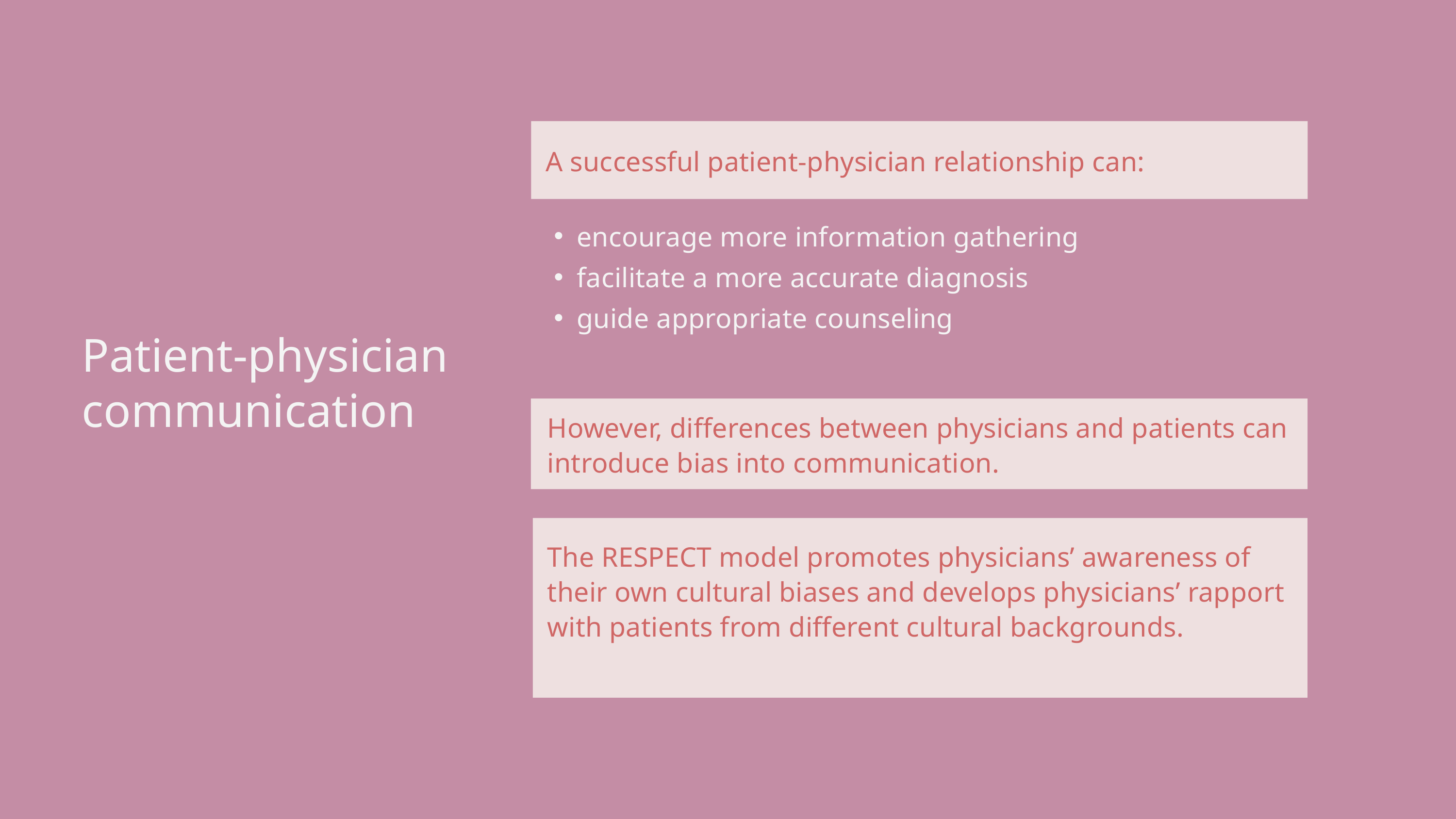

A successful patient-physician relationship can:
encourage more information gathering​
facilitate a more accurate diagnosis
guide appropriate counseling
Patient-physician communication
However, differences between physicians and patients can introduce bias into communication.
The RESPECT model promotes physicians’ awareness of their own cultural biases and develops physicians’ rapport with patients from different cultural backgrounds.

## Slide 29
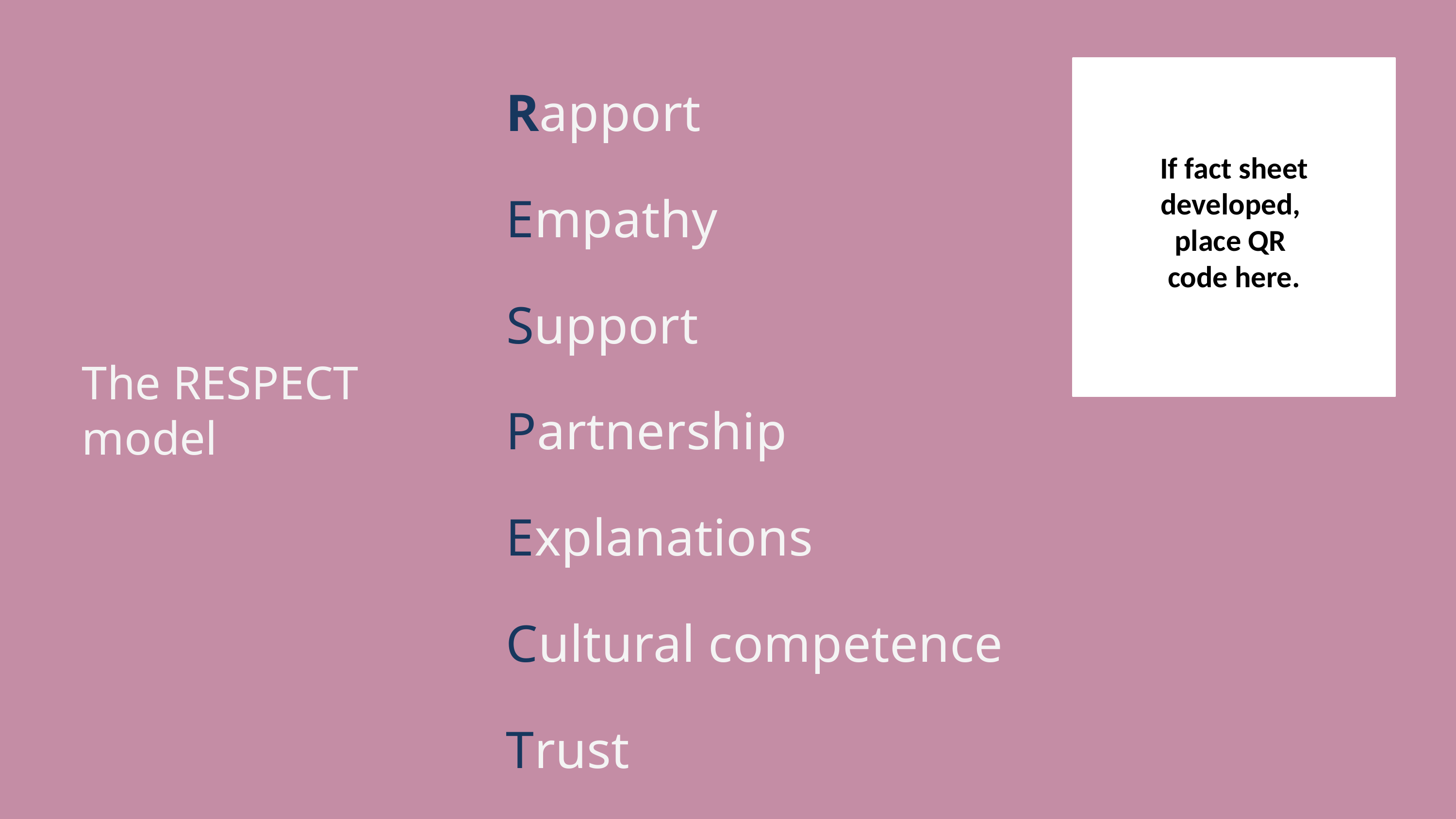

Rapport
Empathy
​Support
Partnership
Explanations
Cultural competence
Trust
If fact sheet developed,
place QR
code here.
The RESPECT model

## Slide 30
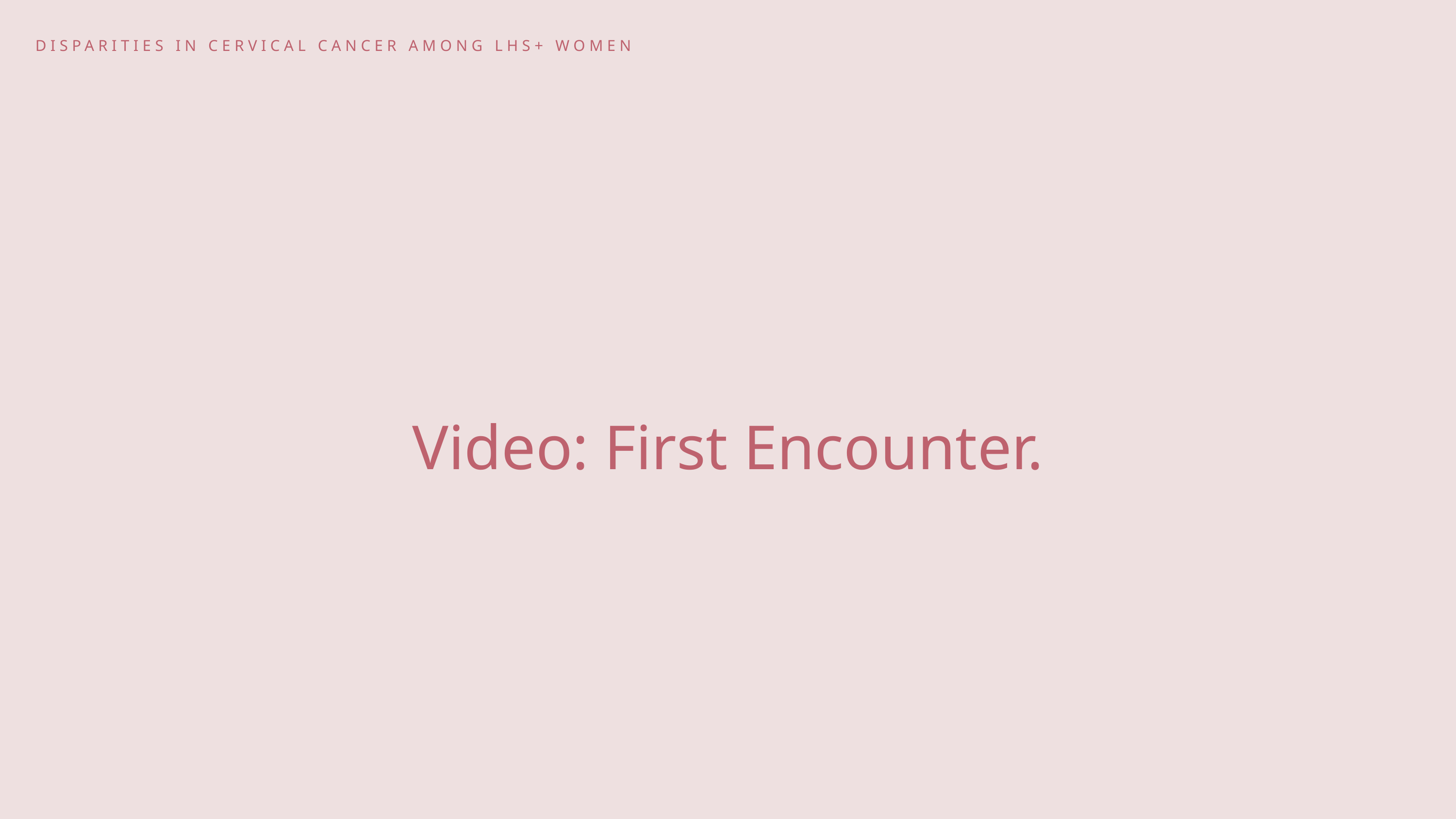

DISPARITIES IN CERVICAL CANCER AMONG LHS+ WOMEN​
Video: First Encounter.

## Slide 31
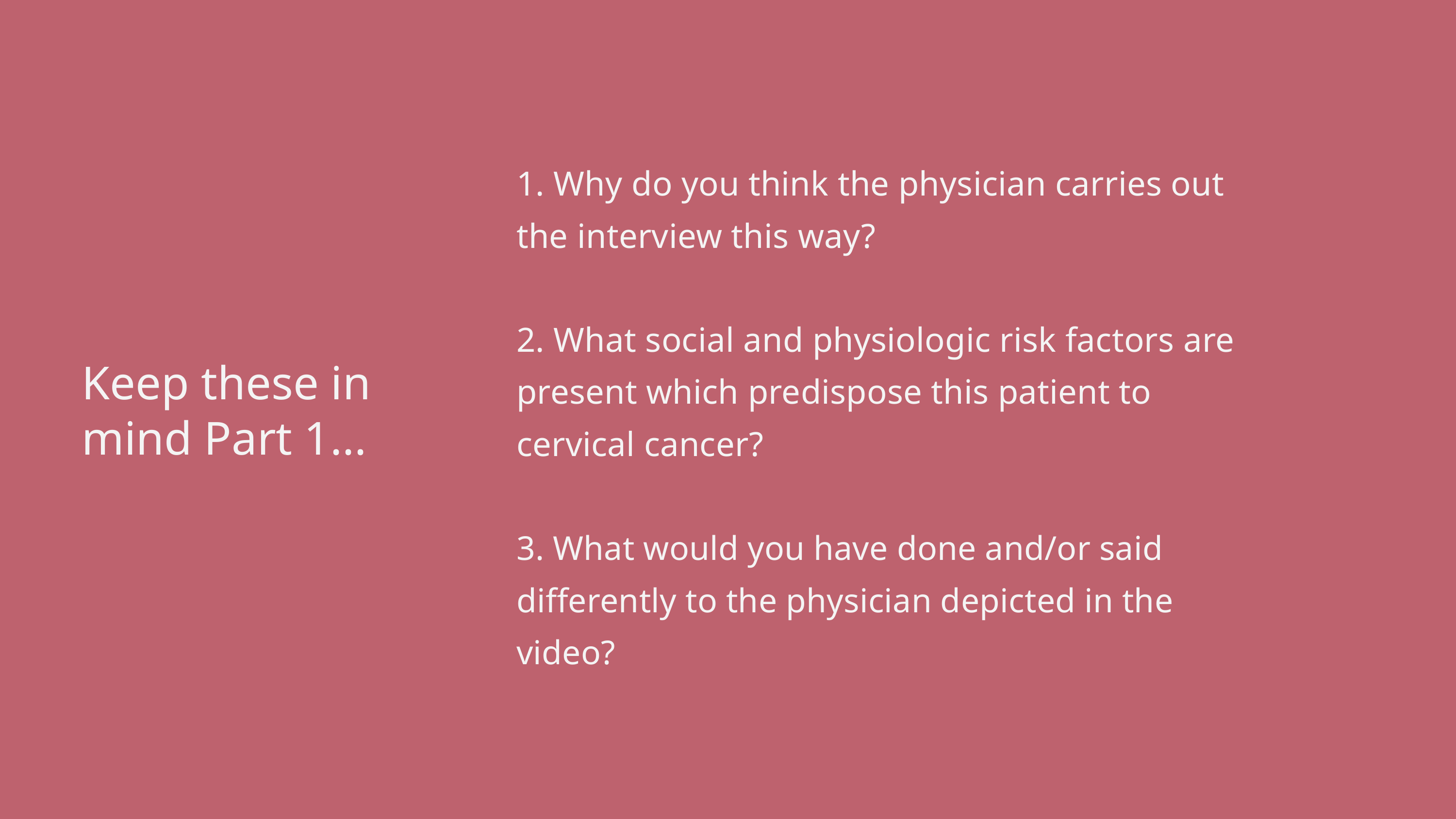

1. Why do you think the physician carries out the interview this way?
2. What social and physiologic risk factors are present which predispose this patient to cervical cancer?​
3. What would you have done and/or said differently to the physician depicted in the video?
Keep these in mind Part 1...

## Slide 32
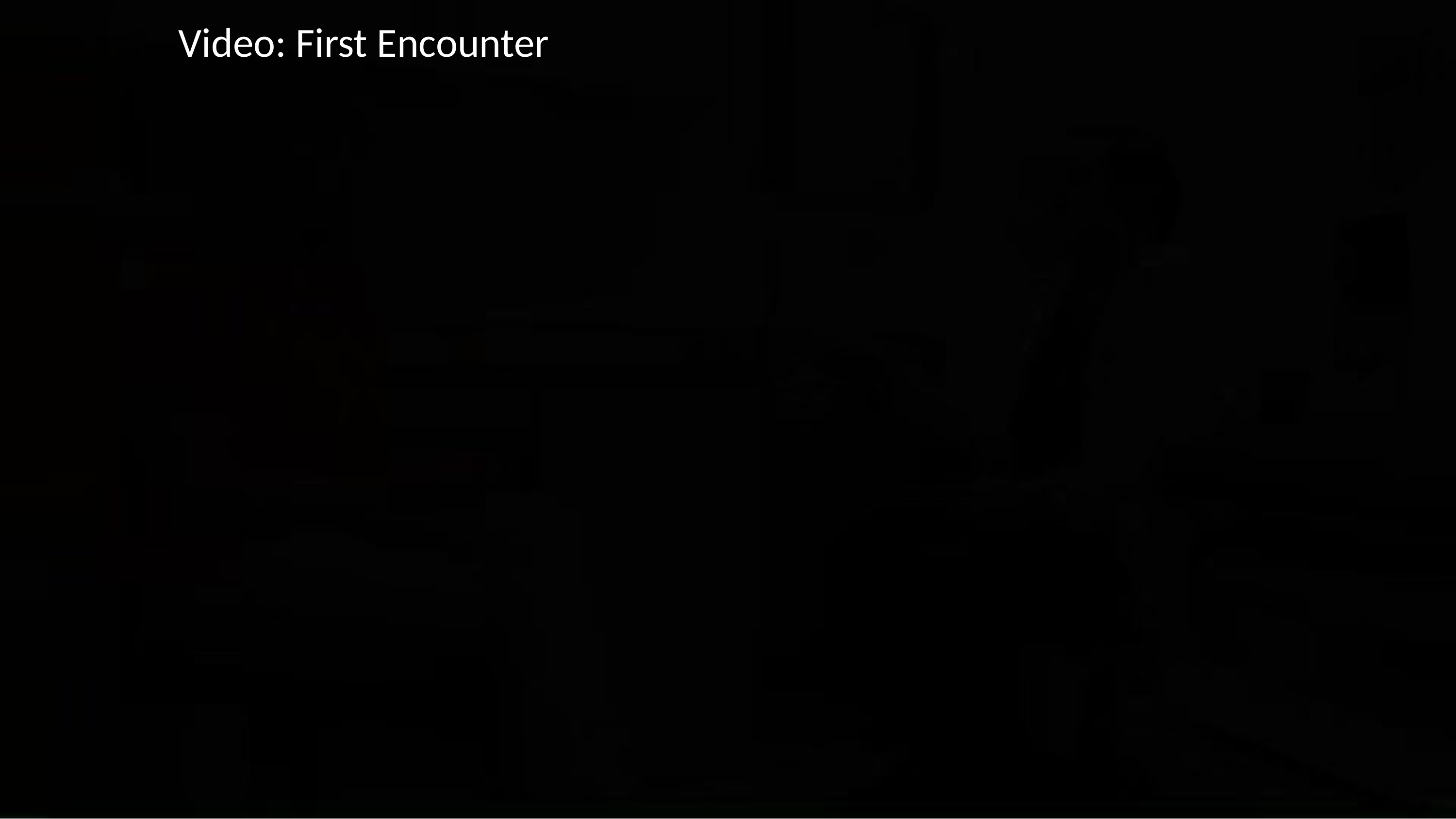

Video: First Encounter

## Slide 33
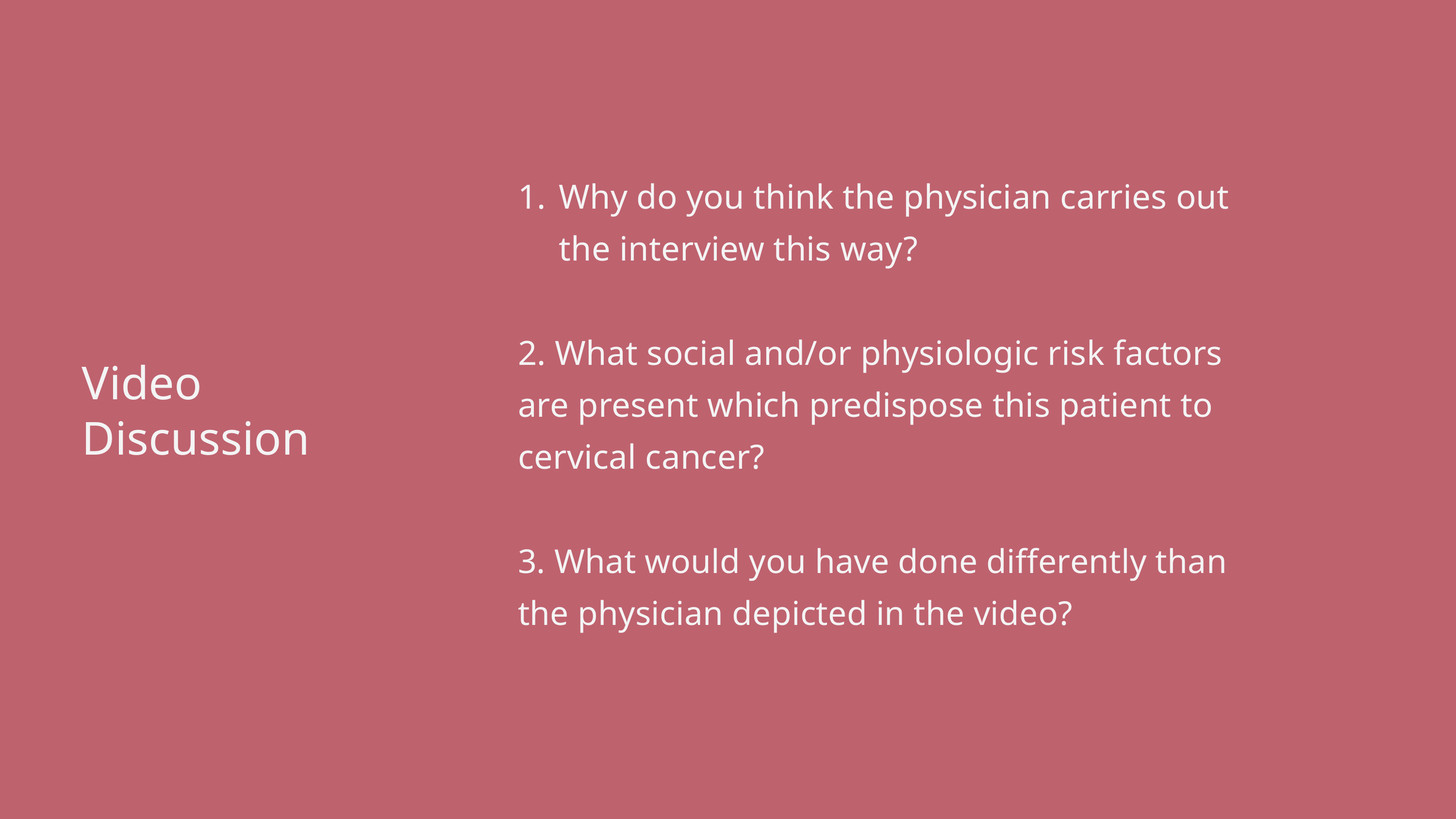

Why do you think the physician carries out the interview this way?
2. What social and/or physiologic risk factors are present which predispose this patient to cervical cancer?
3. What would you have done differently than the physician depicted in the video?
Video Discussion

## Slide 34
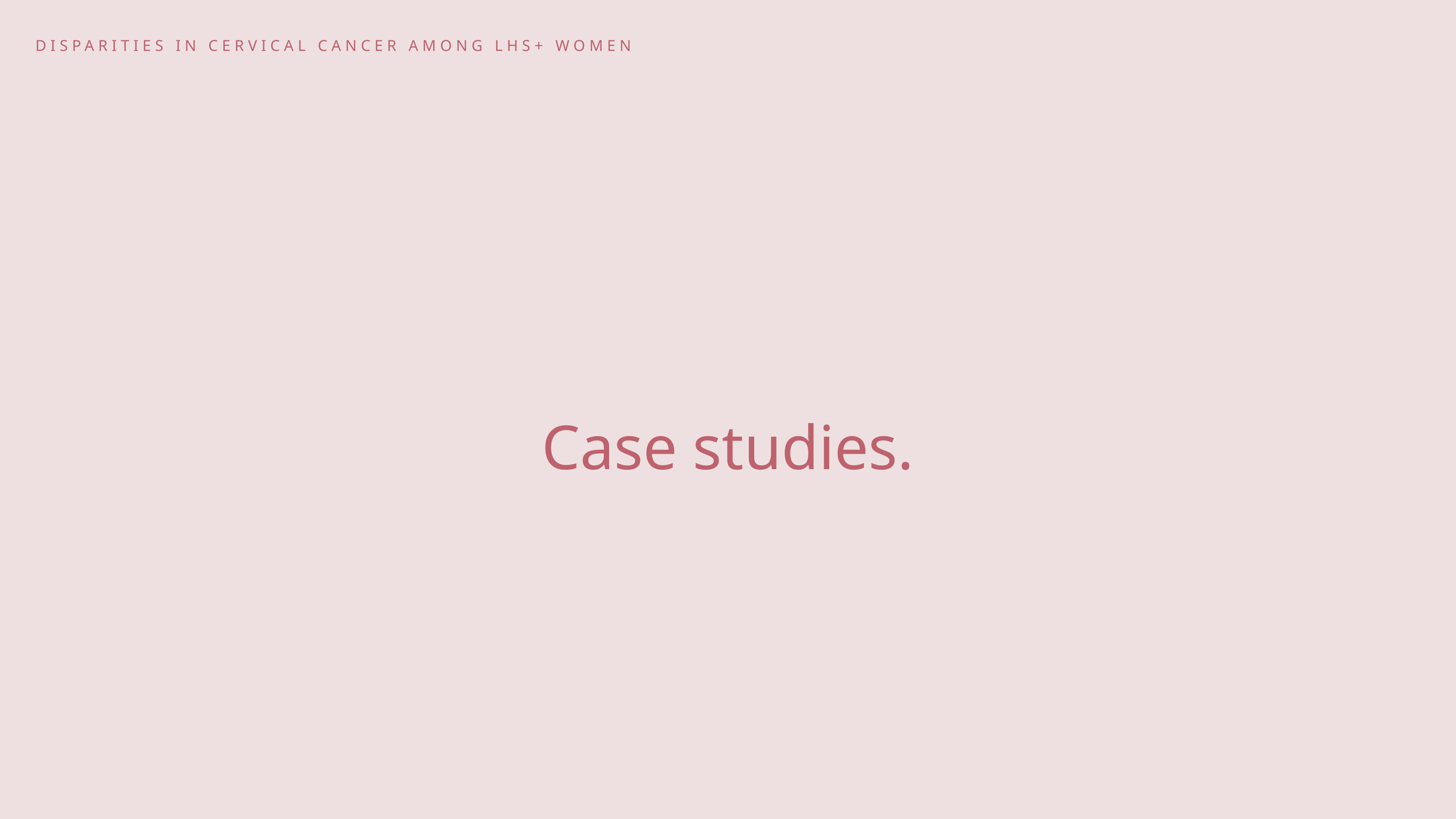

DISPARITIES IN CERVICAL CANCER AMONG LHS+ WOMEN​
Case studies.

## Slide 35
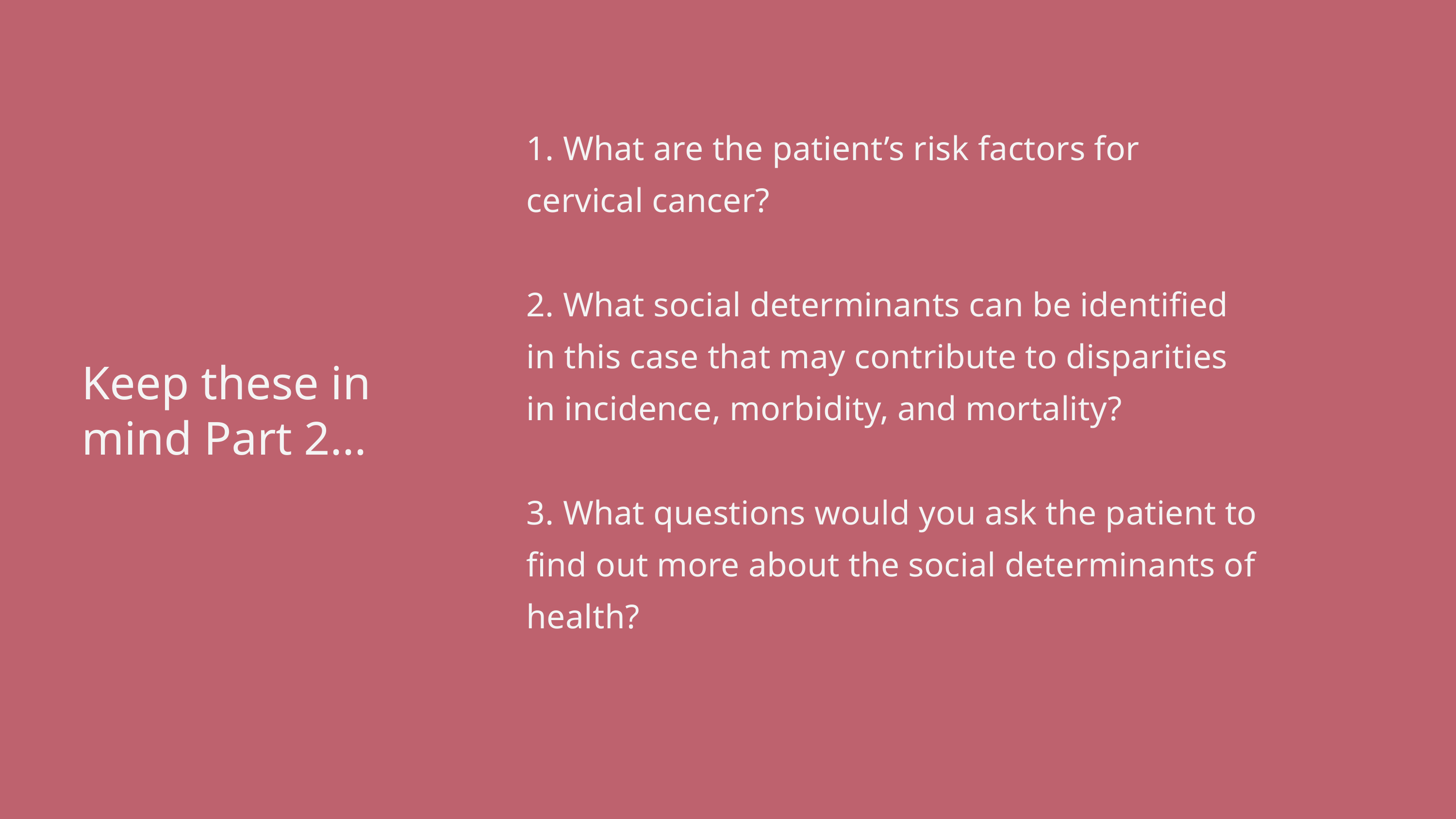

1. What are the patient’s risk factors for cervical cancer?
2. What social determinants can be identified in this case that may contribute to disparities in incidence, morbidity, and mortality?
3. What questions would you ask the patient to find out more about the social determinants of health?
Keep these in mind Part 2...

## Slide 36
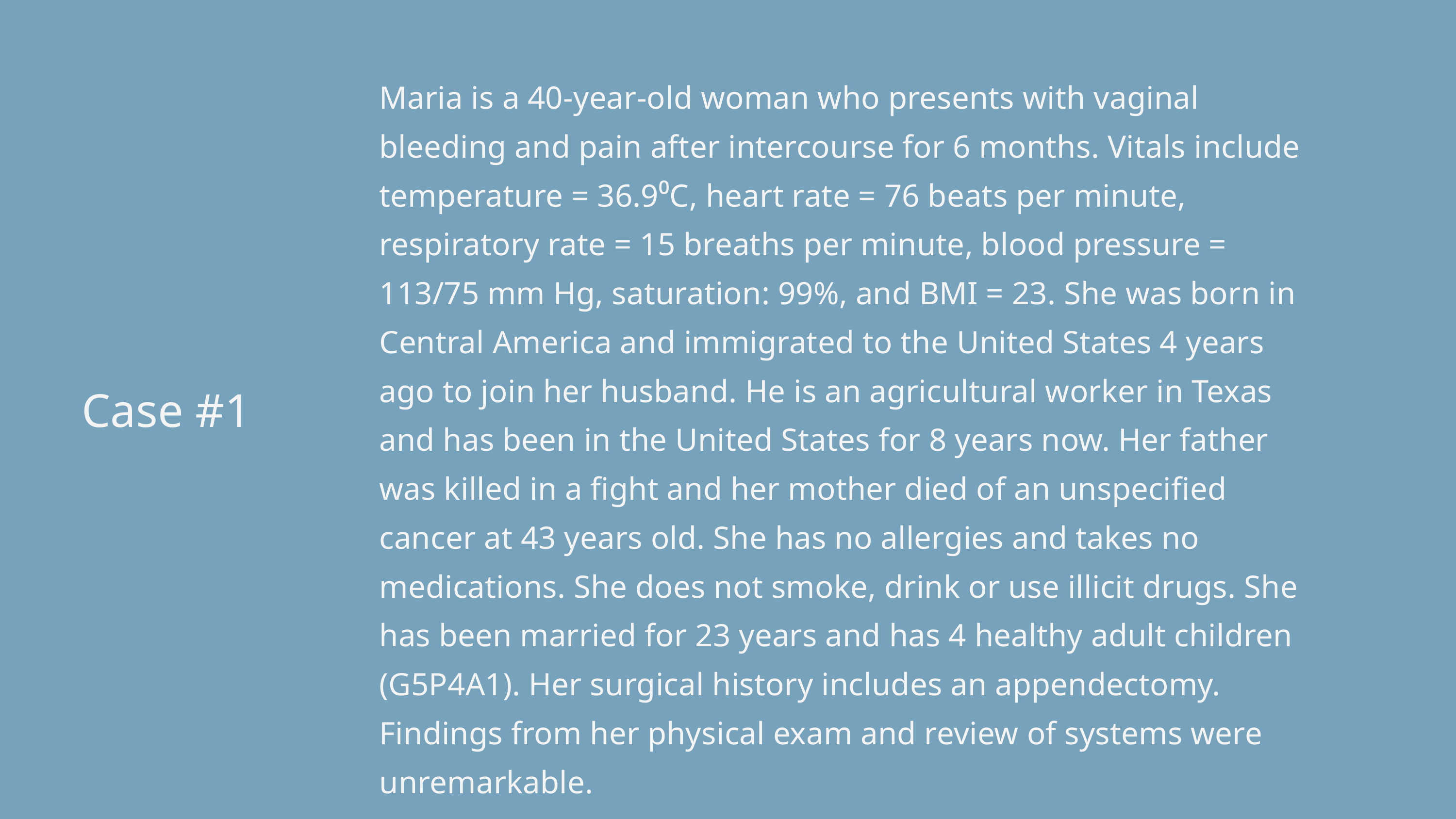

Maria is a 40-year-old woman who presents with vaginal bleeding and pain after intercourse for 6 months. Vitals include temperature = 36.9⁰C, heart rate = 76 beats per minute, respiratory rate = 15 breaths per minute, blood pressure = 113/75 mm Hg, saturation: 99%, and BMI = 23. She was born in Central America and immigrated to the United States 4 years ago to join her husband. He is an agricultural worker in Texas and has been in the United States for 8 years now. Her father was killed in a fight and her mother died of an unspecified cancer at 43 years old. She has no allergies and takes no medications. She does not smoke, drink or use illicit drugs. She has been married for 23 years and has 4 healthy adult children (G5P4A1). Her surgical history includes an appendectomy. Findings from her physical exam and review of systems were unremarkable.
Case #1

## Slide 37
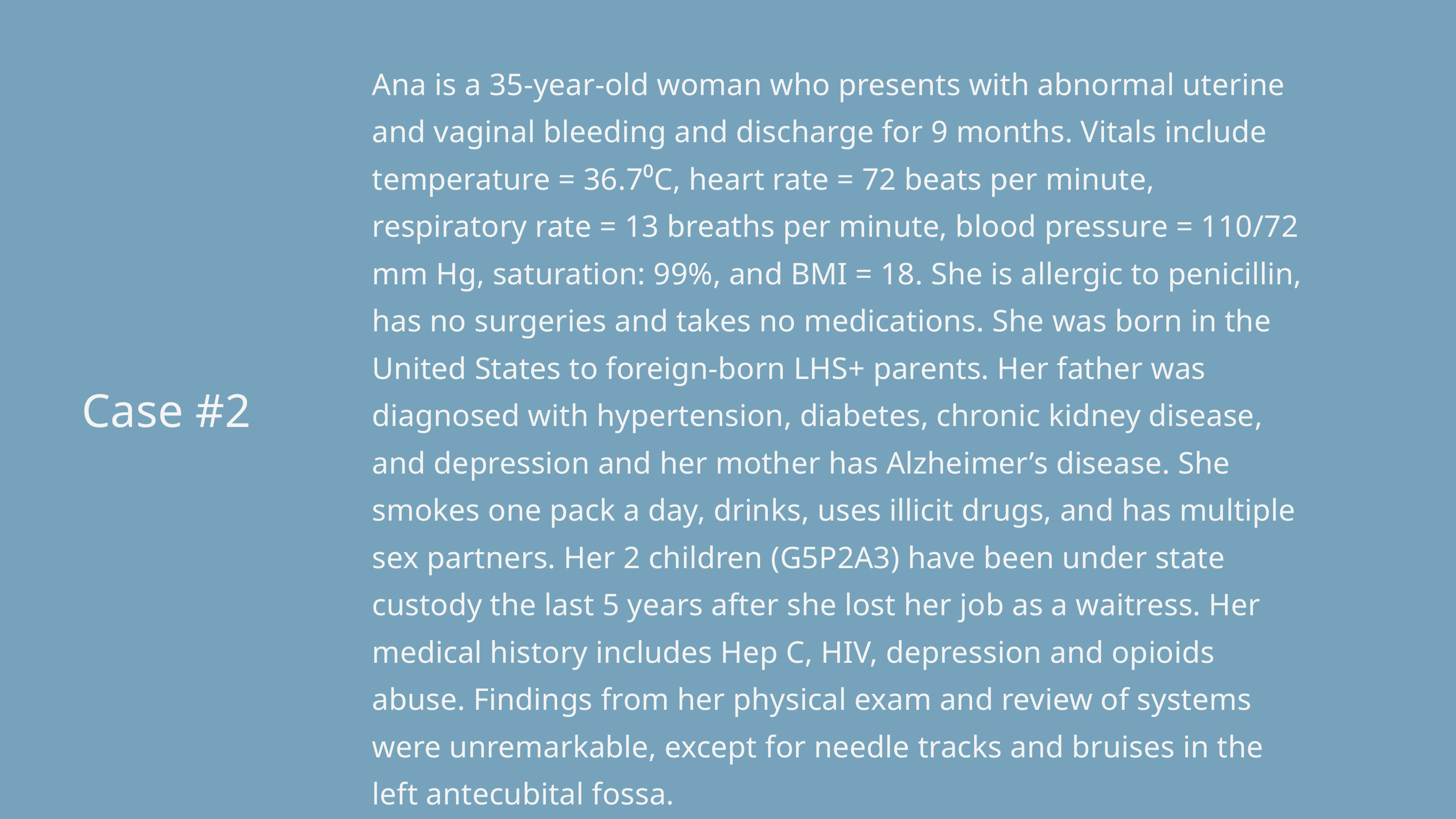

Ana is a 35-year-old woman who presents with abnormal uterine and vaginal bleeding and discharge for 9 months. Vitals include temperature = 36.7⁰C, heart rate = 72 beats per minute, respiratory rate = 13 breaths per minute, blood pressure = 110/72 mm Hg, saturation: 99%, and BMI = 18. She is allergic to penicillin, has no surgeries and takes no medications. She was born in the United States to foreign-born LHS+ parents. Her father was diagnosed with hypertension, diabetes, chronic kidney disease, and depression and her mother has Alzheimer’s disease. She smokes one pack a day, drinks, uses illicit drugs, and has multiple sex partners. Her 2 children (G5P2A3) have been under state custody the last 5 years after she lost her job as a waitress. Her medical history includes Hep C, HIV, depression and opioids abuse. Findings from her physical exam and review of systems were unremarkable, except for needle tracks and bruises in the left antecubital fossa.
Case #2

## Slide 38
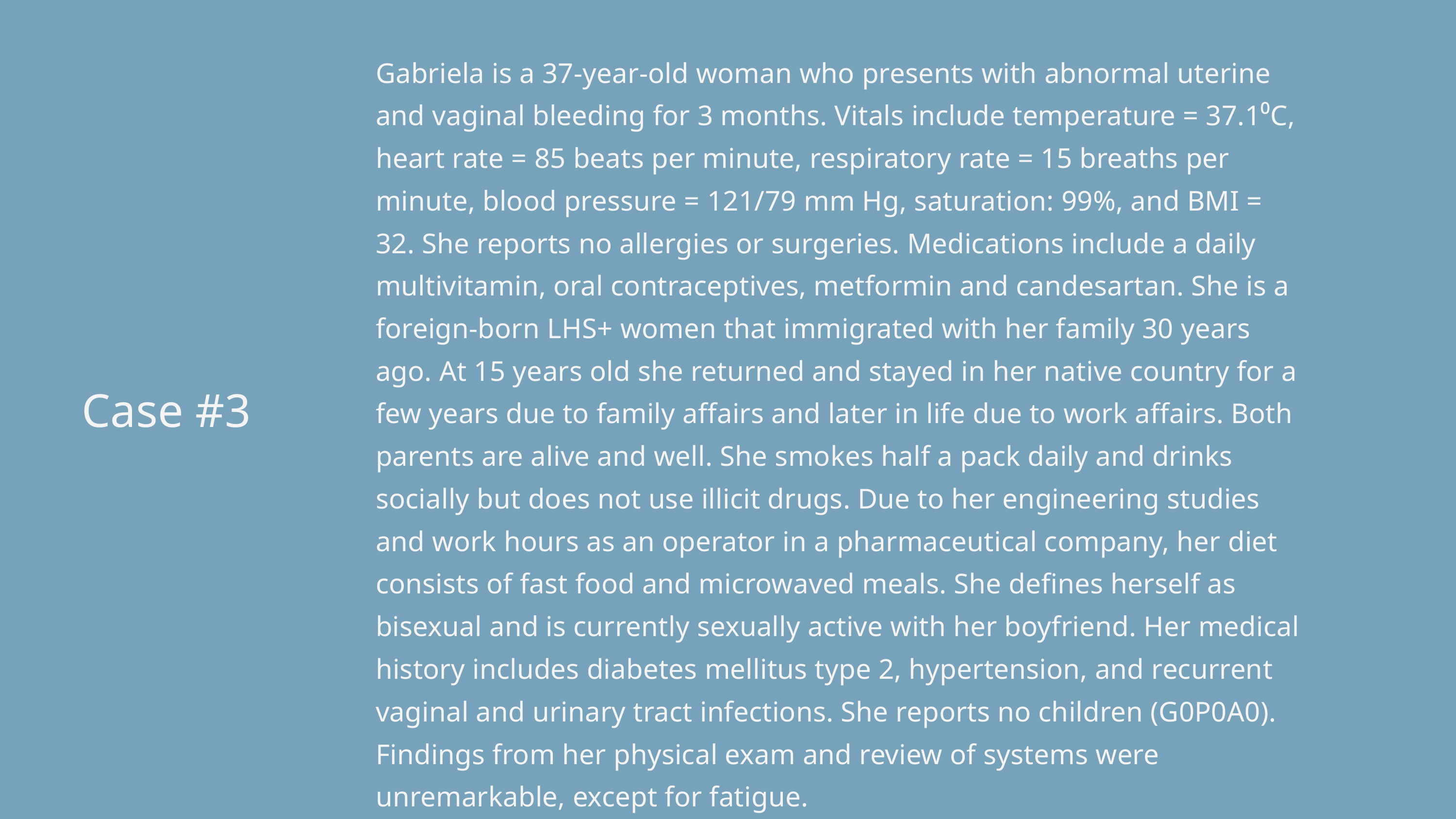

Gabriela is a 37-year-old woman who presents with abnormal uterine and vaginal bleeding for 3 months. Vitals include temperature = 37.1⁰C, heart rate = 85 beats per minute, respiratory rate = 15 breaths per minute, blood pressure = 121/79 mm Hg, saturation: 99%, and BMI = 32. She reports no allergies or surgeries. Medications include a daily multivitamin, oral contraceptives, metformin and candesartan. She is a foreign-born LHS+ women that immigrated with her family 30 years ago. At 15 years old she returned and stayed in her native country for a few years due to family affairs and later in life due to work affairs. Both parents are alive and well. She smokes half a pack daily and drinks socially but does not use illicit drugs. Due to her engineering studies and work hours as an operator in a pharmaceutical company, her diet consists of fast food and microwaved meals. She defines herself as bisexual and is currently sexually active with her boyfriend. Her medical history includes diabetes mellitus type 2, hypertension, and recurrent vaginal and urinary tract infections. She reports no children (G0P0A0). Findings from her physical exam and review of systems were unremarkable, except for fatigue.
Case #3

## Slide 39
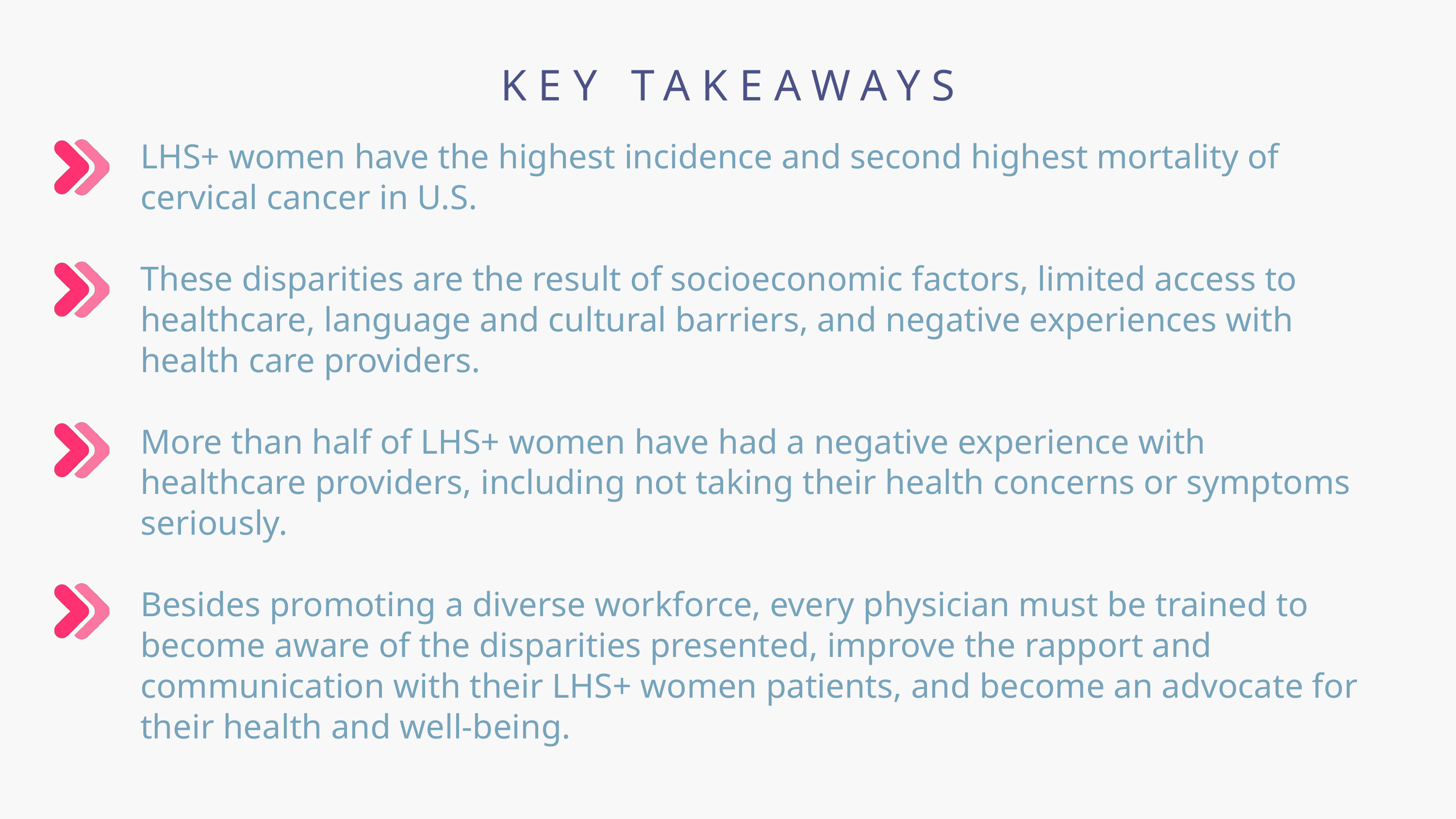

KEY TAKEAWAYS
LHS+ women have the highest incidence and second highest mortality of cervical cancer in U.S.
These disparities are the result of socioeconomic factors, limited access to healthcare, language and cultural barriers, and negative experiences with  health care providers.
More than half of LHS+ women have had a negative experience with healthcare providers, including not taking their health concerns or symptoms seriously.
Besides promoting a diverse workforce, every physician must be trained to become aware of the disparities presented, improve the rapport and communication with their LHS+ women patients, and become an advocate for their health and well-being.

## Slide 40
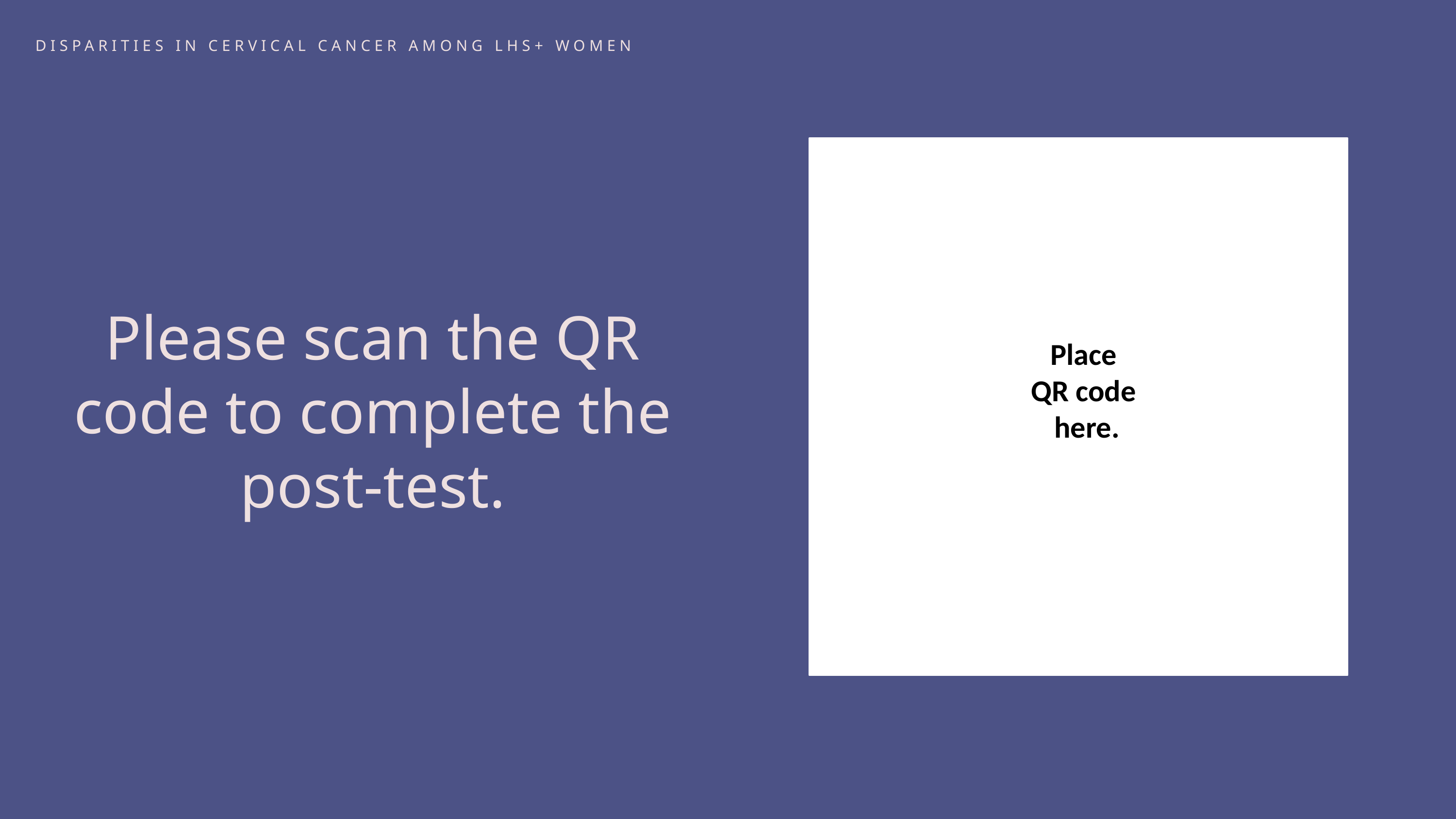

DISPARITIES IN CERVICAL CANCER AMONG LHS+ WOMEN​
Please scan the QR code to complete the post-test.
Place
QR code
here.

## Slide 41
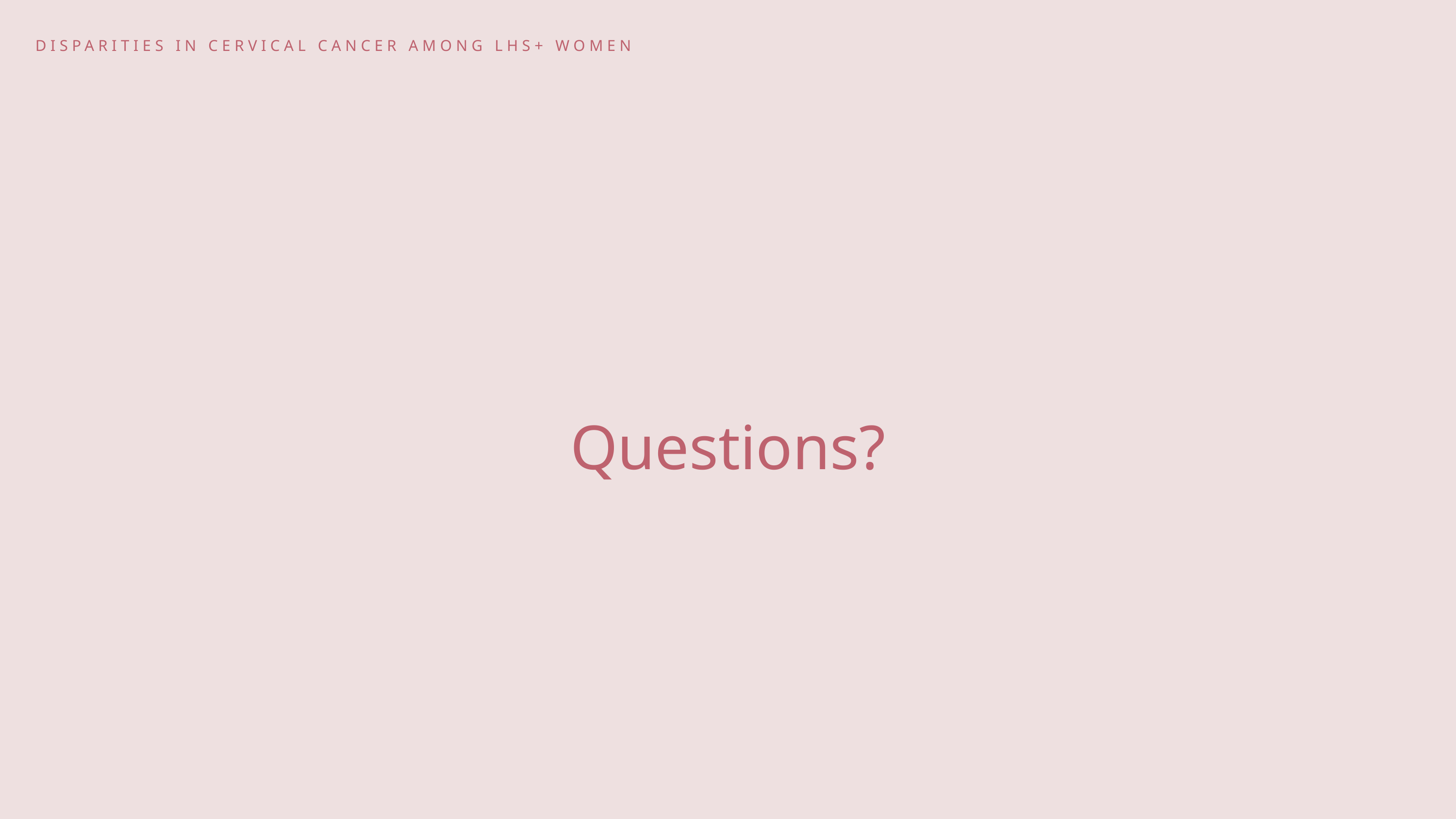

DISPARITIES IN CERVICAL CANCER AMONG LHS+ WOMEN​
Questions?
