# Supplementary material for: Disparities in Cervical Cancer Among LHS+ Women: A Primer for Medical Students
Source: MedEdPORTAL. 2024 Dec 24;20:11482. doi: 10.15766/mep_2374-8265.11482 (PMC11668185; doi:10.15766/mep_2374-8265.11482)
Supplement: Supplementary file 1 — Facilitator Guide.docxPowerPoint Presentation.pptxEvaluation Form.docxVideo.movVideo Script.docxCase Studies.docx [file mep_2374-8265.11482-s001.zip › _Educational Summary Report_11482.pdf]

MedEdPORTAL® The AAMC Journal of Teaching and Learning Resources

Original Publication Open Access

Disparities in Cervical Cancer Among LHS+ Women: A Primer for Medical Students

Alexandra M. Conde Toro, MS, MBA*, Andrea C. Vélez Figueroa, MD†, Alicia Báez Cruz†, Marcel Grau Rodríguez†, Edwin J. Montalvo Rivera†, Diego E. Collazo Irizarry†, Edymarie Vivaldi Marrero†, Pamela Rodríguez Vega†, Alejandra C. Báez Rivera†, Ana I. Calderón Alonso, MPH†, Kaitlyn Pommells, MS†, Álvaro Pérez, MD, MS, MMEL†, John Paul Sánchez, MD, MPH†

*Corresponding author: 121aconde@uccaribe.edu
†Co-second author

Abstract

Introduction: Latina, Latino, Latinx, Latine, Hispanic, or of Spanish origin+ (LHS+) women face higher cervical cancer risks, incidence, and mortality compared to non-Hispanic White women. These disparities are attributable to socioeconomic factors, limited access to health care, language and cultural barriers, and negative health care experiences. **Methods:** We used the Kern model to design, implement, and evaluate a workshop to educate medical students and health care professionals on cervical cancer disparities among LHS+ women and culturally competent communication skills. The workshop included a 60-minute session featuring a PowerPoint presentation, video, and case discussions. **Results:** We conducted the workshop four times, both in person and virtually. We administered pre- and posttests to 46 participants, including medical students and health care professionals. Only 39 participants completed both forms, yielding an 85% response rate. Analysis using the related-samples Wilcoxon signed rank test on responses revealed a significant increase in confidence for each learning objective ($p < .01$). Participants rated the workshop as very good or excellent, and their feedback highlighted the value of interactive activities like the video and case discussions. **Discussion:** Increasing health care providers' awareness of and knowledge about cervical cancer disparities in LHS+ women is essential to improve health care experiences and outcomes. Future workshops should incorporate culturally specific materials for different Spanish-speaking nationalities (e.g., Dominican, Mexican, etc.), medical Spanish training, and cervical cancer education for LGBTQ+ LHS+ women.

Keywords
Cervical Cancer, Latina, Hispanic, Spanish, Women's Health, Cultural Competence, Health Disparities, Health Equity, OB/GYN, Diversity, Equity, Inclusion, Language-Appropriate Health Care

Educational Objectives

By the end of this activity, learners will be able to:

1. Describe cervical cancer epidemiology, risk factors, and prevention efforts.
2. Describe health disparities in cervical cancer among Latina, Latino, Latinx, Latine, Hispanic, or of Spanish origin+ (LHS+) women.
3. Identify the social determinants related to cervical cancer outcomes among LHS+ women.
4. Identify culturally competent communication skills needed to help address barriers in cervical cancer prevention among LHS+ women.

Citation:
Conde Toro AM, Vélez Figueroa AC, Báez Cruz A, et al. Disparities in cervical cancer among LHS+ women: a primer for medical students. *MedEdPORTAL*. 2024;20:11482.
https://doi.org/10.15766/mep_2374-8265.11482

Introduction

Cervical cancer is the fourth most common cancer in women worldwide.[1] In 2020, the incidence rate was 6.8 per 100,000 women in the United States.[2] Differences among race and ethnicity exist, with Latina, Latino, Latinx, Latine, Hispanic or of Spanish origin+ (LHS+) and American Indian and Alaskan Native women having the highest incidence rate (8.4) when compared with Whites (6.5), Blacks (7.3), and Asian and Pacific Islander women (5.4). LHS+ women have an approximately 47% higher risk of cervical cancer incidence and 20% higher risk of death compared to non-Hispanic White (NHW) women.[3]

Copyright © 2024 Conde Toro et al. This is an open-access publication distributed under the terms of the Creative Commons Attribution license.

Risk factors for cervical cancer include smoking, sexual history, obesity, weakened immune system, long-term use of oral contraceptives, multiple full-term pregnancies, diet low in fruits and vegetables, family history of cervical cancer, human papillomavirus (HPV) infection, and lack of HPV vaccination.[4-20] Unfortunately, LHS+ women have higher rates of obesity[14] and HIV[16-19] compared to NHW women. Although LHS+ women smoke less than NHW women, they are less likely to receive cessation counseling when compared to their counterparts.[12,13] Furthermore, HPV vaccination rates are lower in LHS+ adolescents (87%) and adults (36%) compared to NHW counterparts (94% and 45%, respectively).[20]

Disparities in cervical cancer incidence and mortality may result from socioeconomic factors, limited access to health care, and language and cultural barriers.[21-25] A higher percentage of LHS+ individuals do not have a high school diploma (31%), live under the federal poverty level (20%), and lack proficiency in the English language (24%) compared with NHW individuals (7%, 10%, and 11%, respectively).[23] A quarter of LHS+ individuals between 18 and 64 years in the U.S. are uninsured, with a significant gap seen between foreign-born LHS+ individuals (32%) compared to those born in the U.S. (12%). Language and cultural barriers also impact health care by limiting physician-patient communication, care coordination, health education, disease management, and treatment, while increasing length of visits and costs.[24,25]

Approximately half of LHS+ individuals report having had a negative health care experience where they had to speak up to get proper care, felt rushed by their provider, were treated with less respect than other patients, felt their pain was not taken seriously, were looked down on because of weight or eating habits, and thought they received lower quality of care than other patients.[26] Over 30% of LHS+ women report that their health concerns and symptoms were not taken seriously. Younger LHS+ women report more negative health care experiences when compared to older LHS+ women or LHS+ men. Although 58% of LHS+ individuals prefer LHS+ providers, less than 6% of active U.S. providers are LHS+ physicians in a country where 19% of its population identify themselves as Hispanic or Latino.[26-28] Given that LHS+ physicians are scarce, current and future providers must be trained to become aware of their cultural biases, establish rapport with patients from different cultural backgrounds, and develop cultural humility to achieve a successful patient-physician relationship.

This workshop was created to educate medical students and faculty about cervical cancer epidemiology, with a focus on contributory factors and communication strategies to promote prevention. There are only two *MedEdPORTAL* educational publications for medical students related to health disparities and cancer,[29,30] neither of which focuses on educating on disparities in cervical cancer among LHS+ women.

Methods

We used the six-step Kern model to develop, implement, and assess this workshop.[31] In step 1, problem identification and general needs assessment, we conducted a literature review to assess documented training on cervical cancer and LHS+ women. In addition, we incorporated important content from the 2022 Pew Research Center report "Hispanic Americans' Trust in and Engagement With Science," which acted as a needs assessment in the learning environment.[26] In step 2, we held targeted needs assessment and informal discussions with medical students to better understand knowledge gaps. We drafted the learning objectives (step 3) with input from faculty and incorporated Bloom's taxonomy.[32] In step 4, the chosen educational strategies included a PowerPoint presentation along with a video and case discussions for greater engagement by learners. We implemented this workshop four times (step 5), twice in person and twice virtually, targeting students, faculty, and staff from Liaison Committee on Medical Education–accredited medical schools. For step 6, we electronically administered evaluation forms (i.e., pre- and posttests) to assess participants' change in confidence and to gain feedback on workshop design and content.

We, as medical students from the Universidad Central del Caribe School of Medicine (UCCSoM), developed, implemented, and evaluated this workshop. The workshop employed four educational approaches: (1) a collaborative didactic PowerPoint presentation with two knowledge checkpoints included, (2) a video discussion in a large-group format, (3) discussion of three case studies, and (4) a pre- and posttest to evaluate the workshop. No prerequisite knowledge was needed by the participants. The target audience was medical students of any level, but during implementation, we extended the workshop to other health professionals and trainees working with LHS+ women. This project was approved by the Universidad Central del Caribe School of Medicine Institutional Review Board, protocol number 2023-10.

Materials required for this workshop included internet, computer setup for the PowerPoint presentation, connection to a projector, and audiovisual equipment. Participants needed to use their smartphones or tablets to access QR codes and complete pre-

Copyright © 2024 Conde Toro et al. This is an open-access publication distributed under the terms of the Creative Commons Attribution license.

and posttests electronically. A summary of the suggested timeline for this 60-minute workshop is as follows:

- Introduction and pretest (5 minutes, slides 1-5)
- Understanding cervical cancer (5 minutes, slides 6-10)
- HPV and cervical cancer (5 minutes, slides 11-15)
- Disparity factors (10 minutes, slides 16-26)
- Patient-physician communication (10 minutes, slides 27-29)
- Video and discussion (10 minutes, slides 30-33)
- Case studies and discussion (10 minutes, slides 34-38)
- Posttest and key takeaways (5 minutes, slides 39-41)

Using the facilitator guide (Appendix A), we walked through the PowerPoint (Appendix B), beginning with a description of the timeline outlining the contents of the didactic presentation and the relevance of the workshop. After the introduction, we administered the pretest (Appendix C) using a QR code presented on the screen to provide easy access for participants to complete the survey. Then, we focused on the learning objectives, followed by an overview of cervical cancer, including its clinical presentation and risk factors. Subsequent sections highlighted the connection between HPV and cervical cancer, as well as HPV screening and vaccination. Throughout the presentation, there were statistics and facts about disparities in cervical cancer among LHS+ women. The PowerPoint also included information on the importance of a successful physician-patient relationship and how it could be improved with the RESPECT (rapport, empathy, support, partnership, explanations, cultural competence, trust) model.[33]

Then, we presented a video highlighting poor communication between a physician and a patient (Appendix D), followed by a guided, large-group discussion (Appendix A). The video encounter script could be accessed in Appendix E. The video was followed by case studies highlighting three LHS+ women with different risk factors and social determinants of health that might result in cervical cancer disparities (Appendix F). After finishing the case discussions, we instructed the participants to complete the posttest (Appendix C) using another digital QR code presented on the screen. Finally, we shared key takeaways and conducted a Q&A session. Appropriate self-guided learning prepared each facilitator to implement a successful workshop.

A description of the materials implemented to conduct this workshop follows:

- Facilitator guide (Appendix A): This document contained detailed information about each topic covered in the PowerPoint presentation with instructions and talking points to help facilitators present to the audience and guide discussions.
- PowerPoint presentation (Appendix B): The content of the workshop was contained within a 41-slide PowerPoint presentation, which included an outline, pretest QR code, and objectives; general information about cervical cancer, such as clinical presentation and risk factors; facts about HPV's relationship with cervical cancer, screening, and vaccination; statistics and facts about disparities in cervical cancer among LHS+ women, such as socioeconomic factors, language and cultural barriers, health care access, lack of knowledge and awareness, and negative experiences with providers; the importance of a successful physician-patient relationship and how it could be improved with the RESPECT model, which promotes physicians' awareness of their own cultural biases and helps develop rapport with patients from different cultural backgrounds; video and case studies; and discussion questions, posttest QR code, and key takeaways.
- Evaluation form (Appendix C): We developed these documents to evaluate the effectiveness of this workshop, as well as its design and content, following Kirkpatrick's evaluation model.[34] Although the latter includes four levels of criteria (reaction, learning, behavior, and results), in this workshop only the reaction and learning components were assessed. We administered both tests electronically using REDCap, via QR codes included in the PowerPoint presentation. We also assessed participants' confidence aligned with the workshop objectives and overall knowledge of the topics included, as rated on a 5-point Likert-type scale (0 = *no confidence*, 4 = *complete confidence*). A pretest was completed at the beginning of the workshop and a posttest at the end. We instructed participants to provide a unique PIN to pair pre- and posttests without disclosing their identities. Participation was completely voluntary. Completion of pre- and posttests was anonymous and did not include the collection of any personal identifiers.
- Video (Appendix D): This video portrayed the hypothetical scenario of a first medical encounter between a young, bilingual (Spanish-English) woman and a male physician to display some of the dismissive approaches that could result in a negative health care experience and subsequent potential health disparities.
- Video script (Appendix E): This document contained the exact dialogue the characters spoke throughout the video.
- Case studies (Appendix F): This document contained three case studies of hypothetical LHS+ patients to encourage

Copyright © 2024 Conde Toro et al. This is an open-access publication distributed under the terms of the Creative Commons Attribution license.

active participation among the audience by applying gained understanding and knowledge about cervical cancer risk factors, potential barriers that might result in disparities, and communication skills needed to unveil and/or address identified barriers.

We analyzed the data collected using SPSS Statistics Package 29.0. Univariate analyses included frequency and percentage for categorical variables. We used the related-samples Wilcoxon signed rank test to determine if there was a statistically significant difference in responses by participants between the pre- and postworkshop evaluations. Significance was achieved at the level of $p < .01$ for the comparison of each learning objective.

Results

This workshop was implemented four times: two in-person sessions at UCCSoM and the 2023 Latino Medical Student Association (LMSA) National Conference and two virtual sessions using Zoom or Microsoft Teams targeted at LMSA chapters from UCCSoM, University of Puerto Rico School of Medicine, San Juan Bautista School of Medicine, Ponce Health Science University, and University of Illinois College of Medicine. Pre- and posttests were administered to 46 participants, including medical students of any level and other health care professionals and trainees, of whom only 39 completed both forms (85% response rate) and were included in statistical analysis.

Self-assessment of participants regarding their confidence in their ability to meet the workshop's learning objectives ranged from no confidence (0) to complete confidence (4). Analysis of pre- and posttest responses to confidence in meeting each learning objective showed a statistically significant ($p < .01$) increase in confidence for each of the four learning objectives (Table 1).

Results of descriptive questions assessing the participants' knowledge demonstrated improvement in the average score between the pre- and posttests (Table 2).

All 39 respondents rated the workshop as very good or excellent. Thirty-eight participants commented that what they appreciated most about the workshop was the focus on cervical cancer disparities by different racial and ethnic groups (six responses) and the inclusion of data/statistics (five responses). The workshop was considered interactive (eight responses), engaging (three responses), and well organized. The attendees valued the workshop's incorporation of the video (five responses), the case discussions (three responses), and the RESPECT model to facilitate better patient-physician communication (two responses). One respondent commented, "The video was a great visual aid to demonstrate common real-life scenarios, and includes multiple microaggressions, several of which border on outright aggressive behavior."

Thirty-eight individuals provided qualitative feedback about what should be done to improve the workshop. Although the most frequent written response was "no changes" (10 responses), three individuals commented on the importance of spending more time or sufficient time on the case scenarios. Some additional singular comments included providing LGBTQ-related information, medical Spanish terminology for counseling on HPV vaccines, and more images of cervical cancer.

Discussion

This workshop was developed to educate health trainees and professionals on how social determinants affecting LHS+ women in the U.S. result in cervical cancer disparities, as well as on what providers can do as individuals to improve their communication

Table 1. Median Response to Pre- and Postworkshop Confidence Questions ($N = 39$)

Objective[a]	*Mdn* Pre	Post
Please rate how much CONFIDENCE you have in your ability to:		
Objective 1: Describe cervical cancer epidemiology, risk factors, and prevention efforts.	2	3
Objective 2: Describe health disparities in cervical cancer among LHS+ women.	2	3
Objective 3: Identify the social determinants related to cervical cancer outcomes among LHS+ women.	2	3
Objective 4: Identify culturally competent communication skills needed to help address barriers in cervical cancer prevention among LHS+ women.	2	3

Abbreviation: LHS+, Latina, Latino, Latinx, Latine, Hispanic, or of Spanish origin+.
[a]The related-samples Wilcoxon signed rank test was used to determine if there was a statistically significant difference in responses by participants between the pre- and postworkshop evaluations. Significance was achieved at the level of $p < .01$ for the comparison of each learning objective.

Table 2. Mean Percentage Correct for Pre- and Postworkshop Knowledge Questions ($N = 39$)

Question (Correct Answer in Parentheses)	% Correct Pre	Post
Which of the following is not a risk factor for cervical cancer? (Family history of breast cancer)	6	67
The human papillomavirus vaccine is licensed in the United States for women and men through age (45) years.	35	54
Which of the following *does not* result in cervical cancer disparities among LHS+ women? (Criminality rates)	74	83
Cultural barriers among LHS+ women include all the following, except: (Distance)	48	65
The RESPECT model is a widely used tool to (promote physicians' awareness of their own cultural biases and develop rapport with patients from diverse cultural backgrounds).	59	72

Abbreviations: LHS+, Latina, Latino, Latinx, Latine, Hispanic, or of Spanish origin+; RESPECT, rapport, empathy, support, partnership, explanations, cultural competence, trust.

Copyright © 2024 Conde Toro et al. This is an open-access publication distributed under the terms of the Creative Commons Attribution license.

and relationships with patients. The workshop featured teaching materials that enhance medical education and, subsequently, the care, quality, experience, and health outcomes of this diverse community. Objectives were to describe cervical cancer epidemiology, risk factors, and prevention efforts; describe health disparities in cervical cancer among LHS+ women and identify related social determinants; and recognize culturally competent communication skills needed to assess barriers in cervical cancer prevention.

The overall results—quantitative and qualitative data—were positive. The workshop objectives were achieved to varying degrees. This was shown by an increase in knowledge through an examination of multiple-choice questions answered correctly. It is important to note that an increase in confidence does not indicate the ability to correctly perform all the tasks outlined in the objectives, as assessed through a subjective self-evaluation.

Participants' feedback highlighted the value of incorporating interactive and engaging activities such as video and case study discussions. Time allotment varied between sessions due to implementation modality (i.e., in person or virtual) and location (e.g., conference, school, etc.). After the first implementation, we reduced the amount of time spent on understanding cervical cancer and HPV from 15 to 10 minutes to increase the time allotment for the video and case study discussions. Several participants appreciated the up-to-date statistics about cervical cancer disparities. Facilitators should consider reviewing and revising the statistics before implementation.

Opportunities for improvement recommended by participants included discussing statistics and information about LHS+ women from the LGBTQ+ community, emphasizing pre- and posttest answers more during sessions, facilitating a discussion and/or suggesting strategies to address identified barriers and bridge gaps to care, and using the RESPECT model to exemplify how to educate an LHS+ patient on HPV vaccination.

There are many limitations to this workshop. It was developed for implementation in English. A module in Spanish may further prepare practitioners to communicate more effectively with Spanish-speaking LHS+ women. The evaluation form did not include questions on participants' demographic characteristics, thus limiting a stratified analysis by race and ethnicity, professional role or year in training, or specialty. The workshop was presented in both virtual and in-person formats. The facilitators observed increased participation and engagement during the case discussions in the in-person format compared to a hesitance to unmute in the virtual format. Thus, participants were more likely to contribute to the discussion and engage with each other when the workshop was presented in person. The workshop was a brief intervention and was focused on awareness of the topic and on attaining new knowledge. It was not designed to show sustained improvement in awareness or knowledge or to demonstrate behavior change.

Further interventions aimed to educate health trainees and professionals about cervical cancer disparities in LHS+ women may consider exploring disparities in LGBTQ+ patients and between subpopulations of LHS+ women (i.e., races and ethnicities), as well as the impact of HPV vaccination rates in males on women's health.

Appendices

A. Facilitator Guide.docx

B. PowerPoint Presentation.pptx

C. Evaluation Form.docx

D. Video.mov

E. Video Script.docx

F. Case Studies.docx

All appendices are peer reviewed as integral parts of the Original Publication.

Alexandra M. Conde Toro, MS, MBA: Fourth-Year Medical Student, Universidad Central del Caribe School of Medicine; ORCID: https://orcid.org/0009-0008-4925-6193

Andrea C. Vélez Figueroa, MD: First-Year Resident, Hospital Universitario Dr. Ramón Ruiz Arnau and Universidad Central del Caribe School of Medicine

Alicia Báez Cruz: Fourth-Year Medical Student, Universidad Central del Caribe School of Medicine

Marcel Grau Rodríguez: Third-Year Medical Student, Universidad Central del Caribe School of Medicine

Edwin J. Montalvo Rivera: Fourth-Year Medical Student, Universidad Central del Caribe School of Medicine

Diego E. Collazo Irizarry: Fourth-Year Medical Student, Universidad Central del Caribe School of Medicine

Edymarie Vivaldi Marrero: Fourth-Year Medical Student, Universidad Central del Caribe School of Medicine

Pamela Rodríguez Vega: Third-Year Medical Student, Universidad Central del Caribe School of Medicine

Alejandra C. Báez Rivera: Third-Year Medical Student, Universidad Central del Caribe School of Medicine

Copyright © 2024 Conde Toro et al. This is an open-access publication distributed under the terms of the Creative Commons Attribution license.

Ana I. Calderón Alonso, MPH: Third-Year Medical Student, Universidad Central del Caribe School of Medicine

Kaitlyn Pommells, MS: Coordinator, Academic Medicine Writing Fellowship, Building the Next Generation of Academic Physicians

Álvaro Pérez, MD, MS, MMEL: Assistant Dean for Curriculum Development, Accreditation, and Licensing, Universidad Central del Caribe School of Medicine

John Paul Sánchez, MD, MPH: Dean, Universidad Central del Caribe School of Medicine

Acknowledgments
This project was supported by the National Board of Medical Examiners, the Academic Medicine Writing Fellowship of Building the Next Generation of Academic Physicians, and the Latino Medical Student Association.

Disclosures
None to report.

Funding/Support
None to report.

Prior Presentations
Conde Toro AM, Vélez Figueroa AC, Báez Cruz A, et al. Disparities in cervical cancer among LHS+ women: a primer for medical students. Poster presented at: 2024 Advancing the Science of Cancer in Latinos; February 21, 2024; San Antonio, TX.

Conde Toro AM, Vélez Figueroa AC, Báez Cruz A, et al. Disparities in cervical cancer among LHS+ women: a primer for medical students. Poster presented at: 2024 Latino Medical Student Association Southeast Regional Conference; February 24, 2024; Charleston, SC.

Conde Toro AM, Vélez Figueroa AC, Báez Cruz A, et al. Disparities in cervical cancer among LHS+ women: a primer for medical students. Poster presented at: 44th Annual Research and Education Forum of the University of Puerto Rico, Medical Science Campus; March 15, 2024; San Juan, PR.

Conde Toro AM, Vélez Figueroa AC, Báez Cruz A, et al. Disparities in cervical cancer among LHS+ women: a primer for medical students. Poster presented at: 2nd Latino Medical Student Association Annual Research Symposium Forum of the University of Puerto Rico, Medical Science Campus; April 20, 2024; San Juan, PR.

Ethical Approval
The Universidad Central del Caribe School of Medicine Institutional Review Board approved this project.

References

1. Cervical cancer. World Health Organization. March 5, 2024. Accessed November 27, 2024. https://www.who.int/news-room/fact-sheets/detail/cervical-cancer
2. U.S. Cancer Statistics Working Group. U.S. cancer statistics data visualizations tool. Centers for Disease Control and Prevention/National Cancer Institute. June 2024. Accessed November 27, 2024. https://gis.cdc.gov/Cancer/USCS/#/Demographics/
3. Williams PA, Zaidi SK, Sengupta R. AACR Cancer Disparities Progress Report 2022. *Cancer Epidemiol Biomarkers Prev*. 2022;31(7):1249-1250. https://doi.org/10.1158/1055-9965.EPI-22-0542
4. *Cervical Cancer Causes, Risk Factors, and Prevention*. American Cancer Society; 2020. Accessed November 27, 2024. https://www.cancer.org/content/dam/CRC/PDF/Public/8600.00.pdf
5. Kashyap N, Krishnan N, Kaur S, Ghai S. Risk factors of cervical cancer: a case-control study. *Asia Pac J Oncol Nurs*. 2019;6(3):308-314. https://doi.org/10.4103/apjon.apjon_73_18
6. Gadducci A, Cosio S, Fruzzetti F. Estro-progestin contraceptives and risk of cervical cancer: a debated issue. *Anticancer Res*. 2020;40(11):5995-6002. https://doi.org/10.21873/anticanres.14620
7. Oral contraceptives and cancer risk. National Cancer Institute. Updated February 22, 2018. Accessed November 27, 2024. https://www.cancer.gov/about-cancer/causes-prevention/risk/hormones/oral-contraceptives-fact-sheet#:~:text=In%20addition%2C%20oral%20contraceptives%20might,of%20virtually%20all%20cervical%20cancers
8. Hinkula M, Pukkala E, Kyyrönen P, et al. A population-based study on the risk of cervical cancer and cervical intraepithelial neoplasia among grand multiparous women in Finland. *Br J Cancer*. 2004;90(5):1025-1029. https://doi.org/10.1038/sj.bjc.6601650
9. Atalah E, Urteaga C, Rebolledo A, Villegas RA, Medina E, Csendes A. Alimentación, tabaquismo e historia reproductiva como factores de riesgo del cáncer de cuello del útero. Diet, smoking and reproductive history as risk factors for cervical cancer. *Rev Med Chil*. 2001;129(6):597-603. https://doi.org/10.4067/S0034-98872001000600001
10. Hajiesmaeil M, Mirzaei Dahka S, Khorrami R, et al. The association between dietary intake and cervical cancer in women at risk of cervical cancer: a nested case-control study. *Caspian J Intern Med*. 2022;13(3):599-606. https://doi.org/10.22088/cjim.13.3.599
11. Zelmanowicz AdeM, Hildesheim A. Family history of cancer as a risk factor for cervical carcinoma: a review of the literature. *Papillomavirus Rep*. 2004;15(3):113-120. https://doi.org/10.1179/095741904225005115
12. Kristman-Valente AN, Flaherty BP. Latino cigarette smoking patterns by gender in a US-national sample. *Ann Behav Med*. 2016;50(1):34-47. https://doi.org/10.1007/s12160-015-9729-9
13. Hispanic and Latino people encounter barriers to quitting successfully. Centers for Disease Control and Prevention. May 15, 2024. Accessed November 27, 2024.

Copyright © 2024 Conde Toro et al. This is an open-access publication distributed under the terms of the Creative Commons Attribution license.

https://www.cdc.gov/tobacco-health-equity/collection/hispanic-latino-quitting-tobacco.html?CDC_AAref_Val=https://www.cdc.gov/tobacco/health-equity/hispanic-latino/quitting-tobacco.html

14. Fryar CD, Carroll MD, Ogden CL; Division of Health and Nutrition Examination Surveys. *Prevalence of Overweight, Obesity, and Severe Obesity Among Adults Aged 20 and Over: United States, 1960–1962 Through 2015–2016*. National Center for Health Statistics; 2018. Accessed November 27, 2024. https://www.cdc.gov/nchs/data/hestat/obesity_adult_15_16/obesity_adult_15_16.pdf

15. Maruthur NM, Bolen SD, Brancati FL, Clark JM. The association of obesity and cervical cancer screening: a systematic review and meta-analysis. *Obesity (Silver Spring)*. 2009;17(2):375-381. https://doi.org/10.1038/oby.2008.480

16. Salihu H, Dongarwar D, Ikedionwu CA, et al. Racial/ethnic disparities in the burden of HIV/cervical cancer comorbidity and related in-hospital mortality in the USA. *J Racial Ethn Health Disparities*. 2021;8(1):24-32. https://doi.org/10.1007/s40615-020-00751-5

17. Ortiz AP, Engels EA, Nogueras-González GM, et al. Disparities in human papillomavirus–related cancer incidence and survival among human immunodeficiency virus–infected Hispanics living in the United States. *Cancer*. 2018;124(23):4520-4528. https://doi.org/10.1002/cncr.31702

18. Peragallo N, Gonzalez-Guarda RM, McCabe BE, Cianelli R. The efficacy of an HIV risk reduction intervention for Hispanic women. *AIDS Behav*. 2012;16(5):1316-1326. https://doi.org/10.1007/s10461-011-0052-6

19. Berman TA, Schiller JT. Human papillomavirus in cervical cancer and oropharyngeal cancer: one cause, two diseases. *Cancer*. 2017;123(12):2219-2229. https://doi.org/10.1002/cncr.30588

20. Immunizations and Hispanic Americans. Department of Health and Human Services Office of Minority Health. Updated October 29, 2024. Accessed November 27, 2024. https://minorityhealth.hhs.gov/immunizations-and-hispanic-americans

21. Buskwofie A, David-West G, Clare CA. A review of cervical cancer: incidence and disparities. *J Natl Med Assoc*. 2020;112(2):229-232. https://doi.org/10.1016/j.jnma.2020.03.002

22. Garner EIO. Cervical cancer: disparities in screening, treatment, and survival. *Cancer Epidemiol Biomarkers Prev*. 2003;12(3):242s-247s.

23. Miller KD, Ortiz AP, Pinheiro PS, et al. Cancer statistics for the US Hispanic/Latino population, 2021. *CA Cancer J Clin*. 2021;71(6):466-487. https://doi.org/10.3322/caac.21695

24. Escobedo LE, Cervantes L, Havranek E. Barriers in healthcare for Latinx patients with limited English proficiency—a narrative review. *J Gen Intern Med*. 2023;38(5):1264-1271. https://doi.org/10.1007/s11606-022-07995-3

25. Al Shamsi H, Almutairi AG, Al Mashrafi S, Al Kalbani T. Implications of language barriers for healthcare: a systematic review. *Oman Med J*. 2020;35(2):e122. https://doi.org/10.5001/omj.2020.40

26. Funk C, Lopez MH. Hispanic Americans' trust in and engagement with science. Pew Research Center. June 14, 2022. Accessed November 27, 2024. https://www.pewresearch.org/science/2022/06/14/hispanic-americans-trust-in-and-engagement-with-science/

27. QuickFacts. United States Census Bureau. Accessed November 27, 2024. https://www.census.gov/quickfacts/fact/table/US/RHI725222

28. Diversity in medicine: facts and figures 2019. Association of American Medical Colleges. Accessed November 27, 2024. https://www.aamc.org/data-reports/workforce/data/figure-18-percentage-all-active-physicians-race/ethnicity-2018#:~:text=Diversity%20in%20Medicine%3A%20Facts%20and%20Figures%202019,-Diversity%20in%20Medicine&text=Figure%2018%20shows%20the%20percentage,as%20Black%20or%20African%20American

29. Van Schaik E, Howson A, Sabin J. Healthcare disparities. *MedEdPORTAL*. 2014;10:9675. https://doi.org/10.15766/mep_2374-8265.9675

30. Martinez IL, Ilangovan K, Whisenant EB, Pedoussaut M, Lage OG. Breast health disparities: a primer for medical students. *MedEdPORTAL*. 2016;12:10471. https://doi.org/10.15766/mep_2374-8265.10471

31. Thomas PA, Kern DE, Hughes MT, Tackett SA, Chen BY, eds. *Curriculum Development for Medical Education: A Six-Step Approach*. 4th ed. Johns Hopkins University Press; 2022.

32. Anderson LW, Krathwohl DR, eds. *A Taxonomy for Learning, Teaching, and Assessing: A Revision of Bloom's Taxonomy of Educational Objectives*. Longman; 2001.

33. Mostow C, Crosson J, Gordon S, et al. Treating and precepting with RESPECT: a relational model addressing race, ethnicity, and culture in medical training. *J Gen Intern Med*. 2010;25(suppl 2):146-154. https://doi.org/10.1007/s11606-010-1274-4

34. Kirkpatrick JD, Kirkpatrick WK. *Kirkpatrick's Four Levels of Training Evaluation*. ATD Press; 2016.

Received: January 27, 2024
Accepted: October 1, 2024
Published: December 24, 2024

Copyright © 2024 Conde Toro et al. This is an open-access publication distributed under the terms of the Creative Commons Attribution license.
